# Mindfulness Training vs Recovery Support for Opioid Use, Craving, and Anxiety During Buprenorphine Treatment: A Randomized Clinical Trial

**DOI:** 10.1001/jamanetworkopen.2024.54950

**Published:** 2025-01-21

**Authors:** Zev Schuman-Olivier, Hannah Goodman, Joseph Rosansky, Alaine Kiera Fredericksen, Javier Barria, Gareth Parry, Randi Sokol, Paula Gardiner, Benjamin Lê Cook, Roger D. Weiss

**Affiliations:** 1 Department of Psychiatry, Cambridge Health Alliance, Malden, Massachusetts; 2 Department of Psychiatry, Harvard Medical School, Boston, Massachusetts; 3 Department of Family Medicine, Cambridge Health Alliance, Malden, Massachusetts; 4 Tufts University School of Medicine, Boston, Massachusetts; 5 Division of Alcohol, Drugs, and Addiction, McLean Hospital, Belmont, Massachusetts

## Abstract

**Importance:**

During buprenorphine treatment for opioid use disorder (OUD), risk factors for opioid relapse or treatment dropout include comorbid substance use disorder, anxiety, or residual opioid craving. There is a need for a well-powered trial to evaluate virtually delivered groups, including both mindfulness and evidence-based approaches, to address these comorbidities during buprenorphine treatment.

**Objective:**

To compare the effects of the Mindful Recovery Opioid Use Disorder Care Continuum (M-ROCC) vs active control among adults receiving buprenorphine for OUD.

**Design, Setting, and Participants:**

This randomized clinical trial was conducted from January 21, 2021, to September 19, 2023. All study procedures were conducted virtually. Participants were randomized 1:1 and blinded to intervention assignments throughout participation. This trial recruited online from 16 US states and was conducted via online platforms. Patients prescribed buprenorphine for OUD were recruited via social media advertisements, flyers, and health care professional referrals.

**Interventions:**

The M-ROCC program was a 24-week, motivationally adaptive, trauma-informed, mindfulness-based group curriculum. Participants attended a 30-minute informal check-in and 60-minute intervention group each week. The recovery support group control curriculum used 4 evidence-based substance use disorder–focused nonmindfulness approaches and was time and attention matched.

**Main Outcomes and Measures:**

The primary outcome was the number of 2-week periods with both self-reported and biochemically confirmed abstinence from illicit opioid use during study weeks 13 to 24, which was analyzed with an intention-to-treat approach using generalized estimating equations comparing between-group differences.

**Results:**

This sample included 196 participants, predominantly female (119 [60.7%]). Mean (SD) age was 41.0 (10.3) years. Opioid use was 13.4% (95% CI, 6.2%-20.5%) in the M-ROCC group and 12.7% (95% CI, 7.5%-18.0%) in the recovery support group, a 0.6% difference (95% CI, −8.2% to 9.5%; *P* = .89). Cocaine and benzodiazepine use were also not significantly different. Anxiety T scores were reduced across both the M-ROCC and recovery support groups but were not significantly different between groups from baseline to week 24 (1.0; 95% CI, −2.4 to 4.3; *P* = .57). The M-ROCC participants demonstrated a larger reduction in opioid craving compared with the recovery support group participants: −1.0 (95% CI, −1.7 to −0.2; *P* = .01; Cohen *d* = −0.5).

**Conclusions and Relevance:**

In this study, during buprenorphine treatment comparing mindfulness vs active control, both groups significantly reduced anxiety without significant differences in substance use outcomes. Mindfulness led to significantly greater reductions in residual opioid craving than control. The findings of this study suggest that mindfulness training groups may be recommended for people receiving buprenorphine maintenance therapy who have residual opioid craving.

**Trial Registration:**

ClinicalTrials.gov Identifier: NCT04278586

## Key Points

**Question:**

During buprenorphine treatment, does group-based mindfulness training reduce opioid use, craving, and anxiety compared with group recovery support?

**Findings:**

In this randomized clinical trial including 196 adults prescribed buprenorphine for opioid use disorder, mindfulness was not superior at reducing illicit opioid use compared with an active group intervention with an evidence-based curriculum. Both arms experienced significantly reduced anxiety, and the reduction in opioid craving during mindfulness groups was greater than during recovery support groups, a significant difference.

**Meaning:**

The findings of this study suggest that mindfulness groups may have utility during opioid use disorder treatment, especially for patients with residual opioid craving while prescribed buprenorphine.

## Teaser

This randomized clinical trial examines the effect of mindfulness training in adults receiving buprenorphine for opioid use disorder on drug use, anxiety, and craving.

## Introduction

Opioid use is a major public health crisis in the US, with approximately more than 80 000 opioid overdose deaths in 2023.^1^ Buprenorphine treatment reduces illicit opioid use and overdose risk^2,3^; however, studies report that most patients discontinue buprenorphine medical management within 6 months.^4,5^ Several factors that may serve as treatment targets can increase the likelihood of poor outcomes. Comorbid substance use (eg, cocaine, methamphetamine) increases treatment dropout.^6,7^ Psychiatric symptoms (eg, anxiety), benzodiazepine misuse, and opioid craving increase relapse risk.^8,9^ Opioid craving is associated with subsequent use during buprenorphine treatment, is often preceded by negative affect or withdrawal states, and intensifies during exposure to drug cues or stressful life events.^3,6,7,8,9,10,11,12,13^ Behavioral interventions targeting these factors may improve outcomes, but, aside from contingency management, a systematic review identified no clear benefits to adjunctive individual counseling or cognitive-behavioral therapy.^14^ Unlike individual treatment, group treatment attendance has been associated with increased opioid treatment completion, and group-based opioid treatment appears feasible, acceptable, and may improve treatment outcomes.^15^

Mindfulness-based interventions are an increasingly popular evidence-based group treatment for substance use disorders.^16,17^ A recent fully powered randomized clinical trial found that a mindfulness program reduced opioid use and craving among people with both chronic pain and OUD during methadone maintenance.^18^ Mindfulness training appears to increase individuals’ capacities for self-regulation through enhanced attentional control, cognitive control, emotion regulation, and self-related processes.^19^ Mindful behavior change, a curriculum created to leverage those mechanisms, was shown to reduce anxiety symptoms, increase self-regulation, and catalyze health behavior change in trials of the Mindfulness Training for Primary Care program.^20,21^ The established Mindfulness Training for Primary Care curriculum was adapted for patients with OUD and a 24-week trauma-informed Mindful Recovery Opioid Use Disorder Care Continuum (M-ROCC) was created. A single-arm multisite pilot trial found M-ROCC feasible and acceptable during buprenorphine treatment.^22^ Additionally, participants experienced significant reductions in anxiety and decreased benzodiazepine and cocaine use but not opioid use.^23^

The present full-scale clinical trial compared the effectiveness of M-ROCC, delivered as an adjunctive live-online group during buprenorphine treatment, with an attention-balanced nonmindfulness control recovery support group using evidence-based approaches. We hypothesized that M-ROCC would be more effective than a recovery support group at reducing opioid use and anxiety.^24^

## Methods

### Design, Setting, and Recruitment 

We designed this randomized clinical trial, approved by the Cambridge Health Alliance Institutional Review Board, to compare the effectiveness of live-online M-ROCC vs a recovery support group during outpatient buprenorphine treatment. Participants were recruited through social media (ie, Facebook), community partners (eg, Lynn Community Health, Boston Medical Center, North Shore Community Health), online telemedicine health care professionals (eg, Bicycle Health, Boulder Care), and quick response code flyers linking an online referral form, and participants provided informed consent.^25,26^ Participants received financial compensation. Study inclusion required participants to be aged 18 to 70 years with a stable buprenorphine dose prescribed (>4 weeks) for OUD, confirmed by participants signing a consent form for study personnel to contact their health care professional. Because some people receiving buprenorphine attain sustained remission of OUD, this study aimed to enroll individuals with a less clinically stable status, with residual symptoms of anxiety and/or substance use; therefore, participants had either mild or greater anxiety (Patient Reported Outcomes Measurement Information System–Anxiety Short Form 8a [PROMIS-ASF] T score >55) or recent substance use (<90 days of abstinence from alcohol, opioids, benzodiazepines, cocaine, or methamphetamine). Exclusion criteria included psychosis, mania, suicidality or self-injury, cognitive impairment, past mindfulness group experience, expected inpatient hospitalization or incarceration, or group-disruptive behaviors. Research coordinators (including H.G.) screened participants for eligibility through self-report surveys and telephone interviews.^24^ This trial followed the Consolidated Standards of Reporting Trials (CONSORT) reporting guideline. The trial protocol is available in Supplement 1.

### Blinding and Randomization

The data coordinator (J.B.) randomized participants in random blocks of 4, 6, and 8 with a 1:1 ratio, using a random spreadsheet sequence (Excel; Microsoft Corp). The data coordinator concealed allocation in a password-protected file from personnel managing recruitment and screening until the randomization allocation was assigned. Participants and the primary investigator (Z.S.-O.) were blinded to intervention assignments.

### Interventions

Groups were attention matched and offered at the same day and time as their comparator within each cohort. Each group started with a 30-minute informal check-in during which participants completed weekly surveys and research coordinators video-monitored oral toxicology tests in a video communications platform (Zoom; Zoom Video Communication) breakout rooms, recording results with screen capture (Droplr; Droplr Inc).^27^ Then, a 60-minute intervention group was led by 1 to 2 group leaders, including a lead instructor (A.K.F.) and with more than 4 years of group facilitation experience.^24^ Participants without reliable internet access received smartphones with unlimited data plans.

The M-ROCC curriculum had 3 components, starting with a 4-week orientation focused on fostering group engagement through comfort, curiosity, connection, and confidence. Participants continued into a 4-week low-dose mindfulness group, building a trauma-informed foundation for learning mindfulness and increasing daily formal mindfulness practice time. To provide choice about embarking on intensive mindfulness training, we offered those who successfully completed low-dose mindfulness the opportunity to continue into an intensive recovery-focused 16-week mindful behavior change program.^20,21^ This group focused on cultivating mindfulness of the body, breathing, thoughts, and emotions, plus mindful behavior change skills, interpersonal mindfulness practice, increasing self-compassion and emotion regulation, and developing OUD recovery skills, such as mindful savoring and urge surfing.^24^

We designed the recovery support group based on best practices in group-based opioid treatment, using evidence-based techniques while fostering a sense of accountability, shared identity, and supportive community.^15,28,29,30^ It incorporated 8 weeks of group-building orientation followed by 16 weeks of evidence-based treatment techniques for substance use disorders, including cognitive behavioral therapy, motivational interviewing, community reinforcement, and 12-step facilitation.^31,32,33,34,35^

### Measures

All surveys were hosted by Research Electronic Data Capture (REDCap). During the screening and baseline periods, participants completed telephone screening interviews to report demographic characteristics (eg, race and ethnicity) and self-report surveys with substance use and buprenorphine dose information. The interventions in the study organize participants within group cohorts, which feature social elements. These are generally positive for many people, but the experience of group belonging and group cohesion may be influenced by participant experiences of minoritization, implicit bias, and microaggressions, which have been reported to lead to feelings of inclusion and exclusion related to race and ethnicity that might impact attrition or intervention adherence or continuation.^36,37^ In addition, studies have found that demographic variables have been underreported in mindfulness intervention research, leading to systemic bias and inclusion disparities in the field.^38^ Consequently, we report the racial and ethnic makeup of the study participants to contextualize the results and the limitations of generalizability.

### Primary Outcome

Our primary outcome was the number of 2-week periods with both self-reported and biochemically confirmed abstinence from illicit opioid use during study weeks 13 to 24. During each 2-week period, participants completed at least one randomly assigned 14-panel oral toxicologic report via the video communications platform and 2 self-reported weekly surveys inquiring about past 7-day illicit opioid use. Participants were considered abstinent during each of the six 2-week periods if they had no self-reported opioid use and a negative oral toxicology test result for all illicit opioids tested. We hypothesized that participants in the M-ROCC arm would experience more abstinent periods compared with those in the recovery support group.

### Secondary and Exploratory Outcomes

Participants completed the PROMIS-ASF at baseline and weeks 8, 16, and 24. PROMIS-ASF is an 8-item questionnaire using a 5-point scale asking about the past 7 days (1 = never to 5 = always).^39^ The T scores were calculated, with higher scores indicating greater symptoms of anxiety. We hypothesized that participants assigned to M-ROCC would experience greater reductions in anxiety than those in the recovery support group between baseline and week 24.

Secondary outcomes of benzodiazepine and cocaine use were collected for six 2-week periods in the same manner as described for opioids. We hypothesized that M-ROCC participants would experience greater reductions in benzodiazepine and cocaine use than those in the recovery support group.

As a prespecified exploratory outcome, changes in opioid craving during weekly surveys from weeks 1 to 24 were measured. The Opioid Craving Scale asked participants to rate 3 items assessing different aspects of opioid craving on a scale of 0 to 10. Mean ratings were calculated across these items, with higher ratings representing greater opioid craving. In previous research, the Opioid Craving Scale was positively associated with risk for opioid use in the following week.^40^ We hypothesized that participants assigned to M-ROCC would experience greater reductions in opioid craving between baseline and week 24 compared with those in the recovery support group.

### Adverse Events

Staff monitored adverse events at each study visit and via a REDCap survey at weeks 8, 16, and 24, rated by severity, relatedness, and expectedness. Events were reviewed regularly by a National Center for Complementary and Integrative Health–approved data safety and monitoring board.

### Statistical Analysis

Power analyses assumed randomization of 192 individuals, with an effective sample size of 156. This sample size provided 80% power to detect an effect size of 0.45 for negative toxicologic findings for illicit opioids between M-ROCC and the recovery support group, with a 2-sided significance level of *P* < .05, using an unpaired test.

For the primary outcome, we used an intention-to-treat approach to estimate differences between the M-ROCC and recovery support groups in biochemically confirmed illicit opioid abstinence over 6 biweekly time periods during weeks 13 to 24. We used generalized estimating equation logistic regression accounting for clustering at the individual participant level over weeks 13 to 24.

For the secondary outcome of anxiety and the prespecified exploratory outcome of opioid craving, we conducted a difference-in-differences intention-to-treat repeated-measures analysis using linear mixed-effects models with a study week by group interaction term to estimate the relative changes from baseline to week 24. For changes in anxiety, we included only participants with PROMIS-ASF T scores above 55 at baseline.^39^ We used the Benjamini-Hochberg false discovery rate procedure to account for multiple comparisons.^41^ Effect sizes (Cohen *d*) were calculated.

We used maximum likelihood estimation to address missingness for all analyses, adjusting the models to account for baseline covariates that differed between study groups after randomization (*P* < .10). We conducted a supplemental analysis using multiple imputation. We also conducted supplemental sensitivity analyses adjusting for all covariates associated with the outcome measure missingness. We conducted completer analyses for all outcomes among a subsample of intervention-adherent participants, defined as completing at least 15 of 24 sessions. For the number of adverse events, we conducted a negative binomial regression to evaluate between-group differences. All analyses were conducted in Stata, version 18 (StataCorp LLC).

## Results

### Participant Characteristics

**Figure 1.**
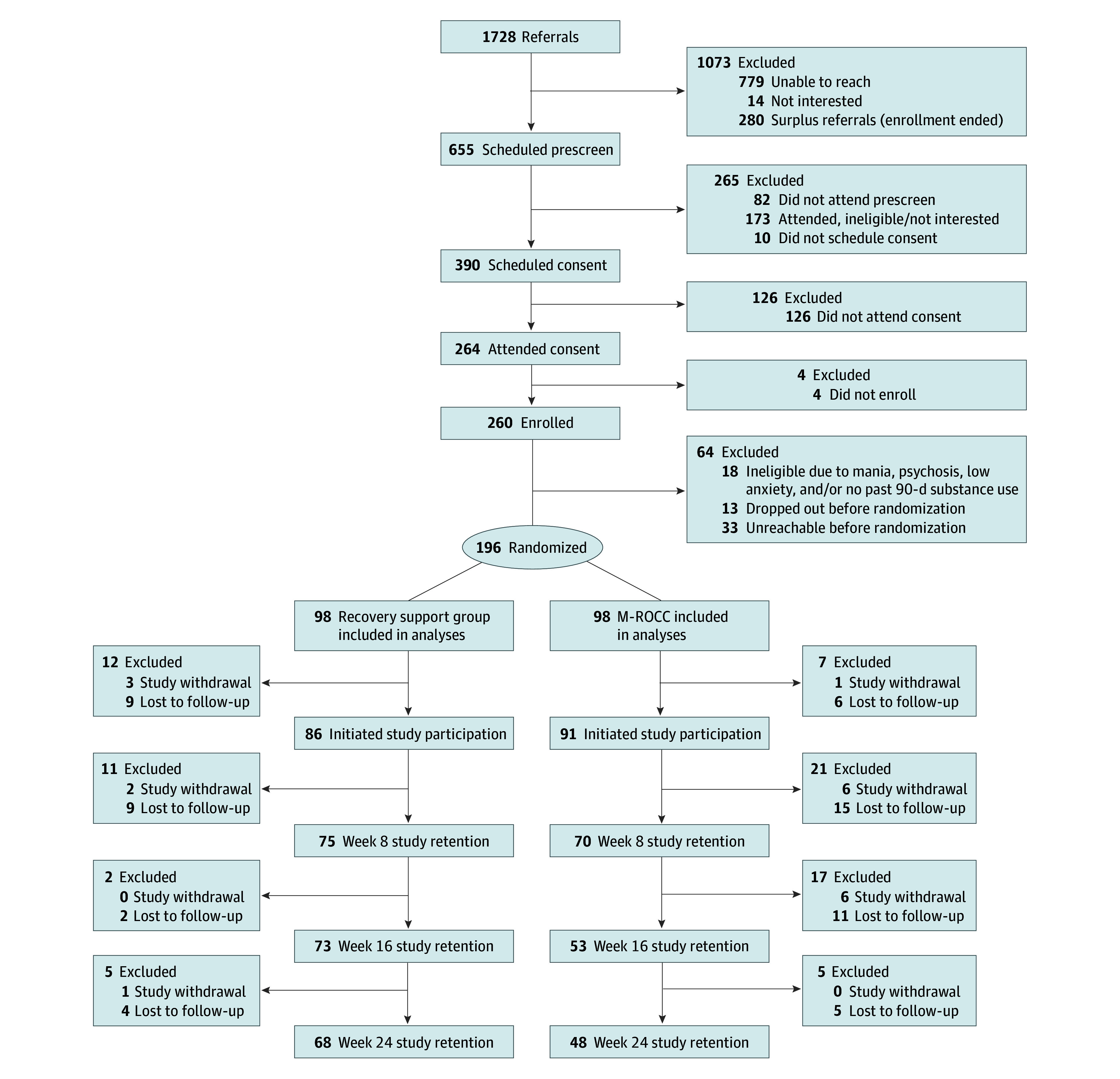
Patient Flowchart M-ROCC indicates Mindful Recovery Opioid Use Disorder Care Continuum.

Of 1728 patients referred between January 21, 2021, and February 15, 2023, 260 participants signed informed consent forms. We excluded 64 individuals for exclusion criteria (n = 18) or incomplete baseline assessments (n = 46) and randomized 196 participants to M-ROCC (n = 98) or the recovery support group (n = 98) (Figure 1). Of these individuals, 119 were female (60.7%), 75 were male (38.3%), and 1 (0.5%) was nonbinary. Mean (SD) age was 41.0 (10.3) years. Once 192 participants were randomized, recruitment ended, although 4 screened participants were able to complete the consent process and join the final cohort. Data collection was completed September 19, 2023. Baseline buprenorphine dose, cocaine use, and annual income differed between groups and were added to the models for primary, secondary, and exploratory outcomes (Table 1).

**Table 1.** Baseline Demographic and Clinical Characteristics of Participants by Study Arm

Characteristic	Study arm, No. (%)		
	M-ROCC (n = 98)	Recovery support group (n = 98)	Total (N = 196)
Gender			
Female	60 (61.2)	59 (60.2)	119 (60.7)
Male	38 (38.8)	37 (37.8)	75 (38.3)
Nonbinary	0	1 (1.0)	1 (0.5)
Unknown	0	1 (1.0)	1 (0.5)
Age, mean (SD)	41.2 (10.4)	40.9 (10.3)	41.0 (10.3)
Race^a^			
White	90 (91.8)	90 (91.8)	180 (91.8)
Ethnicity			
Hispanic, Spanish, or Latinx	10 (10.2)	6 (6.1)	16 (8.2)
Marital status			
Single	43 (43.9)	44 (44.9)	87 (44.4)
Married or living as married	29 (29.6)	37 (37.8)	66 (33.7)
Divorced	18 (18.4)	14 (14.3)	32 (16.3)
Widowed	6 (6.1)	3 (3.1)	9 (4.6)
Educational level, mean (SD), y^b^	13.1 (2.2)	13.5 (2.6)	13.3 (2.4)
Sexual identity			
Straight or heterosexual	81 (82.7)	78 (79.6)	159 (81.1)
Lesbian, gay, or homosexual	3 (3.1)	6 (6.1)	9 (4.6)
Bisexual	9 (9.2)	10 (10.2)	19 (9.7)
Other	5 (5.1)	4 (4.1)	9 (4.6)
Annual income, $			
<20 000	37 (37.8)	46 (46.9)	83 (42.3)
20-80 000	53 (54.1)	38 (38.8)	91 (46.4)
>80 000	7 (7.1)	14 (14.3)	21 (10.7)
Buprenorphine dose, mean (SD)	13.3 (6.3)	15.7 (6.9)	14.5 (6.7)
Opioid Craving Scale score, mean (SD)	3.4 (2.5)	3.0 (2.5)	3.2 (2.5)
Unemployment status	18 (18.4)	17 (17.4)	35 (17.9)
Region			
Northeast	24 (24.5)	27 (27.6)	51 (26.0)
South	48 (49.0)	47 (48.0)	95 (48.5)
Midwest	14 (14.3)	10 (10.2)	24 (12.2)
West	12 (12.2)	14 (14.3)	26 (13.3)
≥4 ACEs	65 (66.3)	65 (66.3)	130 (66.3)
PROMIS-Anxiety T score, mean (SD)^c^	64.5 (6.2)	65.9 (6.8)	65.2 (6.5)
PROMIS-Pain Interference T score, mean (SD)	60.9 (9.0)	59.1 (10.1)	60.0 (9.6)
Used medicines or drugs	97 (99.0)	96 (98.0)	193 (98.5)
Sedatives or tranquilizers	89 (90.8)	83 (84.7)	172 (87.8)
Painkillers	97 (99.0)	95 (96.9)	192 (98.0)
Cocaine or crack	78 (79.6)	67 (68.4)	145 (74.0)
Heroin	56 (57.1)	59 (60.2)	115 (58.7)
Used fentanyl within 90 d before screening^d^	8 (8.4)	3 (3.3)	11 (5.9)
Scheduling acceptability, mean (SD)^e^	8.1 (2.6)	8.2 (2.2)	8.1 (2.4)
Past mindfulness intervention experience	17 (17.3)	21 (21.4)	38 (19.4)

Abbreviations: ACE, adverse childhood experience; M-ROCC, Mindful Recovery Opioid Use Disorder Care Continuum; PROMIS, Patient Reported Outcomes Measurement Information System–Anxiety Short Form 8a.

^a^ For the M-ROCC study arm, the population not in the category of White race included American Indian (1), Black or African American (1), Native Hawaiian (1), more than one race (2), and unknown (3). For the recovery support group, the population not in the category of White race included Asian (1), unknown (4), and more than one race (3).

^b^ Two participants in the M-ROCC group did not report years of education.

^c^ Of 196 participants, only 7 (3.6%) had T scores less than 55 for PROMIS-Anxiety.

^d^ Question answered by 95 individuals in the M-ROCC group and by 91 in the recovery support group.

^e^ Question answered by 83 individuals in the M-ROCC group and by 84 in the recovery support group.

### Outcomes

During weeks 13 to 24, mean illicit opioid nonabstinence time periods were 13.4% (95% CI, 6.2%-20.5%) in the M-ROCC group and 12.7% (95% CI, 7.5%-18.0%) in the recovery support group, a difference that was not statistically significant (0.6%; 95% CI, −8.2% to 9.5%; *P* = .89) (Table 2). During weeks 13 to 24, benzodiazepine use time periods did not differ significantly between the M-ROCC (22.1%) and recovery support (20.2%) groups (1.9%; 95% CI, −10.3%- 14.1%; *P* = .76) (Table 2). Similarly, there was no significant difference in cocaine use periods between the M-ROCC (8.4%) and recovery support (1.5%) groups (6.9%; 95% CI, −2.4%-16.2%; *P* = .15).

**Table 2.** Abstinence Outcomes by Arm

Measure	Weeks 13-24		
	Nonabstinent periods, % (95% CI)	Difference for M-ROCC vs recovery support	
		% (95% CI)	*P* value
**Primary measure**			
Nonabstinent periods of opioids			
Recovery support	12.7 (7.5 to 18.0)	0.6 (−8.2 to 9.5)	.89
M-ROCC	13.4 (6.2 to 20.5)		
**Secondary measure**			
Nonabstinent periods of cocaine			
Recovery support	1.5 (0 to 4.0)	6.9 (−2.4 to 16.2)	.15
M-ROCC	8.4 (0 to 16.6)		
Nonabstinent periods of benzodiazepines			
Recovery support	20.2 (12.7 to 27.7)	1.9 (−10.3 to 14.1)	.76
M-ROCC	22.1 (12.7 to 31.5)		

Abbreviation: M-ROCC, Mindful Recovery Opioid Use Disorder Care Continuum.

Large effect size reductions in anxiety from baseline to week 24 were observed in the recovery support group, with a mean T score change of −10.0 (95% CI, −12.0 to −8.0; *P* < .001; Cohen *d* = −1.3), and in the M-ROCC group, with a mean T score change of −9.0 (95% CI, −11.7 to −6.3; *P* < .001; Cohen *d* = −1.1). The interaction term for study group by week (weeks 0, 8, 16, and 24) was not significant (χ^2^_3_ = 4.5; *P* = .31), and there was no significant difference between study groups at week 24 (95% CI, 1.0; −2.4 to 4.3; *P* = .57) (eFigure 1 in Supplement 2).

**Figure 2.**
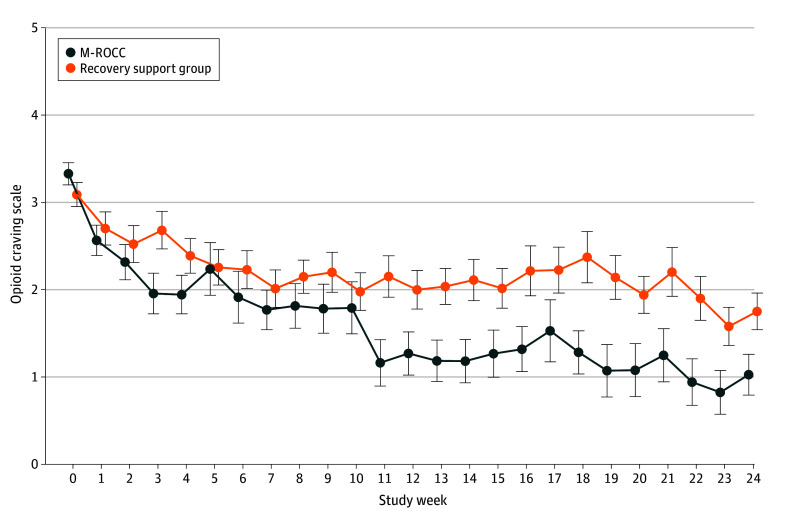
Opioid Craving Scale Total by Study Week and Group Marginal estimates of the Opioid Craving Scale score. Examining variation in opioid craving over time by study group, the interaction term for study group by study week, when added to the model in addition to the main effects, was significant (χ^2^_24_ = 56.5; *P* < .001). Vertical bars indicate SE of the estimate. M-ROCC indicates Mindful Recovery Opioid Use Disorder Care Continuum.

In exploratory analysis of change in opioid craving over time, we added baseline opioid craving to the other outcome covariates. The interaction term for study group by week was significant (χ^2^_24_ = 56.5; *P* < .001). At week 24, the recovery support group mean opioid craving decreased by −44% (−1.3; 95% CI, −1.9 to −0.8; *P* < .001; Cohen *d* = −0.7) compared with a −67% (−2.3; 95% CI, −2.9 to −1.7; *P* < .001; Cohen *d* = −1.3) decrease in the M-ROCC group (Table 3). This represented a significant differential reduction among the M-ROCC group compared with the recovery support group (−1.0; 95% CI, −1.7 to −0.2; *P* = .01; Cohen *d* = −0.5) (Figure 2).

**Table 3.** Secondary and Exploratory Outcomes by Group

Measure	Study group by week interaction		Week 24 to 0		Difference in week 24 to week 0 for M-ROCC vs recovery support	
	χ^2^ (*df*)	Adjusted *P* value^a^	Difference (95% CI)	Effect size	Difference (95% CI)	Effect size
**Secondary**						
PROMIS-Anxiety						
Recovery support	4.5 (3)	.31	−10.0 (−12.0 to −8.0)	−1.3	1.0 (−2.4 to 4.3)	0.1
M-ROCC			−9.0 (−11.7 to −6.3)	−1.1		
**Exploratory (prespecified)**						
Opioid Craving Scale score						
Recovery support	56.5 (24)	<.01	−1.3 (−1.9 to −0.8)	−0.7	−1.0 (−1.7 to −0.2)	−0.5
M-ROCC			−2.3 (−2.9 to −1.7)	−1.3		

Abbreviations: M-ROCC, Mindful Recovery Opioid Use Disorder Care Continuum; PROMIS, Patient Reported Outcomes Measurement Information System.

^a^ The *P* values for the Study Group by Week Interaction were adjusted using the Benjamini-Hochberg false discovery rate procedure to account for multiple comparisons across this family of 2 outcome measures according to the Cao et al method.^41^

Results of the imputation analyses for primary, secondary, and exploratory analyses did not differ substantially from the maximum likelihood estimation analyses (eTable 1, eTable 2, and eFigure 2 in Supplement 2). Sensitivity analyses using all covariates associated with missingness (eg, COVID-19 Delta and Omicron wave cohorts) on the primary, secondary, and exploratory outcomes also had similar results (eResults 1, eTable 3, and eTable 4 in Supplement 2). Only 59% of the participants (116 of 196) completed week 24 of the study. Completer analyses also had similar results. A completer analysis found that women (52.9%) were more likely than men (41.3%) to continue after week 8 in both arms, and non-Hispanic White individuals who spoke English (48.8%) were more likely than others (6.3%) to continue into the intensive M-ROCC after week 8.

### Adverse Events 

There were no significant between-group differences in adverse events. One adverse event, which was of mild severity, was intervention-related (ie, pain during mindful movement practice in the M-ROCC group) (eResults 2 in Supplement 2).

## Discussion

This geographically diverse randomized clinical trial recruiting from 16 states (eFigure 3 in Supplement 2) demonstrated that M-ROCC was not more effective than a nonmindfulness, evidence-based recovery support for reducing illicit opioid, benzodiazepine, or cocaine use. Infrequent opioid use in both groups may have limited the study’s power to detect between-group differences. This may have resulted from positive intervention effects, study attrition, missing data, or selecting a sample of participants receiving stable buprenorphine doses for at least 30 days. Additionally, both the M-ROCC and recovery support groups demonstrated similarly large reductions in anxiety, suggesting that, irrespective of theoretical approach, group-based live-online psychosocial interventions may have similar benefits for anxiety during buprenorphine treatment.

The M-ROCC participants experienced a differential reduction in opioid craving, a risk factor for illicit opioid use and treatment dropout during buprenorphine treatment.^40,42,43^ Similar craving reductions were observed in a recent study of mindfulness among opioid misusers with chronic pain.^44^ However, unlike this and other prior research,^45^ differential craving reductions among M-ROCC participants did not translate into significantly less opioid use than observed in the comparator intervention group. Participants were required to have stable buprenorphine doses for 30 days or more, which resulted in relatively low levels of baseline residual craving and possibly less opioid use.

Several mechanisms may explain the differential reduction in opioid craving among M-ROCC participants.^46,47^ Mindfulness-based interventions may ameliorate reward processing dysfunction through mindful savoring practices designed to resensitize people with OUD to natural reward signals.^48,49^ Craving involves interoceptive processing, and several mindfulness practices (eg, body scan) may impact craving by enhancing healthy interoceptive awareness and correcting interoceptive dysregulation.^50,51,52,53,54,55,56^ Mindfulness enhances self-regulation capacity and improves emotion regulation, thereby reducing reactivity to negative affect and breaking associations between negative affect and substance use craving.^19,21,57,58^ Additionally, mindfulness training reduces attentional bias toward opioid-related cues, possibly reducing autonomic reactivity and enhancing cognitive control during a craving response.^59,60,61^ Mindful urge surfing represents a resilient coping response, reducing craving elaboration and increasing awareness of early signs of craving.^62,63^ Repeated urge surfing with successful inhibition of craving-related responses paired with reconnection to deeply held values may uncouple activating drug-use cues from conditioned appetitive responses^64,65^ and realign motivation, helping sustain behavior change.^19,66,67^

Group-based opioid treatment is an increasingly common approach to providing concurrent behavioral health interventions during buprenorphine treatment.^15,28,29,30,68^ Groups may facilitate improved treatment outcomes by teaching coping techniques and increasing social support, which has been associated with decreased substance use and improved retention in medications for opioid use disorder treatments.^69^ More research comparing group-based opioid treatment directly with individual care is needed, as well as understanding which implementation factors (eg, telehealth/in-person, delivery of evidence-based curriculum, and providing buprenorphine prescriptions during group) may support improved outcomes in group-based opioid treatment.^28,30^ The use of a group-based opioid treatment control arm incorporating evidence-based interventions for substance use disorder distinguishes this study from another recent randomized clinical trial^18^ for people with chronic pain during methadone maintenance that compared an adjunctive telehealth mindfulness group with an active supportive psychotherapy group control that did not provide any therapeutic skill training. In that study, the mindfulness arm demonstrated fewer drug use days and greater medication adherence, although anxiety was not significantly different between the groups.

The results of this present study align with meta-analyses suggesting that mindfulness, while often better than passive controls, does not differ substantially from other evidence-based interventions with respect to substance use and anxiety outcomes.^70,71^ In contrast, meta-analyses suggest that mindfulness outperforms active controls for reducing cravings among individuals with substance use disorders.^72,73^ This trial extends these findings, highlighting that mindfulness training may be helpful for patients with residual craving during buprenorphine treatment. The findings of this trial suggest the utility of mindfulness training as an evidence-based adjunctive approach for treating residual craving during opioid treatment with buprenorphine.

### Limitations

This study has limitations. Higher levels of attrition in the M-ROCC group were noted compared with the pilot study,^23^ especially between weeks 8 and 16, when the intensive mindfulness program started. To be trauma informed, M-ROCC leaders encouraged participants at week 8 to consider their personal motivations for continuing into the more intensive Mindfulness Training for Primary Care OUD curriculum, emphasizing the choice to continue or withdraw from the group. The recovery support group did not have similar warnings about changing intervention intensity. Studies of trigger warnings suggest they do not typically lead to therapeutic avoidance in the general population^74^; however, levels of experiential avoidance can be higher among patients with OUD.^75^ Women were more likely than men to continue in both arms, and non-Hispanic White individuals who spoke English were most likely to continue into the intensive M-ROCC, suggesting that these warnings might have been experienced differently based on gender, identity, and culture. Additionally, the significant difference between groups in opioid craving changes over time could have resulted from a smaller, more committed group of engaged individuals continuing in M-ROCC compared with recovery support. Future multivariate analyses will be conducted to examine the effects of differential attrition on craving outcomes.

Stress, illness, and changes in lifestyle or employment changes due to the COVID-19 pandemic created barriers for multiple participants to engage with this study, resulting in higher than expected attrition particularly during cohorts overlapping with the Delta and Omicron waves of COVID-19 infections. Nevertheless, intention-to-treat analysis using maximum likelihood estimation methods allowed all 196 participants to be included in the final analyses.

The study’s predominantly White sample reflects national statistics on buprenorphine treatment engagement, but the study enrolled fewer Black participants than expected, allowing the possibility that findings may not generalize to all populations. Geographic and regional diversity was a unique strength of this study (eFigure 3 in Supplement 2), but integration of geographically diverse populations with different racial and ethnic and cultural backgrounds into common live-online groups added complexity during an intense period of national racial unrest that started in 2020.^76,77,78^ This study also lacked a control condition with no behavioral treatment; therefore, it is unclear whether specific behavioral interventions, general group effects, or time in buprenorphine treatment were the primary factors of anxiety reduction.

## Conclusions

In this randomized clinical trial, the impacts of a trauma-informed mindfulness-based group intervention during buprenorphine treatment on opioid use, substance use, and anxiety were similar to a recovery support group with a curriculum using evidence-based substance use treatment approaches. While further research is required, the study suggests that mindfulness-based groups may be particularly useful for reducing craving among patients with OUD who are experiencing residual opioid craving during buprenorphine treatment.

## Notes

Supplement 1. Study Protocol

Supplement 2. **eFigure 1.** PROMIS-Anxiety by Study Week and Group **eTable 1.** Abstinence Outcomes by Arm With Additional Covariates Using Imputation **eTable 2.** Secondary and Exploratory Outcomes by Group With Additional Covariates in Sensitivity Analysis Using Imputation **eFigure 2.** Opioid Craving Scale Total by Study Week and Group With Imputation **eResults 1.** Sensitivity Analysis **eTable 3.** Abstinence Outcomes by Arm in Sensitivity Analysis With Additional Covariates **eTable 4.** Secondary and Exploratory Outcomes by Group With Additional Covariates in Sensitivity Analysis **eResults 2.** Adverse Events **eFigure 3.** Geographic Distribution of Randomized Participants

Supplement 3.
Data Sharing Statement
