# Supplementary material for: Mindfulness Training vs Recovery Support for Opioid Use, Craving, and Anxiety During Buprenorphine Treatment: A Randomized Clinical Trial
Source: JAMA Netw Open. 2025 Jan 21;8(1):e2454950. doi: 10.1001/jamanetworkopen.2024.54950 (PMC11751747; doi:10.1001/jamanetworkopen.2024.54950)
Supplement: Supplement 1. — Study Protocol [file jamanetwopen-e2454950-s001.pdf]

Effect of Mindfulness Training on Opioid Use and Anxiety During Primary Care Buprenorphine Treatment (MINDFUL-OBOT)

R33 AT10125

Phase 1: Primary Care RCT
Phase 2: Live-Online RCT

PRINCIPAL INVESTIGATOR

Zev Schuman-Olivier, MD

1.

TOOL REVISION HISTORY

Version Number: 1
Version Date: 06/19/19
Summary of Revisions Made:
Version 1: Initial Submission

Version Number: 1.1
Version Date (Resubmitted at 3 points): 10/7/19, 10/31/19, 11/8/19
Summary of Revisions Made:
Upon the request of the IRB the following stipulations have been made to the protocol:

1. Protocol version and version date updated
2. Pagination corrected to 99 total pages
3. Added Nancie Rouleau and Roger Weiss to the protocol
4. Update Roger Weiss as a non-CHA affiliated team member
5. MTPC-OUD enrollment corrected and justified based on Mindful-OBOT R-21 pilot
6. Time of treatment discontinuation revised and updated with correct information
7. Length of LDM and MCS sessions clarified in Figure 3
8. Inclusion criteria in regards to the definition of 90 days of abstinence refined
9. Clarified when participants enter MCS, and when they stay in LDM
10. Rationalized option for M-ROCC participants to switch back into GBOT
11. Weekly/Biweekly Group timing clarified
12. Alternatives to participation clarified
13. Information on social media privacy and security added
14. Consenting procedure section updated to include CHA and non-CHA sites
15. Procedure for assigning study numbers and acrostics clarified
16. Timing of randomization updated
17. Included company information of prepaid cards, and what data will be shared with CT Payer
18. Risks of participants disclosing private information added
19. Included timeframe for reporting unanticipated problems that are not adverse
20. Revised protocol for Heart Rate Detection task to indicate no intervention in the event of abnormal heartrate detection
21. Description of exported file of site-specific medical record information updated
22. Removed reference to paper and pen surveys
23. Reference to disguising information updated to reflect procedure for creating study numbers and acrostics
24. Clarified study payments.
25. Removed all references to qualitative interviews

26. Removed reference to obtaining a waiver of consent
27. Removed grammatical error in section 7.2.1
28. Clarified statement on group acceptability survey matching
29. Clarified Sarah Moore's institution

Version Number: 1.2 (Initial Submission to NCCIH for Approval)
Version Date:10/22/19
Summary of Revisions Made:

Version 1.2:

1. Removed Dharma Cortes from the study team roster
2. Removed Genie Bailey from the study team roster
3. Removed Yanyi Jiang from study team roster
4. Removed Cassandra Harding from the study team roster
5. Added Farah Samawi to the study team roster
6. Added Timothy Creedon to the study team roster
7. Removed Jen Brownstein from the study team roster
8. Reduced study enrollment from 280 to 180
9. Reduced study length from 52 weeks to 24 weeks and changed 6 month references to 24 weeks for consistency
10. Changed study title from "Effects of Mindfulness Training on Primary Care Buprenorphine Treatment Retention" to "Effect of Mindfulness Training on Opioid Use and Anxiety During Primary Care Buprenorphine Treatment"
11. Edited font size to be consistent throughout document
12. Corrected spelling of Alexandra Comeau's name
13. Removed reference to R01 grant
14. Removed SSTAR as a site
15. Removed North Shore Peabody site
16. Replaced references to 8 sites across 5 institutions with five primary care sites and clarified figures to reflect this change.
17. Added 16-week version of MTPC-OUD intensive
18. Added Senior Research Coordinator to Data Quality monitoring table
19. Changed primary outcomes to R33 outcomes instead of R01 outcomes, focusing on change in PROMIS anxiety.
20. Clarified Richa Gawande's role
21. Clarified that manual will be sent to NCCIH prior to the start of R33 study enrollment
22. Clarified Serious Adverse Event as "life-threatening" overdose requiring naloxone reversal, rather than just "overdose".
23. Clarified Data Quality Monitoring table reviewer roles
24. Corrected grammatical error in section 7.2.1
25. Added Beck Anxiety Inventory (BAI) as a secondary outcome

26. Added PROMIS Anxiety T score less than 50 (raw < 13) as an exclusion criteria and modified screening procedure to reflect this.

Version Number: 1.3 (Re-Submission to NCCIH and CHA IRB for Approval)
Version Date1/6/2020: Modification 1
Summary of Revisions Made:

1. PROMIS Anxiety T score less than 55 (raw < 16) will be excluded from the secondary analysis of PROMIS Anxiety and BAI outcomes, because 55 is the cutoff. for mild anxiety. This analysis will be conducted in subset with anxiety disorders.
2. Changed primary and secondary outcomes to the outcomes from original R33 grant with opioid negative time periods as a primary outcome.
3. Changed sample size from 180 enrolled to 210 enrolled, and included updated power analysis for this sample.
4. Removed PROMIS Anxiety T score less than 50 (Raw<13) as an inclusion criterion.
5. Clarified procedures for obtaining waivers of consent.
6. Defined MOCA as Montreal Cognitive Assessment.
7. Changed payment method from CT payer to PNC bank, and specified which data will be collected by PNC bank.
8. Clarified Richa Gawande's role in study meetings.
9. Added Kayley Okst as a research coordinator.
10. Added language about matching acceptability of GBOT and LDM study group times when there are multiple GBOT groups to consider at a site.
11. Increased transportation reimbursement amount from $25 total to $10 for each study session.
12. Clarified process for data transfer from EHR to REDCap.
13. Changed 6 month references to 24 weeks for consistency.
14. Included section on measuring and reporting of participant adherence to treatment protocol.
15. Removed reference to Myspace in sending private messages.
16. Clarified worsening of underlying illness section.
17. Clarified when data analyst and study methodologists will become unblinded.
18. Corrected fishbowl ratios to be in line with evidence-based protocol by Petry, et al.
19. Added the Nonattachment to Self Scale and adjusted survey length accordingly.
20. Clarified use of False Discovery Rate procedures for addressing multiple comparisons among secondary and exploratory outcomes.
21. Clarified use of PROMIS-ASF 8a T score greater than 55 to determine presence of an anxiety disorder in participants during screening and for inclusion in secondary analysis for effects on anxiety.

Version Number: 1.4 (Second Re-Submission to NCCIH for Approval)

Version Date:1/29/2020 (Modification 1 second resubmission to CHA IRB)
Summary of Revisions Made:

1. Included statement that clinical support staff and other patients in clinical groups that participants are randomized into will not need to be added to the study protocol in a comparative effectiveness trial.
2. Clarified that the study team will obtain waivers of consent at sites.
3. Clarified word missing in statement on group acceptability survey matching.
4. Formatted GBOT framework figure and made it landscape for readability.
5. Included that life-threatening suicide attempts and life-threatening opioid overdoses requiring naloxone administration will be considered Serious Adverse Events, and non-life-threatening suicide attempts are only unexpected if person has no history of suicidality or risk factors.
6. Clarified the SAE definition to be aligned with CHA policy on AE reporting. Clarified the requirement for life threatening overdose (defined by requiring naloxone) and life-threatening suicide attempts (defined by requiring ED visit or inpatient hospitalization) to be SAEs. Clarified that inpatient admission strictly due to substance use disorder alone is not SAE. Added additional categories for CHA policy (e.g. birth defects, etc.).
7. Clarified reporting time frame for SAE and UAs to be consistent with CHA policy on AE reporting.
8. Clarified power statement.
9. Fixed missing reviewer in monitoring table for major protocol violations and corrected capitalization error.

Version Number: 1.5
Version Date: 3/17/2020 (Modification 1 third resubmission to CHA IRB)
Summary of Revisions Made:

1. Moved information regarding non-study participant involvement in study groups to section 5.1.
2. Corrected grammatical errors in interventions and duration section.
3. Corrected range of standard of care for opioid treatment from 24 weeks to 3-12 months.
4. Corrected planned number of enrollments at each study site.
5. Removed reference to 6-month study in secondary objective section.
6. Stated that the document used to collect PHI and enter this information into the PNC bank secure server will be shredded after it the information is entered.
7. Provided gift card alternative to participants who are unwilling to provide PHI to PNC bank.
8. Made references to PROMIS Anxiety (PROMIS-ASF) scale consistent and clear throughout protocol.

9. Made references to PROMIS Pain (PROMIS-PISF) scale consistent and clear throughout protocol.
10. Clarified references to 52-week follow up throughout protocol.
11. Clarified that weekly surveys will be sent via email with secure REDCap link, and that these surveys will be completed using a CHA IT approved iPad if this is not possible.
12. Included the Substance Craving Scale (SUBCS)
13. Included the Pain Catastrophizing Scale (PCS)

Version Number: 1.6
Version Date: 4/14/20 (Modification 1 fourth resubmission to CHA IRB)
Summary of Revisions Made:

1. Corrected wording error in "standard clinical staff in comparative effectiveness study" section.
2. Clarified at which sites GBOT is the standard of care, and which sites are running standard of care GBOT groups.
3. Expanded inclusion criteria to allow enrollment of participants with a diagnosis of OUD and an anxiety or stress disorder who do not meet the less than 90 days of abstinence criterion.
4. Specified that initial EHR screening will occur after participant is referred to the study.

Version Number: 2.0
Version Date: 9/3/20 (Modification 2 submitted to NIH)
Summary of Revisions Made:

1. Removed all references to in person groups and replaced with references to live-online groups.
2. Specified that while participants will be recruited virtually from multiple different sites, the study will now be conducted centrally at the CHA Center for Mindfulness and Compassion.
3. Changed primary outcome to focus on focus on abstinence from illicit opioids.
4. Changed main secondary outcomes to focus on change in co-morbid anxiety, pain, cocaine use, and benzodiazepine use.
5. Removed GBOT arm and replaced it with a standard control group arm.
6. Added qualitative interview as an exploratory outcome.
7. Clarified that groups will not be billable to insurance and will instead be run as research groups.
8. Indicated that group leadership manuals will be adapted to a suit a live-online setting.
9. Replaced all references to urine screens with live-online supervised oral fluid screens.
10. Added Computerized Adaptive Testing for Mental Health (CAT-MH) for psychiatric comorbidity as an exploratory outcome and replaced the MINI with baseline CAT-MH for screening.

11. Removed SART, HDT, and HBDT from survey battery.
12. Changed survey battery timepoints to occur at baseline, 8 weeks, 16 weeks, and 24 weeks and updated survey references to reflect this change.
13. Indicated that participants will undergo a pre-enrollment phone screen to determine eligibility if they consent to do so.
14. Removed requirement that participant referral take place over EHR.
15. Changed randomization ratio to 1:1 (M-ROCC:Control)
16. Removed language about comparative effectiveness and inclusion of standard clinical staff in groups since study will change to clinical trial examining efficacy of live-online M-ROCC versus a control group.
17. Added the Self-Critical Rumination Scale and the Experiences Questionnaire subscale for Decentering
18. Removed all references to 52 week follow-up from protocol.
19. Removed PROMIS Depression and substituted it with CAT-DI
20. Removed references to mediation analyses
21. Removed cannabis use as an inclusion criteria

Version Number: 2.1
Version Date: 9/1/20
Summary of Revisions Made:
1. Corrected grammar and spelling errors throughout document.
2. Specified use of practice dose ladder in M-ROCC intervention.
3. Provided further details regarding live-online control intervention.
4. Edited figure 1 to reflect updated recruitment approach.
5. Clarified group session frequency during first 4 weeks of intervention.
6. Inserted information on handling of missing assessment data.
7. Replaced PNC bank as the preferred gift card vendor with Tango and removed references to SSN collection.
8. Changed anticipated loss during screening and baseline assessments.
9. Specified that oral-fluid swab chamber will be placed in clear view of the research. coordinator during the testing period.
10. Replaced DERS-36 with DERS-16 to reduce participant burden.
11. Removed reference to obtaining waiver of consent
12. Revised primary outcomes to encompass biochemically confirmed opioid abstinent periods using both oral fluid tests and self-report

Version Number: 2.2
Version Date: 9/24/20
Summary of Revisions Made:
1. Removed references to SMART IRB, reliance agreements, and site PIs throughout protocol.

2. Defined MBI in precis section before using acronym.
3. Reinserted relevant descriptions of intervention, duration, sample size and population which were previously deleted in error.
4. Corrected number of interventions in study rationale section.
5. Defined relationship between RNCMs and BMC.
6. Standardized recruitment process across protocol, by which referring clinicians and nurse care managers will obtain verbal consent to share information with the study team, and not engage in any study related activities.
7. Clarified use of scheduling acceptability form.
8. Specified process for destroying phone screen data for participants who do not enroll in the study.
9. Clarified that participants with transient symptoms related to recent substance use will be able to wait for a re-review of eligibility after 30 days.
10. Moved M-ROCC group outline figure to interventions section.
11. Standardized MCS group administration, whereby MCS groups will be held online via zoom as live online community groups for addiction recovery and mindfulness maintenance, and not at individual sites.
12. Moved description of M-ROCC development to background section.
13. Defined where ascending practice dose ladder is implemented.
14. Defined difference between LDM and MTPC-OUD groups.
15. Clarified that participants will be able to advance from the LDM group to either an MCS or MTPC-OUD group.
16. Moved qualitative interview description to study procedures section.
17. Indicated that an electronic consent module will be used to document consent in REDCap.
18. Described qualitative interview download and transcription method.
19. Clarified modules used in CAT-MH screening tool and that CAT-MH will be integrated into REDCap.
20. Specified under which circumstances oral fluid screen results will be shared with a patient’s referring provider.
21. Specified number of referring primary care sites across MA and use of Facebook for recruitment as well from primary care site referrals.
22. Included information on what data that will be shared with referring providers and specified that groups will not replace standard of care at each site.
23. Specified that participants will receive gift cards via email when using tango.
24. Required study staff to use secure technology to communicate with participants.
25. Updated lottery draw contingency management procedure and specified when participants will make lottery draws.
26. Clarified that participant EHR will not be accessed.
27. Removed reference to use of lockboxes to transport paper data.

28. Specified payment amount for qualitative interview completion.
29. Indicated that the study flyer will be posted on the CHA addictions website.
30. Removed statement that suicide risk detected by CAT-MH will automatically trigger an email to the study team.
31. Specified information that will be collected on study inquiry contact form (phone screen)

Version Number: 2.3
Version Date: 10/27/20
Summary of Revisions Made:

1. Expanded abbreviation of Training and Technical Assistance (TTA).
2. Removed references to Facebook advertisement.
3. Included structure of live-online CMC MCS groups.
4. Removed remaining references to site research coordinators.
5. Removed references to CAT-MH data being stored outside of REDCap.
6. Renamed "double-locked lockboxes and filing cabinets" section as "online database storage".
7. Specified that participants will receive iPhone SEs via mail after they have been randomized to join the study.
8. Clarified that live-online control group will run for 80-90 minutes including a 30-minute group check-in.
9. Rephrased study design to make clear that participants from different primary care sites will come together for study groups.
10. Fixed grammatical errors throughout protocol.
11. Made references to 3 non-CHA affiliated and 2 CHA affiliated OBOT sites consistent throughout protocol.
12. Corrected team study roster to list individual's institution, rather than their institution of primary employment.
13. Removed non-CHA affiliated staff who will be added in future SMART IRB amendment.
14. Clarified that the three affiliated non-CHA sites (BMC, North Shore, Lynn) will not engage in study activities until their corresponding reliance agreement has been executed.
15. Made references to primary and secondary outcomes consistent across protocol.
16. Corrected conditions for participant completion bonus to be consistent across protocol and ICF.

Version Number 2.4
Version Date: 11/20/20
Summary of Revisions Made:

1. Corrected grammatical errors throughout document.
2. Specified that R21 pilot data indicated reduction in positive Benzodiazepine and Cocaine urine screen tests.

3. Restated that if the participant fails a second informed consent quiz, they will be ineligible to participate in the study.

Version Number 2.5
Version Date: 12/20/20
Summary of Revisions Made:

1. Added Gabriella Conversano to the protocol as a research assistant.
2. Added Bari-Sue Brodsky to the protocol as an M-ROCC group leader.
3. Added Ashley Mallon to the protocol as an M-ROCC group leader.
4. Added Emily Tavanese to the protocol as an M-ROCC group leader.
5. Added Joseph Rosansky to the protocol as a research assistant.
6. Added Megan Edge to the protocol as a research assistant.
7. Added Caitlyn Wilson to the protocol as a research assistant.
8. Added Audrey Cabral to the protocol as a research assistant.
9. Added Connor Stuart to the protocol as a control group leader.
10. Added Esteban da Cruz to the protocol as a research assistant.
11. Removed Brian Mullin from the protocol.
12. Changed study email from stressreductionstudy@challiance.org to stressreduction@challiance.org.

Version Number 2.6
Version Date: 3/4/21
Summary of Revisions Made:

1. Specified use of Facebook advertisement for recruitment.
2. Modified weekly survey to include Buprenorphine dosage and concomitant medication prescriptions.
3. Added credibility/expectancy surveys to assess for treatment expectations.
4. Specified that participants who received a study smartphone must return the phone to the study team to be eligible to receive the study completion bonus.
5. Clarified in schedule of evaluations that oral-fluid toxicology will be assessed monthly for the first 12-weeks of the study.
6. Added “Droplr” as a HIPAA compliant screenshotting tool which will be used to document oral fluid toxicology screen results.
7. Specified use of password-protected REDCap survey links.
8. Added 48 hour completion bonus for large survey batteries.
9. Updated ATLAS.ti version from 7.0 to 8.4.4.
10. Corrected grammatical errors throughout protocol.
11. Clarified that qualitative interviews will be conducted for 30-60 minutes.
12. Replaced self-compassion scale short form with full self-compassion scale.
13. Removed FFMQ, ISMI, MAIA subscales 2 and 3.

14. Moved CAT-MH scheduling to occur at baseline, 12 weeks, and 24 weeks.
15. Corrected oral fluid toxicology screen frequency on informed consent assessment.
16. Replaced full MOCA with MOCA-BLIND.
17. Expanded study referral form to include clinician contact information, and patient scheduling information.
18. Removed Farah Samawi from study team roster.
19. Corrected payment information on ICF assessment.
20. Moved demographics survey from baseline battery to screening battery.
21. Specified that participants can request that attendance and oral fluid toxicology results be mailed to them on a monthly basis.

Version Number 2.7
Version Date: 5/18/21
Summary of Revisions Made:
1. Added additional information about Facebook advertisements
2. Clarified contact with buprenorphine prescriber
3. Clarified contact with referring provider
4. Clarified portions of adverse event protocols

Version Number 2.8
Version Date: 6/14/21
Summary of Revisions Made:
1. Removed Ian Concannon from study team roster
2. Added Leah Howard to the study team roster as a research coordinator
3. Added Paula Gardiner to the study team roster as a M-ROCC group leader and a Co-Investigator
4. Added Colleen LaBelle to the study team roster as the Boston Medical Center Site PI
5. Added Boston Medical Center as a relying site
6. Modified study-related injury section of the informed consent form as per request by Boston Medical Center

Version Number 2.9
Version Date: 7/22/21
Summary of Revisions Made:
1. Clarified that BMC staff will not engage in any recruitment, screening or consenting tasks, but will instead provide study team contact information to their patients.

Version Number 2.10
Version Date: 8/24/21
Summary of Revisions Made:
1. Removed Audrey Cabral from the study team roster
2. Removed Emily Tavanese from the study team roster
3. Added Joshua Phillips to the study team roster as a research assistant
4. Added Sarah King-McKeon to the study team roster as a M-ROCC group leader

Version Number 2.11
Version Date: 9/9/21
Summary of Revisions Made:

1. Removed Mark Albanese from the study team roster.

Version Number 2.12
Version Date: 9/13/21
Summary of Revisions Made

1. Expanded recruitment to Connecticut, Florida, Maine, New Hampshire, New York, Rhode Island, Texas, and Vermont.
2. Removed contingency management lottery draw from protocol.
3. Added $5 study gift cards for each group attended.
4. Added $5 study gift cards for each bi-weekly period in which all weekly surveys and oral fluid tests have been completed.
5. Added Instagram advertisement capacity and expanded advertisement variations.
6. Added FFMQ to survey battery.
7. Removed state specific language from provider email.
8. Included a letter template version of the provider email.
9. Clarified that the study groups are interventions/programs rather than a treatment that must be provided by a licensed clinician.
10. Specified that control group leaders will require 1 year of prior experience leading recovery support groups for patients with OUD.
11. Updated ICF title to more accurately reflect presence of treatment in the study.
12. Updated suicidality management plan.

Version Number 2.13
Version Date: 10/8/21
Summary of Revisions Made:

1. Added subtitle specifying change in study design from primary care phase to live-online focus.
2. Specified that oral fluid toxicology screen results will not be shared with a participant's Buprenorphine prescriber.
3. Specified how Instagram advertisements will be managed.
4. Removed duplicate reference to Massachusetts as a recruitment state.
5. Changed inclusion criteria to focus on participant state of residence rather than the location of their Buprenorphine prescriber.
6. Edited Figure 1 to reflect additional recruitment sources.
7. Specified that all advertisements will need approval by the IRB prior to their use.
8. Specified from which account Facebook and Instagram advertisements will originate.

Version Number 2.14
Version Date: 11/18/21
Summary of Revisions Made:

1. Added four new participant flyer versions
2. Enabled control participants to undergo a qualitative interview.
3. Shortened phone pre-screening survey.

4. Updated ICF assessment to reflect accurate payment information.
5. Added the “HEARD” contact information database as a recruitment source.
6. Added shortened versions of Facebook advertisements.

Version Number 2.15
Version Date: 2/28/22
Summary of Revisions Made:

1. Removed dollar sign graphic from study flyers.
2. Clarified purpose and data storage procedures of the “HEARD” database.
3. Clarified that Facebook interests will not be used in advertisements.
4. Changed order of phone pre-screening form.
5. Added Stiven Topalli to the study team roster.
6. Added Omnia Eldoghry to the study team roster.
7. Specified qualitative interview themes for the control arm.

Version Number 2.16
Version Date: 7/11/22
Summary of Revisions Made:

1. Removed Sasha Oxnard from the study team roster.
2. Removed Dave Roll from the study team roster.
3. Removed Caitlyn Wilson from the study team roster.
4. Removed Alex Brunel from the study team roster.
5. Added Gareth Parry to the study team roster.
6. Added Ashley Rector to the study team roster.
7. Added Hannah Goodman to the study team roster.
8. Added Elizabeth Gracey to the study team roster.

Version Number 2.17
Version Date: 8/2/22
Summary of Revisions Made:
1. Added Teresa Yeh to the study team roster.
2. Removed Kayley Okst from the study team roster.
3. Removed Leah Howard from the study team roster.
4. Removed Timothy Creedon from the study team roster.

Version Number 2.18
Version Date: 8/25/22
Summary of Revisions Made:
1. Added Javier Barria to the study team roster.
2. Removed Ashley Mallon from the study team roster.
3. Extended the study to new states including California, North Carolina, Alabama, Arizona, Virginia, Illinois, and Michigan.
4. Adding an apartment/mailbox number and zip code to the demographics form since they need this information to send packages to participants.
5. Changing the phone number on the flyer.

Version Number 2.19
Version Date: 12/06/22
Summary of Revisions Made:
1. Added Tori Blot to the study team roster.
2. Added Lolita Roland to the study team roster.
3. Removed Megan Edge from the study team roster.
4. Removed Gabriella Conversano from the study team roster.
5. Removed Esteban da Cruz from the study team roster.
6. Removed Stiven Topalli from the study team roster.

Version Number 2.20
Version Date: 01/06/23
Summary of Revisions Made:
1. Added Danielle La Camera to the study team roster.
2. Added Acelya Aslan to the study team roster.
3. Increased the number of the maximum enrolled participants
4. Added the study measure PhenX_A to the 24-week large survey battery

Version Number 2.21
Version Date: 05/22/23
Summary of Revisions Made:
1. Added Diane Joss to the study team roster.
2. Removed Laura Holland from the study team roster.
3. Removed Sarah Moore from the study team roster.

Table of Contents

TEAM STUDY ROSTER **17**
PRÉCIS **21**
1. STUDY OBJECTIVES **24**
1.1 PRIMARY OBJECTIVE **24**
1.2 SECONDARY OBJECTIVES 25
1.3 EXPLORATORY AIMS: 25
2. BACKGROUND AND RATIONALE 255
2.1 BACKGROUND ON CONDITION, DISEASE, OR OTHER PRIMARY STUDY FOCUS **25**
2.2 STUDY RATIONALE **32**
3. STUDY DESIGN 33
4. SELECTION AND ENROLLMENT OF PARTICIPANTS 35
4.1 INCLUSION CRITERIA 35
4.2 EXCLUSION CRITERIA 35
4.3 STUDY ENROLLMENT PROCEDURES 36

5. STUDY INTERVENTIONS 40
5.1 INTERVENTIONS, ADMINISTRATION, AND DURATION 40
5.2 HANDLING OF STUDY INTERVENTIONS 45
5.3 CONCOMITANT INTERVENTIONS 46
5.3.1 ALLOWED INTERVENTIONS 46
5.3.2 REQUIRED INTERVENTIONS 46
5.3.3 PROHIBITED INTERVENTIONS 46
5.4 ADHERENCE ASSESSMENT 46
6. STUDY PROCEDURES 48
6.1 506.2
506.2.1
506.2.2 ENROLLMENT, BASELINE, AND/OR RANDOMIZATION
51
6.2.3 BLINDING 54
6.2.4 FOLLOW-UP VISITS 55
6.2.5 COMPLETION/FINAL EVALUATION 56
6.2.6 QUALITATIVE INTERVIEWS 58
7. SAFETY ASSESSMENTS 59
7.1 SPECIFICATION OF SAFETY PARAMETERS 60
7.2 METHODS AND TIMING FOR ASSESSING, RECORDING, AND ANALYZING SAFETY PARAMETERS 60
7.2.1 607.2.2
637.3
637.4
647.5
677.6
678. INTERVENTION DISCONTINUATION
68
9. STATISTICAL CONSIDERATIONS 71
9.1 719.2
719.3
729.4
729.5
739.5.1
739.5.2
739.6
7410. DATA COLLECTION AND QUALITY ASSURANCE
78

10.1 78**10.2**
87**10.3 QUALITY ASSURANCE**
91
10.3.1 91**10.3.2**
92**10.3.3**
92**10.3.4**
93**10.3.5**
93**11. PARTICIPANT RIGHTS AND CONFIDENTIALITY**
94
11.1 94
11.2 94
11.3 95
11.4 96**5**
12. COMMITTEES **96**
13. PUBLICATION OF RESEARCH FINDINGS 97
14. REFERENCES **96**

2. TEAM STUDY ROSTER

Role (e.g., PI, Co-I, coordinator)	Name (include degree)	Institution	Phone #	Pager #	Status
PI	Zev Schuman-Olivier, MD	Cambridge Health Alliance	617-591-6056	617-546-1609	Active
Co-I, Quantitative Methodologist	Benjamin Cook, PhD, MPH	Cambridge Health Alliance	617-806-8741		Active
Co-I, Research Scientist / MTPC Supervisor	Richa Gawande, PhD	Cambridge Health Alliance	617-591-6055		Active
Co-I, M-ROCC Clinical Director	Alaine (Kiera) Fredericksen LICSW	Cambridge Health Alliance	617-591-6439		Active
Co-I, CMC Medical Director/MTPC Supervisor	Todd Griswold	Cambridge Health Alliance	617-591-6187	978-761-7380	Active
Co-I, GBOT Specialist	Randi Sokol, MD	Cambridge Health Alliance	781-338-0500	215-873-7771	Active
Co-I, Primary Care Addictions Specialist	Ellie Grossman, MD	Cambridge Health Alliance	617-591-6300	617-546-0500	Active
Data Analyst	Lydia Smith	Cambridge Health Alliance	781-850-6592		Active
Research Coordinator	Alexandra Comeau	Cambridge Health Alliance	617-591-6055		Active
Site PI -- Lynn	Dr. Annalee Wells, MD	Lynn Community Health Center	781-581-3900		Active
Co-I, Neurocognitive Consultant	Nancie Rouleau, PhD	Cambridge Health Alliance	(418) 663-5741		Active
Co-I, Clinical Trials Consultant	Roger Weiss, MD	Cambridge Health Alliance	617-855-2242		Active
M-ROCC Group Leader	Dr. Bari-Sue Brodsky, MD	Cambridge Health Alliance	978-532-4903		Active
Research Assistant	Joseph Rosansky	Cambridge Health Alliance	617-575-5802		Active
Control Group Leader	Connor Stuart	Cambridge Health Alliance	617-591-8567		Active
Site PI – Boston Medical Center	Colleen LaBelle	Boston Medical Center	617-797-6712		Active
Co-I, M-ROCC Group Leader	Dr. Paula Gardiner, MD	Cambridge Health Alliance	781-929-6460		Active
M-ROCC Group Leader	Sarah King-McKeon	Boston Medical Center	(617) 983-3680		Active
Research Assistant	Joshua Phillips	Cambridge Health	617-851-8625		Active

		Alliance			
Research Assistant	Omnia Eldoghry	Cambridge Health Alliance	(781) 475-3985		Active
Data Analyst	Gareth Parry	Cambridge Health Alliance	617-851-8625		Active
Research Coordinator	Elizabeth Gracey	Cambridge Health Alliance	617-851-8625		Active
Research Coordinator	Hannah Goodman	Cambridge Health Alliance	617-851-8625		Active
Research Assistant	Ashley Rector	Cambridge Health Alliance	617-851-8625		Active
M-ROCC Group Leader	Teresa Yeh	Cambridge Health Alliance	617-575-5985		Active
Data Analyst	Javier Barria	Cambridge Health Alliance	617-851-8625		Active
Research Coordinator	Tori Blot	Cambridge Health Alliance	617-851-8625		Active
M-ROCC Group Leader	Lolita Roland	Cambridge Health Alliance	617-851-8625		Active
Research Assistant	Danielle La Camera	Cambridge Health Alliance	617-851-8625		Active
Research Assistant	Acelya Aslan	Cambridge Health Alliance	617-851-8625		Active

<u>Personnel virtually present during study procedure:</u>
At least one of the following personnel will be virtually present during study and group procedures:
Zev Schuman-Olivier, MD, Principal Investigator; Richa Gawande, PhD, Alaine Fredericksen, LICSW, Site-PI, or research coordinators (Hannah Goodman, Elizabeth Gracey) or research assistants (Joseph Rosansky, Joshua Phillips, Omnia Eldoghry, Ashley Rector, Tori Blot, Acelya Aslan, Danielle La Camera).

<u>Study team members responsible for the following activities:</u>
At least one of the following personnel will be responsible for obtaining and documenting informed consent: Zev Schuman-Olivier, MD, Principal Investigator, Alaine Fredericksen, LICSW, Richa Gawande, PhD, research coordinators (Hannah Goodman, Elizabeth Gracey), or research assistants (Joseph Rosansky, Joshua Phillips, Acelya Aslan).

The following personnel will be responsible for providing on-going information to the study sponsor and the IRB: Zev Schuman-Olivier, MD, Principal Investigator.

At least one of the following personnel will be responsible for maintaining participants' research records: Zev Schuman-Olivier, MD, Principal Investigator, Research Coordinators (Hannah Goodman, Elizabeth Gracey).

Inclusion of study personnel:

The research team would consist of the following: Zev Schuman-Olivier, MD (PI); Benjamin Cook, PhD who is a quantitative methodologist and Director of the Health Equity Research Lab, Cambridge Health Alliance/Harvard Medical School; Richa Gawande, PhD (Research Scientist and MTPC supervisor); Randi Sokol, MD who is a primary care physician with expertise in Group-Based Opioid Treatment (GBOT); Alaine Fredericksen, an experienced addictions social worker who has been leading MTPC groups and is clinical director for M-ROCC, and site PIs who also provide clinical administrative leadership from Lynn Community Health (Dr. Annalee Wells). Nancie Rouleau, PhD is a neuropsychologist with expertise conducting research with neurocognitive tasks. Additional staff include Todd Griswold, MD (CMC Medical Director/MTPC Supervisor); Ellie Grossman, MD (Primary Care Addictions Specialist); Alexandra Comeau, MA (Research Coordinator); Roger Weiss, MD (Clinical Trials Consultant), Bari-Sue Brodsky, MD (M-ROCC Group Leader), Paula Gardiner, MD (M-ROCC Group Leader and Co-Investigator), Colleen LaBelle (Site – PI BMC) Joseph Rosansky (Research Assistant), Connor Stuart (Control Group Leader), Joshua Phillips (Research Assistant), Sarah King-Mckeon (M-ROCC Group Leader), Omnia Eldoghry (Research Assistant), Hannah Goodman (Research Coordinator), Elizabeth Gracey (Research Coordinator), Ashley Rector (Research Assistant), Gareth Parry (Data Analyst), Teresa Yeh (M-ROCC Group Leader), Tori Blot (Research Coordinator), Lolita Roland (M-ROCC Group Leader), Acelya Aslan (Research Assistant), Danielle La Camera (Research Assistant), and Diane Joss (Research Scientist).

PARTICIPATING STUDY SITES

The study will be conducted at the CHA Center for Mindfulness and Compassion with online recruitment **(**DUNS number: 805262995**).**

The following affiliated sites from collaborating institutions that offer primary care office-based opioid treatment will contribute to online recruitment efforts and provide trained intervention group co-leaders. We will also recruit from other interested Office-Based Addiction Treatment (OBAT) sites in Massachusetts, Connecticut,

Florida, Maine, New Hampshire, New York, Rhode Island, Texas, Vermont, California, North Carolina, Virginia, Alabama, Arizona, Illinois, and Michigan, but referring providers from these other sites will not be part of the study team. The 3 non-CHA affiliated study sites will be added through SMART IRB reliance agreements, and no site will be involved in study tasks until its corresponding reliance agreement has been executed.

Cambridge Health Alliance (DUNS number: 805262995**)**
CHA Central Street Care Center (26 Central St, Somerville MA 02143)
CHA Revere Care Center (454 Broadway, Revere, MA 02151)

Lynn Community Health Center (DUNS number: 030834915), 269 Union St; Lynn, MA 01901

Boston Medical Center (DUNS Number: 00-549-2160), 801 Massachusetts Avenue, Boston MA 02118

North Shore Community Health Salem (DUNS number: 144117538), 47 Congress Street Salem, MA 01970

To maximize efficiency and consistency across participating sites, we will utilize a single IRB (sIRB) to streamline the IRB review and approval of the research. The Cambridge Health Alliance Institutional Review Board (CHA IRB) will act as the sIRB for all sites. The PI has worked closely to obtain approval for several clinical trials in the past with CHA IRB, both for primary care and OUD populations.

CHA will utilize SMART IRB (www.smartirb.org) to document reliance and all participating sites will cede to CHA IRB. SMART IRB is a national IRB Reliance Agreement that contains many features to aid in the acceptance, coordination, and implementation of a single IRB review model in a highly cost-effective manner. Each institution and site investigator retain responsibility for the conduct of research at their site. As such, investigators at relying sites must provide relevant information to their local IRB, if there is one, for institutional review. Institutional review of each site’s local requirements (e.g., human subject training requirements, conflict of interest, and consent forms to ensure locally required language is included for compensation for injury, conflict of interest, etc.) and “non-IRB” institutional reviews by ancillary committees (e.g., institutional biosafety, radiation safety) must be performed by each institution, as applicable.

The BMC team will not obtain verbal consent from participants to share their contact information with the study team. Instead, the BMC team will distribute IRB approved flyers throughout the center, and participants will be given the option to self-refer. BMC will also provide study team members who will act as group leaders for the M-ROCC arm of the study. Context surrounding adverse events will be shared with the relevant BMC team members, as is outlined in protocol section 7.5.

3. PRÉCIS

Study Title
Effect of Mindfulness Training on Opioid Use and Anxiety During Primary Care Buprenorphine Treatment

Objectives

We will conduct a randomized controlled trial comparing a live-online Mindful Recovery Opioid Care Continuum (M-ROCC) group (a motivationally-responsive, trauma-informed extended 24-week Mindfulness-Based Intervention (MBI) with an ascending practice dose ladder approach that offers mindfulness training instruction optimized to enhance self-regulation and catalyze recovery) with a live-online control group, to examine the efficacy of M-ROCC on abstinence from illicit opioids while also targeting co-morbid anxiety, pain, and substance use (cocaine, benzodiazepines (BZD)).

Design and Outcomes

This will be a RCT designed to compare live-online M-ROCC groups with a live-online control group on the primary outcome of
the number of biochemically confirmed illicit opioid negative abstinent periods (defined by a negative oral fluid test [negative for opiate, oxycodone, fentanyl, methadone] AND no self-reported illicit opioid use) during weeks 13-24 of study (six two-week periods of opioid abstinence during the final 12 weeks of the study).

.

Clinical secondary outcomes include level of anxiety measured by the Patient Reported Outcomes Measurement Information System – Anxiety Short Form 8a (PROMIS-ASF), level of pain interference measured by the PROMIS Pain Interference Scale (PROMIS-PISF), and the number of positive oral fluid tests for BZD or cocaine during the final 12 weeks of the study. Other exploratory outcomes will be level of anxiety measured by the Beck anxiety inventory (BAI), 24-week intervention retention, as well as mechanisms of self-regulation assessed by self-

report and behavioral measures (emotion regulation, decentering/metacognitive monitoring, interoception, experiential avoidance, self-critical rumination, and self-compassion) and their mediating effects on anxiety and opioid abstinence. Qualitative interviews will be conducted with a minimum of 12 and a maximum of 30 M-ROCC or control group completers until thematic saturation to examine themes regarding live-online mindfulness delivery and to compare responses with our R21 qualitative outcomes from our in-person M-ROCC group model. Computerized Adaptive Testing for Mental Health (CAT-MH) will be used to assess changes in psychiatric co-morbidity. Finally, exploratory outcomes of stigma, mindfulness, perceived stress, pain catastrophizing, interpersonal conflict, and shared identity within group will be measured.

Interventions and Duration

Live-Online M-ROCC:

The live-online M-ROCC program is directly adapted from Mindfulness Training for Primary Care (MTPC), which was developed and tested in the MINDFUL-PC research study and piloted for OUD in the R21 study. M-ROCC is based on a model of human experience which identifies experiential avoidance and disconnection from our bodies and from others as fundamental causes of human distress, and addictive behavior in particular. M-ROCC is a 6-month long Mindful Recovery OUD Care Continuum designed with the clinical needs of OUD participants to be delivered in primary care clinics. Due to COVID-19, this study will test the curriculum delivered in a virtual setting online as a research intervention separate from clinical care. Below we describe the adjustment of the program with logistical constraints of the COVID-19 pandemic in mind. The M-ROCC curriculum focuses on integration of mindfulness practice for living well through stress, anxiety, depression, pain and addiction recovery. The M-ROCC curriculum has three primary components.

1) LDM (Low-Dose Mindfulness): A four-week introductory mindfulness program for OUD with monthly rolling admission, which includes 50 minutes of mindfulness introduction and explicit training in the use of mobile mindfulness apps. LDM introduces participants to the ascending practice dose ladder. The live online session will be 80-90 minutes with 30 minutes of group check-in and online toxicology testing with staff followed by 60 minutes of the mindfulness program.

2) MTPC-OUD (Mindfulness Training for Primary Care – OUD): A sixteen-hour curriculum delivered in either (a) eight-week intensive mindfulness group with an

ascending practice dose ladder and skill integration over the course of the eight weeks, 120 minutes per session, or (b) sixteen-week intensive mindfulness group with an ascending practice dose ladder and skill integration over the course of sixteen weeks with 60 minutes per session. While the LDM group format will be delivered in the same manner in all study cohorts, the MTPC-OUD curriculum format will be interchangeable in different cohorts depending on the implementation needs of group leaders. The live online session will be 80-90 minutes with 30 minutes of group check-in and online toxicology testing with staff followed by 60 minutes of the mindfulness program.

3) MCS (Mindfulness Maintenance Check-in Support): Various ongoing weekly mindfulness continuation groups with check-in and reminders, leveraging mobile mindfulness application and motivational-oriented counseling for graduates of LDM for 50 minutes of mindfulness practice. MCS will be available for the duration of the study. After LDM completion, 20 weeks is the longest expected duration of MCS for a participant who goes directly from LDM to MCS and does not choose to enroll in MTPC-OUD. Free MCS groups will be available through the CMC Free Live Online Community Program.

Within the M-ROCC curriculum there is flexibility, so that each participant who enrolls will begin in LDM for four weeks. If the participant is able to develop a home practice and begins ascending the practice ladder, then they will be given the option to move to the intensive MTPC-OUD or the ongoing MCS depending on readiness. Participants can repeat LDM, transfer to the MCS group or transition from the LDM or MCS into intensive MTPC, forming a continuous practice and check-in support continuum that encourages increased practice with the spirit of autonomy, collaboration, and naturalistic selection based on participant motivation and capacity.

<u>Live-Online Control Group:</u>

The live-online control group will be attention- and time-matched to the M-ROCC LDM and MCS groups. This weekly control group will run for 80-90 minutes, with 30 minutes of group check-in and online toxicology testing with staff followed by 50-60 minutes of group time. As an active group comparator, this group will isolate mindfulness components by controlling for the therapeutic aspects of a group. Group content is derived from commonly used methods in community substance use disorder treatment and recovery groups, including elements of Motivational Interviewing, CBT, Community Reinforcement, and Twelve Step Facilitation, as well as educational and didactic components. These live-online control groups will function as research groups only and will not replace standard of care at sites, but they may provide a therapeutic benefit to participants lacking a recovery group due to

the constraints of COVID-19.

Sample Size and Population

We anticipate an approximate enrollment of N=236 with a maximum of N=280 participants prescribed buprenorphine from Connecticut, Florida, Maine, Massachusetts, New Hampshire, New York, Rhode Island, Texas, Vermont, California, North Carolina, Virginia, Alabama, Arizona, Illinois, or Michigan over eight months. We anticipate enrolling approximately 30 participants per month. We anticipate 192 (81%) participants to be randomized (1:1 ratio, block size of 4, 6, 8 to M-ROCC or live-online control, respectively) in online groups (n=96 M-ROCC, n=96 Control), and of these we anticipate 45 (47% of those who began LDM) will begin the 16 session MTPC-OUD intensive group. This estimate is based on our experience in the R21 Mindful-OBOT pilot, in which 47% of participants enrolled in our initial pilot site began an MTPC-OUD group.

Participants will be patients living in U.S. states approved by the IRB for recruitment who are prescribed Buprenorphine. We will recruit from one of the three non-CHA affiliated sites from collaborating institutions that offer primary care office-based opioid treatment, from two sites affiliated with CHA, and from OBAT sites in U.S. states approved by the IRB for recruitment, and also via Facebook and Instagram. Participants must be 18-70 years old and must be able to use an electronic device with a video camera to attend study groups and complete questionnaires. This study will enroll individuals of any gender, and any demographic group, including pregnant women.

Participants must have a diagnosis of opioid use disorder, be prescribed buprenorphine, have sufficient English fluency to understand procedures and questionnaires, and be without acute severe mental illness (mania, psychosis, suicidality with intent/plan) and ability to provide informed consent. This trial is limited to English-speaking participants at this time since the intervention curricula have not been adapted to other languages.

1. STUDY OBJECTIVES

1.1 Primary Objective

1.1.a. Opioid Abstinence: Compare effects of live-online M-ROCC vs. a live-online control group on the number of biochemically confirmed illicit opioid negative abstinent periods (defined by negative oral fluid tests [negative for opiate, oxycodone, fentanyl, methadone] AND no self-reported illicit opioid use) during weeks 13-24 of study.

H1a: M-ROCC will have more abstinent time periods than the control group during weeks 13-24.

1.2 Secondary Objectives

1.2.a. Clinical Co-morbidity: Compare effects of M-ROCC vs. the control arm on anxiety and pain.

H2a: Among those with anxiety disorders, M-ROCC will have greater reduction in anxiety than control group at 24 weeks.
H2b: M-ROCC will have greater reduction in pain interference than control group at 24 weeks.

1.2.b. Cocaine and Benzodiazepine Use: Compare effects of live-online M-ROCC vs. the control arm on cocaine and benzodiazepine use.

H3a: M-ROCC will have fewer positive oral fluid tests for cocaine than a control group during weeks 13-24.
H3b: M-ROCC will have fewer positive oral fluid tests for BZDs than a control group during weeks 13-24.

1.3 Exploratory Aims:

We will examine the impact of M-ROCC randomization status and mindfulness practice dose on intervention retention at 24 weeks, substance craving, opioid craving, psychiatric co-morbidity (Computerized Adaptive Testing for Mental Health; CAT-MH), and self-regulation targets including experiential avoidance, self-compassion, emotion regulation, interoceptive regulation, self-critical rumination, perceived stress, and interpersonal conflict, assessed at baseline and weeks 8, 16, and 24.

2. BACKGROUND AND RATIONALE

2.1 Background on Condition, Disease, or Other Primary Study Focus

Opioid use disorder (OUD) and overdose due to opioid-induced respiratory depression is a serious public health issue with 42,249 opioid overdose deaths in 2016, which was a 5-fold increase from 1999[1]. Driven initially by two decades of increasing prescription opioid use[2] and followed by a population transition from heroin, starting in 2011[3], the "opioid overdose epidemic" has intensified[4].

Office-based opioid treatment (OBOT) with buprenorphine/naloxone (B/N) has emerged as a popular evidence-based treatment for OUD[5]. Buprenorphine (BUP) is a partial opioid mu-receptor agonist that prevents opioid withdrawal, blocks opioid euphoria[6] and prevents opioid overdose[7].

About 1/3 of BUP-waivered prescribers nationally are primary care providers (PCP)[8]. Most US counties do not have a waivered provider to prescribe B/N, and in many counties, PCPs are the only access point. Therefore, the development of disseminable interventions that help PCPs address patient psychosocial stress and psychiatric symptoms during OBOT may improve access to care and prevent overdose deaths.

Retention in B/N treatment is important because dropout from opioid substitution treatment is associated with relapse[9] and an increased risk of overdose and death[10]. Only about 46-50% of patients are retained in B/N treatment at 24 weeks[11]. One major predictor of poor retention is psychosocial stress and psychiatric co-morbidity related to mental illness, e.g., anxiety[12], Post-Traumatic Stress Disorder (PTSD)[13]. Additionally, co-morbid cocaine use is a predictor of opioid use and attrition from treatment[14,15]. Also, increased pain and pain volatility among those with chronic pain syndromes are associated with increased opioid use during BUP treatment[16].

The COVID-19 pandemic also threatens Office-Based Opioid Treatment (OBOT) by restricting access to in-person treatment for co-morbidity that can impact BUP treatment outcomes. The pandemic has led to nationwide changes in OUD treatment. Many primary care facilities are presently unable to hold in-person Group Based Opioid Treatment (GBOT) groups. Therefore, it is crucial that these services be delivered in a feasible online format such that OUD patients can still receive the quality care they need.

More than 30% of patients with OUD get benzodiazepine (BZD) prescriptions during the first year of opioid treatment[17]. Early in treatment, patients receiving B/N treatment frequently request a benzodiazepine prescription to mitigate anxiety and insomnia symptoms, to deal with psychosocial stress, and to regulate emotions[18]. Among those in OBOT who do not receive a BZD prescription, 67% still report a history of BZD misuse[19]. Mixing BZDs and opioids can lead to synergistic overdose[20], cognitive impairment[21], and accidental injuries[19]. Heavy BZD use reduced HCV antiviral treatment uptake[22]. Much concern and debate exists about BZD diversion within B/N programs[22]. Therefore, non-pharmacologic approaches to anxiety, stress, and emotional dysregulation are needed.

Unfortunately, many B/N studies for OUD have reported limited benefit from behavioral interventions[23]. For instance, in a RCT (n=166), Feillin et al found no significant differences between medication management and a behaviorally-enhanced approach on percentage of opioid negative urine tests and only 45% of randomized patients completed the 24-week trial. In a second study, Fiellin et al examined the effect of adding 12 sessions of CBT to medication management in a 24-week study of opioid users in primary care (n=141) and again found no difference between groups on opioid use and

41% of subjects completed the trial. Ling et al tested Cognitive Behavioral Therapy (CBT), contingency management, and their combination against medication management alone, finding no specific differences between group and 50% completed the 32-week trial. Finally, Weiss et al conducted the Prescription Opioid Addiction Treatment Study (POATS) trial at 10 NIDA Clinical Trials Network sites (n=360), comparing medical management with individual drug counseling plus medication management after 12 weeks of B/N stabilization, finding no group differences. Considering the failure of these established behavioral therapy approaches to impact key opioid use disorder outcomes, there is a need to develop and evaluate the impact of innovative, evidence-based, behavioral therapies with a different theoretical approach, such as Mindfulness-Based Interventions (MBI)[23–25].

Since stress, pain, anxiety, and depression are frequently co-morbid with OUD, alternative behavioral interventions that successfully target those symptoms could be promising. Mindfulness-based interventions (MBI) have been developing for the past 30 years with an initial focus on chronic pain[26,27], stress reduction[28], and anxiety disorders[29]. MBIs start with present-moment awareness without judgment and focus on mindfulness practice as a mechanism for change[30]. The methodology and treatment fidelity of MBIs are becoming increasingly refined and mindfulness has emerged as a commonplace intervention in US medical schools[31]. Interventions that involve mindfulness meditation are attractive because of reported impact from MBIs on co-morbid symptoms of anxiety[32], depression[33], and pain[34–36], based on findings from meta-analyses. Mindfulness also has been shown through meta-analysis to decrease physiologic markers of stress[37], which is important because stress can be a trigger for craving[38], relapse[39], addiction persistence[40], and stress system dysregulation is a problem among OUD users[41,42]. Stress is also linked with anxiety and depression, as well as exacerbations of mental illness, such as bipolar disorder[43] and schizophrenia[44]. Mindfulness has also been shown to have a mild effect on insomnia and can serve as an auxiliary treatment for sleep complaints[45].

Even as mindfulness emerged as a mental health treatment, the role for mindfulness in medication-assisted treatment (MAT) for OUD remained unclear. When we started researching mindfulness and OUD in 2004, the initial aim was to assess whether mindfulness meditation was acceptable and feasible during MAT, and whether it could impact behavior. The first mindfulness-oriented randomized controlled trial (RCT) during MAT for OUD was a NIDA-funded trial of Spiritual Self Schema therapy by Margolin et al and delivered by Dr. Schuman-Olivier who is PI, for reducing HIV risk among methadone-maintained cocaine users. This intervention focused on integrating CBT schema therapy with mindfulness meditation. The initial pilot RCT (n=72) found reductions in HIV-risk behaviors (OR: = 8.89; CI: 95% = 1.62-48.93) after 8 weeks of mindfulness compared with controls[46]. A feasibility pilot among HIV+ cocaine users

maintained on methadone (n=38) demonstrated reduced impulsivity after 12 weeks and increased motivation for drug abstinence, HIV risk prevention, and medication adherence[47]. Using an ascending practice dose ladder approach, this study found that 64% reported greater than 30 min/day of practice by 12 weeks. While some recent feasibility studies have been published[48], since this initial MAT RCT in 2004, no federally-funded RCTs have been published from a MBI within buprenorphine or methadone treatment.

Even though there is limited evidence among OUD MAT, Mindfulness Based Relapse Prevention (MBRP) has been well-studied for substance use disorders (SUD) in general. Among a sample of primarily alcohol and stimulant users, MBRP demonstrated relative efficacy at 12 months with fewer substance use days and decreased heavy drinking compared to relapse prevention and treatment as usual[49]. Higher levels of home mindfulness practice were associated with lower alcohol and other drug use and craving, but the benefits faded during months 2-4, suggesting a longer trial or additional support could be helpful[50]. In addition, this RCT population was recruited during after-care post-detox and only had 9% of the sample with OUD.

Through multiple pilot studies of MBRP, several mechanisms of action of mindfulness have been elucidated, which could be applicable to addiction treatment outcomes[51,52]. Mindfulness training reduces negative affect and psychiatric impairment, which was effective in reducing substance use[53], through decoupling the association between negative affect and substance use craving[54]. In summary, MBRP has established the relative efficacy of mindfulness for SUD but has yet to be extended to patients with OUD prescribed BUP, and the effects of home practice seem to wear off quickly after the 8-week program ended. In addition, meta-analysis of 9 MBRP studies suggested that while MBRP reduced craving symptoms, it did not impact substance use outcomes[55]. Therefore, the standard MBRP 8-week program does not appear to have sustained impact on substance use, suggesting longer mindfulness interventions or enhanced programs are needed to get the full benefit from the intervention.

Other MBIs have also been shown to engage mechanisms potentially relevant to addiction treatment outcomes[56,57]. For instance, a RCT of Mindfulness Training for Smokers reported reduction in tobacco use after 4 weeks of intensive mindfulness training, and demonstrated decoupling of the relationship between craving and smoking[58]. This program integrated mindfulness theory with a model of operant conditioning to intensify the application of mindfulness to behavior change[59]. Mindfulness Oriented Recovery Enhancement (MORE) was designed for reducing opioid misuse among people on opioid therapy for chronic pain, adding innovative exercises like mindful savoring. Patients in MORE had less momentary pain and reported more positive affect[60]. Opioid misusers had decreased attentional bias, which was also associated with

greater perceived control over the pain and attenuated emotional reactivity[61]. Finally, self-compassion has emerged as a protective factor for substance use[62] and is a mechanism for reducing anxiety and depressive symptoms[63]. The Mindful Self-Compassion program has emerged as the 4th most common MBI nationally[31], and can be particularly helpful with the impact of toxic shame[64], which can drive substance use and relapse[65,66]. As Wilson et al point out, an intervention that could incorporate new innovative developments in the mindfulness field, while letting go of the requirement for the standard time format, leveraging mobile technology, and delivering guidance in a motivationally appropriate way during different points in the change process, could offer a more impactful intervention for substance use disorders[57].

As part of the Mindfulness Research Collaborative, a network of researchers funded by the NIH Science of Behavior Change (SOBC) Initiative[67], we have been involved in an ongoing process of systematic review and meta-analysis, examining the relationship between mindfulness practice, self-regulation targets and health behavior change. This process has developed on our original model[68], identifying evidence for three broad self-regulation domains targeted by mindfulness meditation, including cognitive control processes, emotion regulation, and self-related processes.

(A) Cognitive Control processes (COG) include attention (i.e. orienting, alerting[69,70], vigilance[71], attentional lapses[72]); executive function, conflict monitoring[73,74], impulsivity and inhibitory control[75,76], and metacognitive awareness[77–79]/decentering[80,81].. Studies indicate that meditation training, such as MBIs, engage these cognitive processes[82,83,92–95,84–91].

(B) Emotion regulation (EMO), which is the capacity to alter the magnitude or duration of an emotional response[96]. Poor emotion regulation impairs the capacity for self-regulation behaviors that support addiction recovery[97]. MBIs favorably engage measures of emotion regulation such as amygdala activation[87,98–101], sympathetic hyperarousal[88,102–106], and emotional responses to stressful situations[106–114].

(C) Self-related processes (SRP), including: *(i) self-efficacy* – the belief in one's capabilities to execute the courses of action required to manage prospective situations (such as coping with a trigger)[115,116], which is a central aspect of self-regulation and behavior change[117,118]; *(ii) self-compassion* – the capacity to extend compassion to oneself in instances of perceived inadequacy or failure, rather than engaging in self-destructive behaviors (self-judgment, isolation, rumination) or in permissive, risky behaviors[119–121]: self-compassion has been found to promote health behaviors such as adhering to diets[122], smoking cessation[123], physical activity[124], and seeking medical treatment when needed[125]; *(iii) self-related rumination or mind-wandering* – which may be beneficial in some cases[126–128], but can be detrimental when negatively-valenced or disrupting goal directed activity[79,98,128–133]; and *(iv) interoceptive awareness* – awareness of internal manifestations of emotions and feelings and inner body sensations[134–136],

which is considered fundamental to the 'experiencing self'[137–142], seems to be involved drug craving[143], urges and emotional decision-making[144], engages insula cortex activation[145,146], and is of major interest in MBI research[68,87,153–162,133,163,146–152].

Considering this self-regulation model and the innovative MBI trainings developed over two decades, we set out to develop an empirically supported MBI that would specifically engage these mechanistic self-regulation targets and enhance them to specifically support the initiation and maintenance of behavior change. As part of the SOBC Initiative, our laboratory has aimed to build on the first-generation Mindfulness-Based Program framework[30] and the fundamental teaching processes developed in Mindfulness-Based Stress Reduction and Mindfulness-Based Cognitive Therapy[164], while also learning from the multitude of specific MBI adaptations for SUD[56] with the goal to develop a MBI focused on harnessing mindfulness practice to catalyze behavior change among a broad primary care population. Using skills and exercises developed in empirically-supported evidence-based mindfulness-oriented addiction treatments, i.e., Mindfulness-Based Relapse Prevention, Mindfulness Training for Smokers, and Mindfulness-Oriented Recovery Enhancement as well as Mindfulness-Based Cognitive Therapy, Mindful Self-Compassion, and Motivational Interviewing[165], we have created a seamless and potentially more potent MBI: Mindfulness Training for Primary Care (MTPC). In addition, unlike other stagnant MBIs whose manual is fixed, MTPC, developed through the iterative SOBC process for the past 3 years, has been adapted to incorporate and enhance the strategies supported by empirical evidence about mechanistic self-regulation targets involved in behavior change. In two clinical trials, MTPC has reliably resulted in increased rates of health behavior change initiation[166]. Because of this capacity to focus on empirically-supported behavior change mechanisms and its integration of core exercises and concepts from earlier, addiction-focused MBIs, MTPC is ideally suited for a trial with OUD. In addition, because it is already designed for implementation in primary care, it can be tested in a real-world treatment setting where there is greater external validity than in closed research settings.

The M-ROCC program is directly adapted from Mindfulness Training for Primary Care (MTPC), based on a model of human experience which identifies experiential avoidance and disconnection from our bodies and from others as fundamental causes of human distress, and addictive behavior in particular. MTPC-OUD has 4 threads in addition to the core focus on present moment awareness that emerge in every session, including 1) **autopilot and mindful behavior change**, 2) **interpersonal mindfulness practice** to reduce interpersonal reactivity and self-regulation failures in social contexts[167] (e.g., corrosive couple conflict[168]) that can lead to substance use[169], 3) **common humanity and stigma reduction through kindness and self-compassion**, and 4) **autonomy and choice** through a group leader who plays a collaborative, co-participatory role, which is

trauma-informed[170,171] and motivationally-sensitive. Because trauma is common among patients with OUD[172], a trauma-informed mindfulness program that supports choice is important to prevent retraumatization[173]. MTPC emphasizes choice and trains group leaders to adapt sessions with the expectation that people in the group have trauma. MTPC has been shown to support behavior change among people with PTSD[166]. In MTPC-OUD, we adapted the standard daily practice dose for MTPC to start with 20-25 minutes a day instead of 40 minutes a day, with the goal of continuing the ascending practice dose ladder to attain 30-45 minutes of daily practice by the end of the 8-week intensive program. Based on 3 decades of science and practice of MBI development[30], MTPC was designed in an iterative process integrating strategies from other MBIs to empirically engage self-regulation target mechanisms to enhance the impact of mindfulness on behavior change outcomes: e.g., savoring practice helps address hedonic dysregulation among opioid-dependent patients[174]; urge surfing helps tolerate craving[175] and negative affective experiences[176]. MTPC also applies a mindfulness approach to values exploration[177,178] in a way that helps people develop discrepancy while supporting autonomy and confidence, consistent with processes from both Self-Determination Theory and Motivational Interviewing[179]. Finally, MTPC explicitly recognizes the ubiquity of medication in primary care and has modules designed to help people be mindful of experiences arising around medication[180,181]. This is particularly important in BUP treatment where people have a strong affective relationship with their medication and substantial internalized stigma about being on BUP[182,183]. Within MTPC, self-compassion is more explicitly cultivated to help reduce substance use risk[62], address the toxicity of shame and regret that can drive addiction relapse cycles[184], and weaken the impact of internalized stigma[185] related to BUP and seeking addiction treatment[186].

In addition to MTPC, we have also developed a low-dose mindfulness (LDM) intervention for primary care. The LDM approach provides brief didactic and experiential exposure to mindfulness and then leverages online resources and provides encouragement with ongoing practice reminders. In two primary care samples, we demonstrated LDM paired with smartphone mindfulness app links and reminders reliably resulted in stress reduction (d= -0.52; -0.58)[166,187]. In our R21 pilot study, the M-ROCC program including LDM and 16 hours of intensive MTPC curriculum demonstrated reductions in experiential avoidance, anxiety and pain interferences and increased mindfulness, self-compassion and interoceptive awareness ($p<0.05$). In addition, cocaine and benzodiazepine positive urine tests became less frequent over time in the program ($p<0.05$).

In conclusion, mindfulness shows promise as a behavioral intervention for OUD, because of its impact on self-regulation and co-morbid symptoms such as anxiety. Yet, standard approaches may lack the potency and may be either too long or too short for OUD patients based on the heterogeneity of their motivation upon entrance to B/N treatment. A

RCT comparing the efficacy of 24-weeks of a live-online Mindful Recovery Opioid Care Continuum (M-ROCC) group with 24-weeks of a live-online control group could have a major impact on clinical OUD treatment, and if successful, could also revolutionize MBI addiction research by demonstrating a stage-oriented, motivationally-informed program. A successful live-online group model of a behavioral intervention for OUD for patients prescribed buprenorphine through primary care office-based opioid practices in our region that reduced anxiety, enhanced treatment retention, and reduced illicit opioid use during buprenorphine treatment would be in high demand. Importantly, this model would likely be rapidly disseminated as a national model in primary care addiction treatment and could help reduce relapse and put a stop to the ever-increasing rate of overdose deaths.

2.2 Study Rationale

This study offers five innovations that will shift current research and clinical paradigms:

1. Motivationally-Responsive MBI: This is the first study to design and evaluate the impacts of a stage-oriented, motivationally-responsive, mindfulness continuum. M-ROCC starts with a focus on engagement and enhancing confidence, providing flexibility in training session type depending on participant motivation.

2. Ascending Practice Dose Ladder: The ascending practice dose ladder provides steady encouragement throughout and builds practice confidence and prevents early dropout from negative self-attributions.

3. Live-Online Delivery: No published RCT of online MBIs during BUP treatment exists. This format allows for MBIs to be delivered to patients prescribed BUP, even under the constraints of the COVID-19 pandemic.

4. Primary Care Focus: This is the first MBI program designed for primary care BUP treatment for OUD. Prior MBI studies focus on other substance use disorders or on methadone treatment even though primary care BUP in office-based opioid treatment is becoming the most common way that people get OUD treatment.

5. Optimized for Self-Regulation and Behavior: MTPC is optimized using a mechanistic self-regulation framework from the NIH Science of Behavior Change (SOBC) Initiative[168] to catalyze behavior change[166].

Developed through stakeholder collaboration, the continuum has been designed for participants to move through the three component phases at their own pace, allowing for flexible implementation and dissemination.

3. STUDY DESIGN

This will be a RCT comparing the efficacy of 24-weeks of a live-online Mindful Recovery Opioid Care Continuum (M-ROCC) group with 24-weeks of a live-online control group. Participants will be recruited from 3 affiliated sites from collaborating institutions that offer primary care office-based opioid treatment, in addition to 2 affiliated with CHA (see figure 1), as well as from OBAT sites in US states approved by the IRB for recruitment, and through Facebook and Instagram advertisements. The 3 non-CHA sites will execute SMART IRB reliance agreements with CHA and will not engage in study activities until their corresponding reliance agreements have been executed. We will enroll patients prescribed buprenorphine into a study that will be run remotely with assessments that can be completed fully online. Each study group will include patients prescribed buprenorphine. We will primarily recruit from various different primary care buprenorphine practices. Participants from various recruitment sites will come together for the live-online study groups. M-ROCC and live-online control groups will not replace standard of care at any sites and will instead be conducted as research groups.

Figure 1. Five affiliated primary care sites and non-affiliated recruitment sources in the MINDFUL-OBOT study

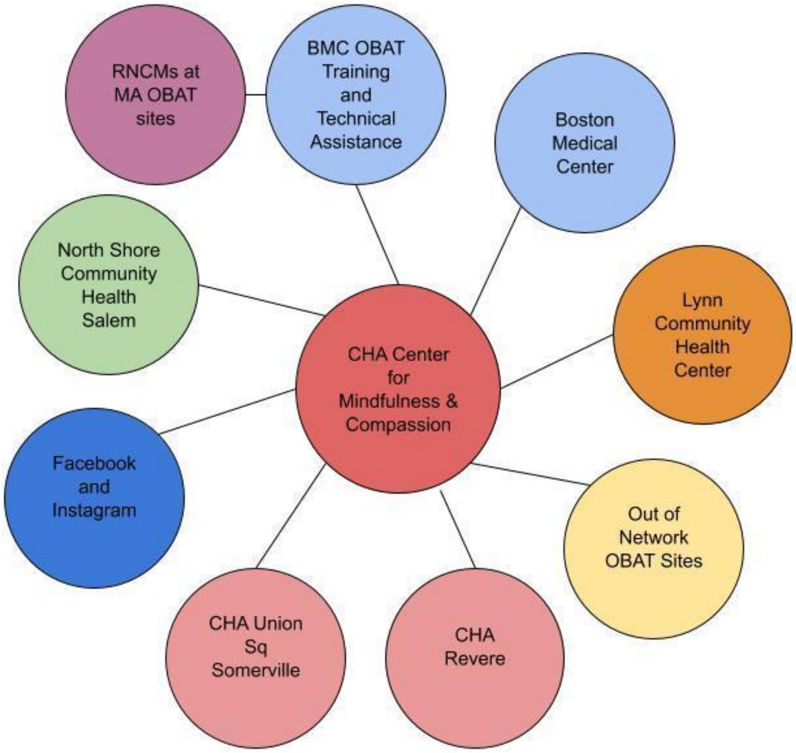

Figure 2. Study Schema

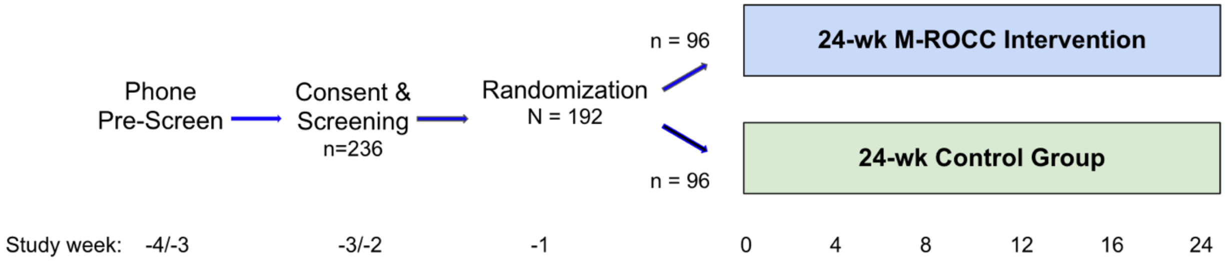

4. SELECTION AND ENROLLMENT OF PARTICIPANTS

4.1 Inclusion Criteria

All of the following are required criterion for inclusion in the study:

1. 18-70 years old
2. Participant lives in Massachusetts, Connecticut, Florida, Maine, New Hampshire, New York, Rhode Island, Texas, Vermont, California, North Carolina, Virginia, Alabama, Arizona, Illinois, or Michigan.
3. Participant is currently prescribed Buprenorphine.
4. Diagnosis of opioid use disorder prescribed a stable dose of buprenorphine (at least 4 weeks)
5. Less than 90 days of abstinence (from non-prescribed opioids or benzodiazepines, cocaine, or alcohol) **OR** OUD with a co-morbid anxiety or stress disorder (as evaluated by Computerized Adaptive Testing for Mental Health [CAT-MH] or PROMIS-ASF $\geq$55).
6. Able to use an electronic device with a videocamera to attend study groups and complete questionnaires.
7. Sufficient English fluency to understand procedures and questionnaires
8. Ability to provide informed consent.

4.2 Exclusion Criteria

Any of the following is regarded as a criterion for exclusion from the study:

1. Active psychosis
2. Bipolar I disorder history or severe level of mania on CAT-MH ($\geq$71)[169]
3. Acute suicidality or self-injurious behavior or severe level of suicidality on CAT-SS ($\geq$71)[170]
4. Cognitive inability as demonstrated by both the inability to complete an informed consent assessment AND complete the Montreal Cognitive Assessment (MOCA-BLIND) <16[171,172] when given chances on two different days
5. Current participation in another experimental research study
6. Previous participation in an 8-week intensive Mindfulness-Based Intervention in past 3 years or participation in the MINDFUL-OBOT pilot study
7. Expected medical hospitalization in next 6 months

8. Expected incarceration in next 6 months
9. Substance use severity requiring likely inpatient treatment in opinion of principal investigator (e.g., severe alcohol withdrawal symptoms, severe benzodiazepine withdrawal symptoms, etc.).
10. Inability to participate in group intervention without disrupting group in opinion of principal investigator.

4.3 Study Enrollment Procedures

Participant Recruitment

We will primarily recruit from 3 non-CHA affiliated sites who have established experience providing OBOT (Boston Medical Center (BMC), Lynn Community Health Center (LCHC), and North Shore Community Health (NSCH)), as well as 2 CHA affiliated sites with an existing partnership with CHA (CHA Revere, CHA Union Square), in addition to up to 50 different primary care sites in Massachusetts associated with the MA OBAT Training and Technical Assistance (TTA) network, OBAT sites in U.S. states approved by the IRB for recruitment, and via Facebook and Instagram advertisements. To ensure a rigorous design, we will refer to the study during recruitment as a "study comparing two group programs designed to help reduce stress, anxiety, and craving during recovery," which would avoid the impact of expectation bias towards or against mindfulness.

Referral process is described below. Participants will be recruited through:

1. A color flyer describing the details of the study, given to site staff at primary care sites throughout U.S. states approved by the IRB for recruitment to distribute to patients.
2. Information about the study will be posted on the CHA Addictions and CHA CMC website after IRB approval of the patient flyer.
3. The MA OBAT Training and Technical Assistance program will send a provider flyer to all nurse care managers working in OBAT across the state of Massachusetts to distribute to prescribers. Providers will receive a provider version of this flyer which will help them identify patients who may be interested and eligible.
4. Patients will be given the study flyer by their providers and nurse care managers. Providers and nurse care managers will not recruit or consent patients to the study, but rather will have the flyer available for any patients prescribed BUP who may be interested. Providers will refer interested patients to the study staff via email (stressreduction@challiance.org) and/or by

completing a google form on the CHA CMC website. Providers will receive verbal consent from their patients to pass along their names, phone number, and email address to study staff so that they may be contacted regarding their interest in the study. Clinicians will be asked to include their email, as well as the optimal method and time to reach the patient they are referring. The referring clinician will only be contacted by the study team regarding enrollment status (i.e., enrolled or not enrolled) once a patient has signed the ICF and consented to this contact. Patients will also be encouraged to contact study staff via email if they are interested in the study.

5. Advertisements will be distributed to potential participants via Facebook and Instagram advertising. All advertising management will be conducted through Facebook, which controls advertisement on both social media platforms. The text and images used in the advertisements will be the same across platforms unless otherwise specified. The research team will not specifically choose individuals or groups to see these advertisements. The ad dissemination process will be completely automated, and Facebook will use a proprietary algorithm to identify users to whom the advertisements will be displayed. Specifically, these advertisements will be targeted to Facebook and Instagram users between the ages of 18-65+ who live in U.S. states approved for recruitment. Facebook does not allow advertisers to specify target ages above 65 and the research team will instead screen out referred participants who are over the age of 70 during the initial phone pre-screening call. Facebook will not share any user information with the research team, and the research team will not be able to identify the specific Facebook and Instagram users to whom the advertisements are displayed. Individual users will have the ability to like and comment on the advertisements and to post the advertisements to their personal pages; however, this would be done at a user's own personal discretion and no incentives will be provided for liking or sharing the Facebook or Instagram ads for this study. Furthermore, to protect confidentiality, our research team will not track or record any information about specific Facebook or Instagram users who may like, comment on, or repost the study advertisements. All Facebook and Instagram advertisements will advertised by Cambridge Health Alliance's official Instagram account, "cambhealthalliance".
6. The recruitment team will not communicate with group moderators, groups, or individuals on Facebook or Instagram. The advertisements will be run through Facebook and the recruitment team will not specifically choose individuals or groups to see the advertisement. The criteria specified during the advertisement creation will be used by Facebook's algorithm to determine who is able to view the ads.

7. The principal investigator will not recruit his own patients into the study, though recruitment coordinators and Co-Is will support patient recruitment at each site.
8. Google advertisements and bulk-mail advertisements to local catchment area will be used if recruitment is slower than expected. All advertisements will require IRB approval prior to use.
9. The study team will utilize contact information in the Helping to Enhance Addiction Recovery Database (HEARD). Only participants from the "Exploring the attitudes and experiences of patients engaged in buprenorphine treatment during the COVID-19 pandemic" study who indicated their interest in future research opportunities will be contacted. The HEARD database contains contact information for individuals with OUD prescribed Buprenorphine who have indicated a willingness to be contacted about future research opportunities. Participants who elect to provide contact information and indicate that they would like to be contacted about future research opportunities will have their contact information (email address and phone number) added to a confidential, password-protected database by a team member who is listed on both study rosters. This database will only contain email addresses and phone numbers, and it will not be connected in any way to other data collected as part of this investigation. Participants from the present Mindful-OBOT R33 study who have indicated a willingness to be contacted about future research opportunities in the phone pre-screening form or the ICF will also have their names, phone numbers and email addresses added to this database. The PI will seek IRB approval for use of this database in any future research. Contact information will be stored for a period of five years or until we receive a request to remove someone's contact information from the database.

Procedures for documentation of reasons for ineligibility and for non-participation of eligible candidates (e.g. Screening Log)

Interested patients will be referred to the study by their medical provider or nurse care manager via email or phone. Referring providers or nurse care managers will be able to describe the study to patients and obtain verbal consent to share patient names and contact information directly with the study team for study coordinators to reach out. Patients may also hear about the study through Facebook, Instagram, google ads or bulk mail ads and will have the option to express interest to the study team through a google form created to collect contact information. Since we will be recruiting patients from different primary care sites across the region, a trained Research Coordinator (RC) will conduct 15-30 minute phone screens with each interested patient to assess their eligibility. Patients will verbally consent to the phone screen and will be read a statement of confidentiality (see phone screen script included in the application). This will protect patients from unnecessarily consenting to and enrolling in the study and completing

screening measures if there is an obvious reason for their ineligibility that emerges through the phone screen. Decisions on inclusion/exclusion will be made by the RC with support from the PI, CMC medical director, and MINDFUL-OBOT clinical director. The participant will also be asked to provide emergency contact information (name and phone number) for their buprenorphine prescriber (in the demographics survey), who the study team will reach out to provide more context surrounding emergencies or potentially life-threatening AEs involving the participant. If the participant meets inclusion and exclusion criteria, then the RC will contact the patient and suggest scheduling an online study screening and consent visit via zoom videoconferencing. If the participant is deemed ineligible to participate during this screen, their reason for ineligibility will be documented and all pre-screening data will be removed from the pre-screening database.

During this visit, the patient will be given a verbal overview of the key information related to the study by the research coordinator, and an opportunity to consent to enrollment via the electronic consent module in REDCap. Once a patient completes the consent process by scoring at least 90% on the online consent assessment and signs the electronic consent form through REDCap, he/she will be referred to as a “participant” in the study. However, if the patient is unable to score 90% or higher on the consent assessment, then the research coordinator will administer the Montreal Cognitive Assessment-Blind (MOCA-BLIND) online through zoom to assess for eligibility (a study comparing the MOCA administered via videoconference vs. in person did not generate significant differences in outcome between the two groups[193]). If they pass the MOCA-BLIND, then they will be allowed to review the consent document again with the research coordinator and complete a new consent assessment. If the participant does NOT pass the consent assessment on the second attempt, then they will no longer be eligible to join the study. If they do not pass the MOCA-BLIND, then the screening visit is over, and they will be able to be assessed once more on another day (since substance use can temporarily increase psychiatric symptoms and impact cognitive performance). On another day the coordinator will meet with the patient via zoom videoconferencing to review the protocol highlights again with the interested patient and offer a second chance to complete the consent assessment and MOCA-BLIND. If the participant has a MOCA-BLIND score below 16 on two separate occasions or scores less than 90% on the consent assessment again, they will not be eligible. If both failures are considered to be potentially due to ongoing substance use, then they may be screened again after 30 days if there has been a clinically significant change in their substance use status based on the judgement of the Principal Investigator (for example, they stopped alcohol use or tapered off the illicit benzodiazepine use, etc.). Once they sign the consent, they will receive a baseline diagnostic assessment using CAT-MH to identify psychiatric diagnoses and risk factors, and a review with a clinician will be required if there is possible psychiatric exclusion. Participants will also complete the PROMIS-ASF and the PhenX Substance Abuse and Addiction Core toolkit.

During this screening process, all eligible participants will need to rate the level of scheduling accessibility of the study groups days and times for both M-ROCC and the control group times being offered. Scheduling acceptability for assigned group will also be used as a covariate in statistical analyses.

If the participant is not excluded based on diagnosis, then they will be scheduled to complete the baseline survey and behavioral measures. If the participant has exclusion criteria on diagnostic assessments or cannot complete baseline measures, then the participant will not be eligible for randomization. Since participants with opioid use disorder often have transient mental health symptoms or intoxication related to recent substance use, participants with transient symptoms related to recent substance use will be able to wait for a re-review of eligibility after 30 days.

5. STUDY INTERVENTIONS

Figure 3. M-ROCC Group Outlines

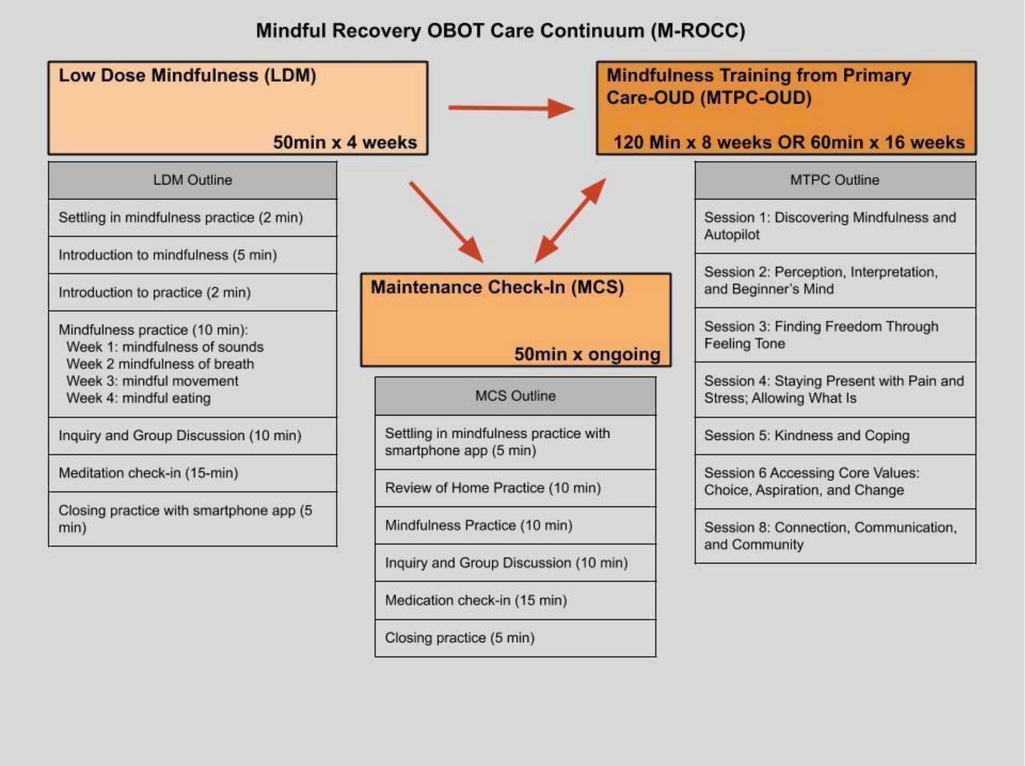

5.1 Interventions, Administration, and Duration

<u>Interventions:</u> *Live-Online M-ROCC, the Mindful Recovery OUD Care Continuum*

While the 8-week MBI standard has been helpful for research reproducibility, it often can lead participants with OUD to feel overwhelmed by practice expectations, unintentionally causing self-judgment and shame, leading to relapse. For others, just as mindfulness begins to click for them at 8 weeks, the program ends. For this reason, M-ROCC is stage-oriented and motivationally-responsive. M-ROCC (Fig.3) meets participants where they are. The introductory low-dose mindfulness group (LDM) is the first stage and it is designed to foster engagement, reduce stress, introduce skills for autonomic regulation, and provide a non-judgmental group environment, exposing participants to mindfulness and self-compassion with a 4-week minimum commitment[194,195]. The LDM group uses rotating short modules in 50-minute sessions for 4-8 weeks with short mindfulness practices, experiential exercises, recordings, and introduction to free online mindfulness resources, with demonstrated feasibility to integrate into addiction treatment[110]. M-ROCC allows people to repeat the 4-week LDM group as needed as they work with the ascending practice dose ladder and become ready for the intensive group, working to build confidence, safety, self-efficacy, and enhance motivation for mindfulness and recovery. After completion of LDM, participants are encouraged to start an intensive group (MTPC-OUD) of 6-15 people. If at least 8 people are ready to transition to an intensive group after 4 weeks then the group will be able to do so. Those who are not ready to transition when the majority of the group transition at either 4 or 8 weeks, can transition to another LDM group at another time if they would like to do so. Participants will join an MTPC-OUD group for 16-weekly 90 minute sessions, which are designed to enhance self-regulation, integrate mindfulness for living well through stress, anxiety, depression, pain and addiction recovery, create an experience of common humanity, and learn from each other's experiences[196]. The 16-week MTPC-OUD group is designed to minimize scheduling difficulties on behalf of both patients and providers, and to increase feasibility in primary care settings where scheduling a longer group may be challenging. Whereas the LDM group is designed to introduce participants to mindfulness practice in an accessible and motivational manner, the MTPC-OUD group is more similar in content to an intensive 8-week MBIs in terms of practice and content. Participants are asked to engage in longer practices (~30 minutes) designed to address more complex topics such as self-compassion, self-management for chronic illnesses, and skills for addressing cognitive patterns of worry and rumination that could otherwise overwhelm novice meditators. After completion of MTPC-OUD, participants can go to a mindfulness maintenance (MCS) group, available in the form of CMC's weekly free live online mindful recovery addiction maintenance community group. This group will run for 60 minutes, and will begin with a longer mindfulness practice followed by a brief inquiry, a brief recovery check-in, and concluding with a shorter mindfulness practice. MCS groups may also be available in the wider community. These groups are flexible in terms of delivery, provided they meet the requirements to be considered an MCS group (mindfulness practice, inquiry and discussion, group check-in). Participants will be given

the option to participate in one of these groups in order to strengthen their practice, however community groups will not be considered to be part of the study or the M-ROCC curriculum. M-ROCC's key focus on autonomy, collaboration and naturalistic selection to different levels based on readiness reduces unnecessary attrition and decreases the likelihood of participants feeling shame or failure if they fail to attain higher levels of practice.

Importantly, due to the impact of COVID-19 all M-ROCC groups will take place online via HIPAA compliant Zoom software. Each M-ROCC group will start with a 15-30 minute live-online check-in period. During this time, participants will be able to meet together in groups of three to discuss their week and experience with mindfulness practices. This brief group is designed to foster interpersonal connection in an online setting where it may otherwise be difficult for participants to interact. Each week we will randomly select 3-6 individuals who will conduct supervised oral fluid toxicology tests during this check-in period with study staff in a private Zoom breakout room. The study team will aim to supervise oral fluid testing among participants once every 2 weeks. If participants do not show up for group but they were randomly selected, then they will be expected to meet within 24 hours with study staff by video to conduct the online screening test.

The Mindful Recovery Opioid Care Continuum (M-ROCC) integrates an ascending practice dose ladder across the 3 group stages. This slow and focused approach to developing home practice differs from standard MBIs which expect static high levels of practice throughout. In a previous pilot of an 8-week MBI group for OUD, participants reported feeling discouraged, overwhelmed, guilty, and that it moved too fast to learn and remember the multiplicity of skills taught each week. More time was needed to develop mindfulness practice confidence, which is important because mindfulness practice dose is associated with the amount of neurophysiologic change people experience during MBIs (e.g., attention/inhibitory control)[197].

The ascending practice dose ladder, which is introduced in the LDM group, provides steady encouragement to increase mindfulness of breathing by an average of 5 minutes of practice per week, building up to 20 minutes a day, providing validation for any practice they are doing, building confidence and self-efficacy. Once the participant begins the MTPC-OUD group they will therefore have an existing mindfulness practice, which will allow them to engage in longer 30-minute practices without being overwhelmed. If the program moves too fast with unrealistic expectations at the outset, then self-stigma, self-criticism, regret, and avoidance can lead to dropout, which has more substantial clinical implications in OUD treatment (relapse, overdose) than in standard MBIs for other diagnoses, where completion rates are often around 50%[49]. In the M-ROCC R21 pilot,

one participant stated the most important part of the LDM group was "going from 5 minutes to 15 minutes a day of practice". **In this way, a stage-oriented continuum of care holds promise to impact OUD outcomes.**

Comparator (Live-Online Control) Design:

This randomized controlled trial will employ an attention- and time-matched live online control group as a standalone comparator intervention. In a similar manner to the M-ROCC check-in group, this weekly control group will offer participants the ability to connect in groups and discuss the challenges that they are facing online with each other. This approach allows for participants to connect in a manner that they would not otherwise be able to, considering that the majority of B/N prescription sites are not offering in-person groups as a result of the COVID-19 pandemic. After completion of the testing and check-in groups, the full group will come together for a 45-60-minute check-in as a group. As an active group comparator, this method will help to isolate mindfulness aspects by controlling for the therapeutic aspects of group and will match the toxicology screening methods across groups as well. Group content will derive from commonly used methods in community substance use disorder programs and recovery groups, including an eclectic curriculum designed to emulate common curricular elements, with representation from commonly implemented evidence-based approaches (Motivational Interviewing, CBT, Community Reinforcement and Twelve Step Facilitation[198]), using engagement and group building processes commonly used in addiction community groups[199]. The manualized group will be standardized across each control group to ensure low variability across cohorts, starting with group check-in and then presentation of a skill and/or discussion of a topic.

<u>Administration</u>

Participants will be enrolled on a rolling basis in preparation for their randomization to either the live-online M-ROCC or live-online control arm. Initially, randomization will only occur once 24 patients are ready for randomization. Following this initial group, every 4 weeks if at least 24 participants have completed baseline measures and are ready to start a group, they will be randomized using randomly selected block sizes of 4, 6 and 8[200] with a 1:1 ratio (24 weeks of live online M-ROCC : 24 weeks of live online control group). Next, after randomization, the participant will be informed about their assignment and given a start week for group sessions (either live online control or live online M-ROCC LDM) and will be given information about the group and its structure with a start date scheduled. We will aim to recruit constantly, with a new M-ROCC and control group beginning every 4-5 weeks.

We will aim for participants to begin attending control or LDM groups as soon as possible after randomization, but at least within 4 weeks. The study timeline will start

with week 1 being the first week the participant is assigned to start the study assigned group. Participants will continue to participate in standard care at each site while awaiting randomization and group placement, as well as throughout their enrollment in the study. For both study arms, participants will verify their presence with the group leader or research coordinator who will take attendance each week, which will be transferred to REDCap by the research coordinator after group.

After completion of the rigorous RCT consisting of 4-8 weeks of low dose mindfulness (LDM), LDM participants will be encouraged to proceed after LDM into the remainder of the M-ROCC program with a naturalistic design to evaluate the choices and outcomes that OUD participants make when engaging in additional mindfulness training (MTPC-OUD) and MCS sessions. After completion of LDM, participants may repeat with multiple cycles of LDM or may enter the next MTPC-OUD group. The availability of MTPC-OUD groups will depend on the number of group participants interested. Intensive mindfulness group-based intervention will be scheduled after 8 participants are willing to transition to intensive, but can only start when there is a critical mass greater than 6 participants to maintain the synergistic container effects important to the mindfulness group approach[176], such as common humanity and learning from each other's experiences.

MTPC-OUD intensive groups will run every 1-2 months and MCS groups will be run continuously in the form of free live online addiction mindful recovery groups. The CMC Free Live Online addiction recovery group led by the M-ROCC clinical director is a live-online group (https://www.chacmc.org/connect) following the MCS curriculum, which can be accessible by Zoom for anyone, and would be available for any study participant who has completed an MTPC-OUD group, or who is not comfortable moving from their LDM group to an MTPC-OUD group.

We anticipate about half of the M-ROCC sample will choose to initiate MTPC-OUD within the first 8 weeks. This is based on our having 9 out of 18 people who enrolled in the LDM in our initial pilot site choose to participate in MTPC-OUD.

M-ROCC participants may join the MCS or the intensive MTPC for the remainder of the study according to their choice. In the pilot study, 2 out of 18 people who started M-ROCC chose to return to a standard group-based opioid treatment (GBOT) level of care and 2 transitioned to individual therapy. Given the risk of overdose in this population, after 4 weeks of LDM, if a participant in M-ROCC chooses to leave the M-ROCC group and return to another level of care (perhaps because of schedule or change in risk level), then we believe it is imperative that we allow them to do that to avoid risk of harm. This is part of the naturalistic design and data on switching will be collected.

The LDM and MTPC group sessions will not be billed to insurance as group psychotherapy or shared medical visits and no-co-pays will be collected. These will be

run as research groups – this will be outlined in the consent form. Participants will be required to remain in their existing BUP treatment program in addition to being randomized to either study arm. MCS groups will be 50 minute groups that participants will be able to join after the study, if the study group ends at 20 weeks for them, or if they decide not to do the live-online intensive MTPC-OUD program. Free MCS groups will be available through the CMC Free Live Online Community Program.

Duration

Participants will complete study procedures for a total of 24 weeks. Participants will be asked to make a live-online group commitment for at least 4 weeks (attending groups once a week), after which they will be encouraged to attend weekly groups for 16 additional weeks. Since group participants will be referred from multiple different sites, the importance is that the requirement is the same across both study arms.

If a participant is in the MTPC-OUD group at week 24, then they will still complete the week 24 assessments within the 2-week completion window even though they have not completed the MTPC-OUD group.

Any assessment battery (specifically online surveys not including oral fluid screens) completed more than 2 weeks after it is expected to be completed by the study timeline would be marked as outside the acceptable assessment window and excluded from primary analyses. Sensitivity analyses for late survey data collected outside this time window will be conducted and if it does not differ than it may be considered for inclusion in analyses.

Alternatives to Participation

Patients receiving buprenorphine at their primary care site can stay in the regular clinical OBOT program at their individual site and opt to not participate in this research study.

5.2 Handling of Study Interventions

The MTPC manual and the Low-Dose Mindfulness (LDM) manual have been tailored specifically for participants with OUD in primary care buprenorphine treatment. Prior to the start of the R33 M-ROCC groups, the manuals will be finalized based on the remaining participant feedback from the R21 phase, and merged into a single adapted, stage-based Mindful Recovery OUD Care Continuum document with an ascending practice dose ladder, which was demonstrated to result in high levels of daily mindfulness practice among participants with MAT in previous trials. These manuals will be further refined for adaptation to a live-online setting. This manual will be sent to NCCIH prior to start of R33 study enrollment.

5.3 Concomitant Interventions

5.3.1 Allowed Interventions

All other non-experimental interventions are allowed as long as they do not conflict with inclusion/exclusion criteria. Many participants are also receiving standard care for their chronic illness and mental health, which may include trials of psychopharmacologic agents or psychotherapy.

5.3.2 Required Interventions

Participants will be required to remain in their regular OBOT program according to standard of care at their referring site. All BUP prescriptions will be tracked and managed by the participant's clinical team. There are no additional required interventions except the mindfulness practice, which will be encouraged in M-ROCC.

5.3.3 Prohibited Interventions

Psychopharmacologic treatments AND diagnoses related to active psychosis, thought disorder, schizophrenia, or schizoaffective disorder as these would indicate that the participant meets one of the exclusion criteria. Psychopharmacologic treatments with atypical antipsychotics and mood stabilizers for the adjunctive treatment of mood disorders or bipolar 2 disorders are NOT prohibited unless there is active psychosis.

5.4 Adherence Assessment

Measurement and Reporting of Participant Accrual, Compliance with Inclusion/Exclusion Criteria:
Review of the rate of participant accrual and compliance with inclusion/exclusion criteria will occur monthly by the data analyst with reports to PI monthly to ensure that a sufficient number of participants are being enrolled. Accrual will be reported twice yearly to the DSMB.

Measurement and Reporting of Participant Adherence to Intervention Protocol:
Participants will be considered adherent to the intervention protocol if they attend at least 3 LDM or control groups. Participants will be considered adherent to the overall M-ROCC intervention if they attend at least 12 MTPC-OUD or MCS groups and were adherent to the LDM group with at least 3 LDM sessions attended. Participants will be

considered adherent to the control intervention if they complete 15/24 control groups. They will be adherent to weekly surveys and eligible for a completion bonus if they complete 75% of these survey sessions and all 2-week time periods have an oral fluid screen. If assigned a study phone, participants must also return this phone to be eligible to receive this bonus.

Measurement and Reporting of Group Leader Adherence to Intervention Manual: Data on adherence to the intervention protocol will be collected weekly by research staff and reviewed monthly by the PI, and twice yearly by the study statistician and DSMB. Adherence of participants will be evaluated by attendance at weekly sessions (Control, LDM, MCS or MTPC-OUD). Adherence for each intervention session will be rated on the following scale: 0 = absence; 1 = completed; 2 = incomplete (with comments). An explanation of study staff efforts to monitor and address participant adherence deficits will be documented and reviewed twice weekly by the senior RC. All sessions will be audio recorded, and 10% of recordings will be reviewed by a trained reviewer rating for adherence, competence, and fidelity, using the MBI:TAC[201].

6. STUDY PROCEDURES

6.1 Schedule of Evaluations:

Table 1a: Schedule of Evaluation from Screen to Wk 8

	Consent & Screening	Baseline		Study Week							
			1	2		3	4	5	6	7	8
**Informed Consent and Screening Session**											
Informed Consent Form	X										
Consent Quiz	X										
PROMIS-ASF	X										X
MOCA-BLIND – if required	X										
CAT-MH	X										
PhenX Addiction Core T1	X										
Demographics Survey	X										
**Baseline Battery**											
Meditation and Mindfulness Experience (SMME)		X									
Adverse Childhood Experiences (ACE)		X									
**Clinical Assessments**											
PROMIS-PISF		X									X
Beck Anxiety Inventory (BAI)		X									X
Oral Fluid Toxicology Results		X					X				X
Weekly Survey			X	X		X	X	X	X	X	X
Pain Catastrophizing Scale (PCS)		X									X
Substance Craving Scale (SUBCS)		X	X	X		X	X	X	X	X	X
**Self-Regulation Battery**											
Experiential Avoidance (BEAQ)		X									X
Difficulty in Emotion Regulation (DERS-16)		X									X
Perceived Stress Scale (PSS)		X									X
Self-Compassion Scale (SCS)		X									X
Interoceptive Awareness (MAIA-2)		X									X
Nonattachment to Self Scale (NTS)		X									X
Self-Critical Rumination Scale (SCRS)		X									X
Experiences Questionnaire Decentering Subscale (EQD)		X									X
**Mechanisms Battery**											
Interpersonal Mindfulness Scale		X									X
Credibility/Expectancy Survey -Stress				X							
Credibility/Expectancy Survey - OUD				X							
Five Facet Mindfulness Questionnaire (FFMQ)		X									X
Group Attitudes Survey											X
**Monitoring surveys**											

Weekly Mindfulness Practice Diary			X	X		X	X	X	X	X	X
Weekly Recovery Skills Diary			X	X		X	X	X	X	X	X
Adverse Events Reporting Form			X	X		X	X	X	X	X	X
Qualitative Interview											
**Duration (min)**	**69**	**77**	**7**	**13**		**7**	**17**	**7**	**7**	**7**	**76**
**Payments for Study Visits ($)**	**20**	**40**	**7**	**12**		**7**	**12**	**7**	**12**	**7**	**52**

Table 1b: Schedule of evaluations weeks 9-24

	**Study Week**																	Duration (min)
	9	10	11	12	13	14	15	16	17	18	19	20	21	22	23	24		
**Informed Consent and Screening Session**																		
Informed Consent Form																		20
Consent Quiz																		10
PROMIS-ASF								X								X		3
MOCA-BLIND – if required																		10
CAT-MH				X												X		12
PhenX Addiction Core T1																		10
Demographics Survey																		4
**Baseline Battery**																		
SMME																		3
Adverse Childhood Experiences (ACE)																		4
**Clinical Assessments**																		
PROMIS-PISF								X								X		3
Beck Anxiety Inventory (BAI)								X								X		5
Oral Fluid Toxicology Results				X		X		X		X		X		X		X		10
Weekly Survey	X	X	X	X	X	X	X	X	X	X	X	X	X	X	X	X		1
Pain Catastrophizing Scale (PCS)								X								X		3
Substance Craving Scale (SUBCS)	X	X	X	X	X	X	X	X	X	X	X	X	X	X	X	X		2
**Self-Regulation Battery**																		
Experiential Avoid (BEAQ)								X								X		3
Emotion Regulation (DERS-16)								X								X		5
Perceived Stress (PSS)								X								X		3
Self-Compassion (SCS)								X								X		5
Interoceptive Awareness (MAIA-2)								X								X		5
Nonattachment to Self Scale (NTS)								X								X		2
Self-Critical Rumination Scale (SCRS)								X								X		3
Experiences Questionnaire Decentering Subscale (EQD)								X								X		5
**Mechanisms Battery**																		
Interpersonal Mindfulness Scale								X								X		4
Credibility/Expectancy Survey - Stress																		3
Credibility/Expectancy Survey - OUD																		3
Five Facet Mindfulness Questionnaire (FFMQ)								X								X		6
Group attitudes								X								X		2
**Monitoring surveys**																		
Weekly Mindfulness Practice Diary	X	X	X	X	X	X	X	X	X	X	X	X	X	X	X	X		2
Weekly Recovery Skills Diary	X	X	X	X	X	X	X	X	X	X	X	X	X	X	X	X		2

Adverse Events Form	X	X	X	X	X	X	X	X	X	X	X	X	X	X	X	X		2
Qualitative Interview																X		60
**Visit Duration (min)**	**7**	**7**	**7**	**31**	**7**	**17**	**7**	**76**	**7**	**17**	**7**	**17**	**7**	**17**	**7**	**98**		
**Payments for Visits ($)**	**7**	**12**	**7**	**12**	**7**	**12**	**7**	**52**	**7**	**12**	**7**	**12**	**7**	**12**	**7**	**102**		

6.2 Description of Evaluations

6.2.1 Screening Evaluation

Pre-Screening Process

In order to be eligible to participate in the study, all patients must have an active buprenorphine prescription from a provider and must reside in a state approved by the IRB for recruitment. Study participants will be referred by their provider, by the OBOT nurse care manager, or they will be self-referred. Referring clinicians will be able to collect verbal consent to share patients' name, email and phone number with study RCs who can reach out to the patient to describe the study in more detail. Referring clinicians will also be able to provide study team contact information to the participant so that they will be able to contact the Research Coordinator (RC) directly. The providers will not be members of the study staff, they are members of the clinical staff who will work with study staff to help make the study available to their patients should their patients find it a good fit.

Following the participant's referral to the study, the RC will conduct a brief phone screen, with the outcomes recorded in a REDCap survey, to determine the participants eligibility. If the participant meets inclusion and exclusion criteria based on the phone screen, then the RC will contact the patient and suggest enrollment in the study followed by study screening. If the participant is deemed ineligible to participate during this screen, their reason for ineligibility will be documented and all pre-screening data will be removed from the pre-screening database.

For patients for whom there are clinical eligibility questions, eligibility will be reviewed by MINDFUL-OBOT Clinical Director, MINDFUL-OBOT PI, or CMC Medical Director prior to the screening visit. The research coordinator will review information provided by the participant during their phone screen to confirm evidence of inclusion criteria. If the patient appears eligible from the brief review, then the Research Coordinator will call all patients with a referral to schedule a screening visit. Eligibility and phone screen information will be stored in a secure REDCap database.

6.2.2 Enrollment, Baseline, and/or Randomization

Study enrollment is defined as the date when the informed consent is completed. Once the participant is determined eligible from screening, they will complete their baseline measures. Once baseline measures are completed if they are still eligible, then the participant is ready for randomization, which is described below.

Consenting Procedure

Electronic informed consent will be obtained from each participant at entry into the study via a secure REDCap server hosted at CHA. Informed consent is obtained by the following process:

<u>Consent for Participants for study:</u>

Low-Risk: The informed consent document will include a full description of the study procedures and associated risks. Because these interventions are well-established with more than 1000 studies demonstrating the safety and efficacy of Mindfulness-based interventions[203], the groups represent a low-risk experience.

Consent procedure: Strict confidentiality during the consenting procedure will be maintained through the use of REDCap for document consent, and the study staff will be trained on maintaining confidentiality during the consent process. The study staff will call or video chat with the participant to guide them through the consenting process. The research coordinator will first describe the measures taken to keep the consent session confidential and safe for the participant, and then will describe the key information of the study verbally, using a study schema diagram designed for easy patient comprehension, and will then give the patient time to review the consent form on their own. The patient will be given a chance to ask questions and express any concerns about the study.

Prior to study enrollment, during the consent process, the participant will be asked to provide their personal information (name, date of birth, address, phone number). No study-specific procedures or investigations will be performed before the patient has signed and dated the Informed Consent Form. The electronically signed and dated informed consent forms will be stored in a secure REDCap server hosted by CHA. The RC will also sign the consent form, indicating that they were present during the consent process. The participant will be emailed a copy of their signed and dated consent form for their own records. Once participant consent has been obtained, the RC will ship a package to the participant containing Oral Fluid Toxicology testing kits.

Consent assessment: The participant will also be asked to complete an Informed Consent Assessment via REDCap to ensure comprehension of the study procedures, rights of participants, as well as understanding of the risks and benefits of the study. The participant will not be able to sign the document without having at least 90% correct answers on the consent assessment.

Participants will be given the opportunity to review the study procedures with a trained study staff member if they fail the consent assessment. Participants will also be given an opportunity to correct their answers on the consent assessment. If they can't pass the assessment with at least 90% completely correct answers after reviewing it and the protocol with the coordinator, then they will meet individually with the Research Coordinator or other approved study personnel to be evaluated for the presence of cognitive deficits disrupting their ability to complete study tasks, including completing the MOCA-BLIND[204] to evaluate for cognitive deficits that may prevent them from comprehending the study intervention and study assessments. If participants do not obtain a score of 16 or higher on the MOCA-BLIND, the session will end and they will be permitted to reschedule to take the MOCA-BLIND once more on a different day. If they do not obtain a score of 16 or higher on the second day, they will not be permitted to join the study. Since participants with opioid use disorder often have transient mental health symptoms or intoxication related to recent substance use, participants with transient symptoms related to recent substance use may be able to wait for a re-review of eligibility after 30 days based on the judgement of the Principal Investigator.

Participants with at least one score of 90% or higher on the Informed Consent assessment AND a score of 16 or higher on the MOCA-BLIND within up to two screening visits will be eligible for participation in the study. Once the participant demonstrates understanding of the study and agrees to participate in the study, the consent will be signed in the presence of the research coordinator or another trained member of the study team staff. After completion, participants will be given an additional copy of the signed informed consent form for their own records.

Consented patients will be assigned a numeric study number based on their order of enrollment and study acrostic which is based on letters (to allow for cross-referencing and prevent mistaken confusion among participants if a digit is incorrect), and will complete the screening and baseline assessment battery, which will take place after informed consent.

Screening Assessments (Consent and Screening Visit)

After participant consent has been obtained, the research coordinator will provide the participant with a secure REDCap link to the PROMIS-ASF, and PhenX toolkit surveys, as well as a secure link to a screening CAT-MH survey, to evaluate for eligibility prior to proceeding with baseline assessments. The RC will also supervise an oral fluid test with the participant to assess for substance use. If the screening process suggests that the participant meets exclusion criteria or brings up other questions about eligibility, then the RC will contact the PI or clinical director to review the information and decide prior to proceeding to baseline assessments. If a participant's PROMIS-ASF T score is greater than 55 (Raw > 16), then the participant will be determined to have an anxiety disorder even if it was not previously diagnosed clinically.

Since participants with opioid use disorder often have transient mental health symptoms or intoxication related to recent substance use, participants with transient symptoms related to recent substance use may be able to wait for a re-review of eligibility after 30 days.

During this screening visit, all eligible participants will be asked to fill out a Scheduling Acceptability form to rate the level of acceptability of the available study group day and time for both M-ROCC and control group times at the site, asking on a 0-10 scale how much they will be able to attend the group times.

Baseline Assessments (Baseline Visit)

<u>Baseline Survey Session and Computer Tasks Visit:</u>
Eligible participants will complete baseline surveys through a link to the secure REDCap database. They will receive a password protected survey link through an email from the study team.

All participants will be shipped a package containing sponge-based testing kits for Oral Fluid Toxicology testing. Oral fluid toxicology tests will be conducted via videochat with a research coordinator to ensure proper collection. The participant will hold the results panel of the test up to their camera for the research coordinator to screenshot using the HIPAA compliant screenshotting tool "Droplr". These screenshots will be stored in Droplr's secure cloud server before being transferred to the secure CHA Google Drive. Participant faces and other PHI will not be included in the screenshot.

The Baseline Survey Session is expected to take approximately 1.5 hours. Please refer to schedule of evaluations (Section 6.1) for an exact list of assessments used in this session.

Randomization

We will randomize participants in a 1:1 ratio of M-ROCC arm to control arm. We will randomize participants in randomly selected blocks of 4, 6, or 8 in a 1:1 ratio.

Randomization will be conducted as follows:

1) Participants who complete baseline measures and have a scheduling acceptability form will be entered into the randomization database.
2) Upon final review of eligibility, eligible participants will be marked as ready for randomization in the study database, which will alert the data analyst by automated email generated by REDCap.
3) The data analyst will randomize participants who are ready using a randomly selected block size (4, 6 or 8) (1:M-ROCC; 1:Control) and will enter the randomization status into the Study Status spreadsheet which the research coordinators will have access to and is kept secure within the CHA G Suite system.
4) The research coordinator will call the participant by phone to let them know the date/time that they can start LDM/Control.

Participant De-identification, Randomization, Allotment, and Blinding:

Upon consenting to the study, each participant will be assigned a study number by the research coordinator. Randomization will occur using the assigned study number, which the Data Analyst will use to enter each participant into the randomization spreadsheet. The Data Analyst will be blinded to participant identity prior to and during randomization. The research coordinator will not have access to the randomization spreadsheet.

6.2.3 Blinding

See Table 2 for blinding status for study staff. Participants will not be blinded to their assigned study arm (M-ROCC vs. control), nor will the research coordinators, data analyst, or methodologists. The PI will be blinded to assigned study arm when reviewing data quality, adverse events, protocol violations, etc.

Table 2. Blinding Status of Study Staff

Study Role	Blinding Status	Time of unblinding

PI	Blinded	When all primary outcome data have been collected and when database is locked
Data Analyst (Lydia Smith, Gareth Parry, Javier Barria)	Not Blinded	Will prepare reports for DSMB with unblinded data, but will not have contact with participants.
Methodologists (Creedon/Cook)	Not Blinded	Will review reports for DSMB with unblinded data, but will not have contact with participants.
RCs	Not blinded	
Clinical/Medical Directors	Not blinded	
Participants	Not blinded	

6.2.4 Follow-up Visits

Overview of Study Visits:

All study visits will be conducted via Zoom video and password protected REDCap surveys rather than in person. Study visits are outlined below:

- Consent and Screening Session (1 hours)
- Baseline Assessment Session (1.5 hours)
- 8-week visit assessment (1 hour)
- 16-week visit assessment (1 hour)
- 24-week visit assessment (1 hour)
- Oral fluid toxicology screens will be conducted at least once every two weeks during the study and will be entered into REDCap by study staff
- Weekly monitoring surveys will be collected monitoring substance use and Weekly Mindfulness Practice Diary

Participant Communication
Study staff will conduct outreach phone calls every two weeks during the 6-month study. If a participant does not respond to the phone call and has not been responsive within the past two weeks, study staff will reach out using e-mail, text message, or private message via Facebook or Instagram. Phone number, e-mail address, social media handle, and the names and contact for three close contacts will be recorded in the demographics survey outlined in the participant consent form. We will also collect the name and phone number of the patient's buprenorphine prescriber in the

demographics survey. The prescriber would only be contacted in case of emergency or potentially life-threatening adverse event relating to the patient.

According to Mitchell *et al*, sending private messages via text or Facebook was a more effective way of communication with SUD participants than leaving voicemails[204]. Only private, direct messages will be sent to participants through private social media messaging. The voicemail, text, email, and social media outreach attempts will be limited to scheduling and general content and will not include any mention of the research study or the nature of the study. Study participants will be contacted through the "Stress Reduction Study" Facebook and Instagram accounts.

Only secure WiFi will be used to communicate with participants. If a study staff member's home WiFi is not password protected, they will be required to use a study phone. Only a CHA IT approved secure study cell phone and computer, both password-secured, will be used to communicate with participants. All communication will be logged in a G-suite secure study call log, and messages will be deleted from the study cell phone or social media site once logged in the call log.

6.2.5 Completion/Final Evaluation

Final primary outcome/endpoint is number of biochemically confirmed oral fluid toxicology opioid negative abstinent periods (two-week periods of opioid abstinence during weeks 13-24 of the study defined as the number of biochemically confirmed illicit opioid negative abstinent periods (defined by negative oral fluid tests (negative for opiate, oxycodone, fentanyl, methadone) AND no self-reported illicit opioid use) during weeks 13-24 of study.

Final study session is week 24 and all the evaluation measures and assessment batteries that happen within two weeks of that day. Please refer to schedule of evaluations for an exact list of assessments used in this session.

<u>Participant Reimbursement:</u>

Participants will be paid using either anonymous, retail, online reward cards, OR anonymous, HITECH safe reloadable prepaid cards. Specifically, these online reward cards would be provided through a company like Tango (https://www.tangocard.com), a HIPAA compliant rewards service, or Comdata, a CHA approved reloadable gift card vendor. Use of Tango will require the input of

participant email. Use of anonymous, retail, non-reloadable prepaid cards will not require any participant information to be recorded.

Participants will also be rewarded with a completion bonus for an oral fluid screen completion rate of at least 90%, completion of baseline, 8-week, 16-week, and 24-week assessments, and completion of more than 75% of weekly surveys. If assigned a study phone, the participant must also return this phone to be eligible to receive this bonus. Participants will receive an email notification for each weekly gift card they receive.

For completing baseline, 8-, 16-, and 24-week survey batteries within 48 hours of their assignment, participants will be rewarded with $5.

Payment will be given as follows:

- $20 at the Screening/Consent visit for completion of screening
- Up to $45 at Baseline Visit for completion of baseline visit
- $5 per group attended ($120 total)
- $5 per bi-weekly period in which all assigned weekly surveys and oral-fluid toxicology screens are complete ($60 total)
- Up to $45 at 8 Week Assessment
- Up to $45 at 16 Week Assessment
- Up to $45 at 24 Week Assessment
- Up to $48 for 24-week completion bonus if participants complete at least 90% of their randomly assigned oral fluid screens weeks AND complete 75% of weekly surveys between weeks 1 and 24. If assigned a study phone, the participant must also return this phone to be eligible to receive this bonus. This bonus could range from $36 to $48 depending on how many weekly surveys are completed ($2 for each weekly survey completed).
- $50 for completing an optional M-ROCC or control completer qualitative interview (available for first 30 completers).
- **Up to $478 total**

Study Payments will be given the following times:
Payment 1: $20 for Screening/Consent visit
Payment 2: Up to $45 at Baseline survey/computer visit

Payment 3: Up to $45 at 8 Week Assessment
Payment 4: Up to $45 at 16 Week Assessment
Payment 5: Up to $143 at 24 Week Assessment
Weekly Payments: Up to $180 spread out across 24-weeks. Participants will be able to choose whether they receive these payments each time they are earned, or at the end of each large survey battery.

6.2.6 Qualitative Interviews

We will conduct a minimum of 12 and a maximum of 30 qualitative interviews of M-ROCC or control group completers, defined as having attended at least 3 LDM sessions and 12 MTPC-OUD/MCS sessions or 15 control sessions of a possible total of 24 sessions. Interviews will be conducted with a minimum of 6 and a maximum of 15 participants from each study arm. Interviews will last between 30 and 60 minutes. All completers will be invited to interview until thematic saturation is reached. Interviews will be conducted on the HIPAA-compliant videoconferencing technology platform Zoom. Research staff with qualitative interviewing experience will be trained to conduct productive post-study interviews, oversee preparation of transcripts and ensure compatibility for it to be entered into the software program ATLAS.ti version 8.4.4 to enable comparison across qualitative data sources.

The M-ROCC qualitative interviews will be structured to identify:

1) Themes regarding online mindfulness delivery.
2) Challenges of online group delivery
3) Strengths of online group
4) Subjective experience of the online supervised oral fluid testing process
5) Benefits of group-based approach to treatment, hypothesizing that reasons for mindfulness group retention will also include mindfulness practice benefits, body awareness, emotion regulation, acceptance of medication, and values clarification.

The control group qualitative interviews will be structured to identify:

1) Themes regarding online GBOT delivery, such as shared identity, accountability, and supportive community.
2) Challenges of online group delivery
3) Strengths of online group
4) Subjective experience of the online supervised oral fluid testing process.
5) Benefits of group-based approach to treatment, hypothesizing that group-based treatment creates an atmosphere of mutual identification and acceptance in which participants gain support and inspiration through positive role modeling.

Analysis of the qualitative data obtained through these interviews will begin with transcription. The text output from the qualitative interviews will be generated using Transcription Star transcription services (https://www.transcriptionstar.com/), an online service provider that specializes in HIPAA compliant audio transcription. To protect human subjects' privacy, only the audio (mp3) files from qualitative interviews will be sent to Transcription Star. The study staff will only record and download interview data using a desktop computer at the CMC office in order to ensure that the interview files are securely maintained. Files will be downloaded to the secure CHA server before being de-identified and uploaded to the secure G-Drive. Study staff will anonymize the mp3 files (using Audacity) by removing any identifiers mentioned during the interview. Audacity has been previously approved by the CHA IT department, and only saves files locally. The qualitative interviews will be converted from video mp4 format to audio mp3 format using Zoom HIPAA compliant conversion software. Audio recordings in mp3 format will be uploaded to transcriptionstar.com and will be securely stored and transmitted using HTTPS and Transport Layer Security (TLS) 1.2 encryption, which is the highest level of security available. Once the transcriptions are complete, the text of the transcripts will be checked against the digital recordings and errors will be corrected. The transcripts will then be prepared as rich text format files for compatibility with the computer software, ATLAS.ti, version 8.4.4[207]. In addition, other data sources will also be entered by research staff into ATLAS.ti to enable comparison across qualitative data sources.

The transcriptions of the interviews will be coded using two approaches simultaneously: 1) coding using revised a priori codes based on the themes described above; and 2) open coding to identify new concepts found in the data[178]. Trustworthiness of the data will be determined through investigator meetings[180], the application of analytic reflexivity in the interpretive process[180], and constant comparison of emerging themes[181]. To refine themes, the team will further analyze all excerpts within a theme into sub-categories as well as define and name themes. Finally, qualitative themes and results in the primary care implementation pilot will be compared.

7. SAFETY ASSESSMENTS

AEs will be systematically assessed at each assessment visit (weekly surveys, baseline, 8, 16, 24). In addition, group leaders and research coordinators will be trained to identify and report any adverse events that may occur or are reported during weekly live-online group visits. All AEs will be reviewed weekly by the Senior RC and the MINDFUL-OBOT Clinical Director, and monthly by the PI, and SAEs will be reviewed by PI within 24 hours. Please see adverse events section (7.3) below.

7.1 Specification of Safety Parameters

Participants are assessed regularly for AEs, hospitalization, and changes in medication.

7.2 Methods and Timing for Assessing, Recording, and Analyzing Safety Parameters

7.2.1 Expected Risks

1. **Worsening of underlying illness**

   People with OUD are at high risk of worsening of the underlying disorder, which can lead to opioid overdose, intoxication, life-threatening accidents, inpatient hospitalization for detoxification or stabilization, and death. Given the high rate of relapse and overdose death both in this population of patients with OUD, inpatient hospitalization for opioid use disorder and overdose death are both risks that are expected to occur to the study but will be unlikely to be related to the protocol. Co-morbid psychiatric disorders are common, especially PTSD, MDD, Bipolar Disorder, Anxiety disorders, and OCD. Worsening of underlying mental illness may also occur, which would be an expected adverse event. Suicidality is a common symptom in this population and suicidal ideation is an expected symptom as a sign of worsening of underlying mental illness or opioid use disorder. Non-life threatening suicide attempts associated with an overdose, or any non-life threatening, self-harm among patients with prior suicidality, self-harm, past suicide attempts, or other risk factors would be an expected risk of the study. However, if a participant without previous suicidality, previous self-harm, or other risk factors were to attempt to commit suicide or physically harm themselves, then this would be unexpected. Life threatening suicide attempts are an unexpected events. Medical events related to injection, inhalation or other methods of administration of opioids and other drugs of abuse can also happen and would be expected risks in this population.

2. **Time spent learning mindfulness techniques**
   Participants will be invited to practice mindfulness skills and techniques at home, which may involve negotiating with those at home to create time and space for practice. This will not be required of them.

For #3 and #4 below, research coordinators, group leaders, and the PI will be trained to respond to escalating anxiety, psychosis, or any other worsening psychiatric symptoms. They will be trained to alert the buprenorphine prescriber or their delegate (e.g. nurse care manager) by phone/page or electronic medical record (within participating healthcare systems) within 24 hours of becoming aware of the escalating symptoms, and to contact the CMC Medical Director or Principal Investigator by phone or page as soon as possible and at most within 24 hours of alerting the existing care provider (s) to review the case. The care team and study staff will work together to determine the appropriate referral, including referral to outpatient mental health and/or local Emergency Department if necessary.

3. **Increased anxiety due to difficulties in mental training program**

   Mindfulness practices can sometimes cause anxiety for those who practice. Support from the Group Leader and the group can help participants recognize that this anxiety is part of the process and can help people learn to manage these feelings. If meditation and mental exercises are causing a worsening of anxiety for participants, they will be encouraged within groups to change their approach to the practice. Research coordinators will assess for worsening symptoms every two weeks during the engagement call adverse events report, and also during each live-online group session. If the participant(s) are unable to find a way to practice without eliciting an increase in symptoms, then they may be asked to stop practicing and to meet with either the M-ROCC Clinical Director, the CMC Medical Director, or the Principal Investigator for an evaluation. The participant may ultimately be terminated from the study if they are experiencing an adverse reaction. These participants may continue to receive other treatments at their primary care site as part of their ongoing treatment plan.

4. **In some very rare cases, meditation practice can lead to a dissociative state or to psychosis[208].** This is more likely in participants with current conditions of or a predisposition to psychosis. Participants experiencing active psychosis are excluded from participating in this study. If a participant begins to experience these feelings during his or her time in the study, then the study staff and care team will work together to alert the appropriate care team providers and study investigators immediately. The participant may be asked to stop practicing and to meet with either the CMC Medical Director or the Principal Investigator for an evaluation in person or via videoconference while a member of the study staff is onsite with the person. Videoconferencing will occur through the HIPAA compliant and encrypted communications platform zoom. The participant may

ultimately be terminated from the study if he or she is experiencing an adverse reaction. These participants may continue to receive other treatments at their primary care site as part of their ongoing treatment plan. If the participant experiences a dissociative state or psychosis during a group session, the research coordinator and/or group leader will ask the participant to meet with them individually in an online breakout room, while the other/group leader continues on with the session and will contact the appropriate care provider(s).

5. **Physical discomfort due to gentle movement aspect of training.** The training will involve gentle movements and stretching. Some participants could experience physical discomfort during this aspect of training. If a participant feels discomfort, he or she will be encouraged to engage in a less straining manner. If discomfort continues, then the participant will be asked to sit out of this brief element of training and discuss the areas of discomfort with their PCP before return to practice of physical postures. Severe discomfort is not expected, but could possibly occur. In this case, the participant will be treated for this pain, and encouraged to return to practice stationary forms of mindfulness. If a participant gets hurt or get sick as a direct result of being in this study, emergency treatment will be given to them. All needed emergency care is available to participants, just as it is to the general public. Cambridge Health Alliance has not set aside any money to pay for a research-related injury or illness.

6. **Feelings of embarrassment or anxiety when asked personal survey question.** Some survey questions are of a sensitive or personal nature and may cause the participant to become upset. In some rare cases, participants may require mental health support upon feeling upset by the survey questions. A participant's referring clinician will be contacted if the participant needs additional mental health support. The PI, M-ROCC Clinical Director, and CMC medical director will remain available to field questions from participants about their experience with mindfulness and any adverse experiences. The clinical director will help to monitor participants and work with the referring clinician to coordinate behavioral health care for participants.

7. Monitoring may lead to anxiety and difficulty with completion of interviews may lead to feelings of guilt or shame. Staff will specifically be trained not to shame anyone about missed sessions, but instead focus on what they can do to complete the next ones.

8. Despite efforts to prevent data breaches, any use of electronic devices and internet data transmission can result in a **breach of confidentiality**.

9. Group members will be asked to keep what others share confidential, but they may not. As a result, **some private information may be disclosed** by other group members.

7.2.2 Expected Benefits

1. Participants may learn about others who have similar problems as they do, helping them feel less alone
2. Participants may feel increased accountability in their recovery by being in group
3. Participants may have less of a need for symptom-relieving medication like benzodiazepines and opioids.
4. Participants may find that they smoke fewer cigarettes and drink less alcohol.
5. Participants can learn skills for controlling behavior and improved well-being.
6. Participants may feel less depression, anxiety, stress, and pain.
7. Participants may benefit from the support of group-based intervention.
8. Participants may feel more joy and gratitude.

7.3 Adverse Events and Serious Adverse Events

Following NCCIH request, a detailed Data and Safety Monitoring Plan (DSMP) was designed and a DSMB assigned. Please see appendix at the end of this document. Adverse event reporting guidelines are detailed in the DSMP and copied below.

<u>Definition:</u>

In this study we will use the FDA definition of adverse events (AEs): "any untoward medical occurrence that may present itself during treatment or administration with a pharmaceutical product [or group-based treatment intervention], and which may or may not have a causal relationship with the treatment." Serious adverse events for this trial will be defined as any AE temporally associated with the participants' involvement in research that meets any of the following criteria:

- Results in death;
- Is life-threatening (i.e., any suicide attempt requiring emergency department or inpatient care will be a SAE, but parasuicidal gestures or self-harm that is not life-threatening or requiring immediate medical care will be an AE, but NOT a SAE; any opioid overdose requiring Naloxone reversal will be a SAE);

- Inpatient hospitalization or prolongation of existing hospitalization (other than admissions for substance use disorder, which will be classified as an AE, but this alone is not sufficient to classify as an SAE since this may be for detoxification or as a beneficial step towards recovery);
- Results in congenital anomaly/birth defect;
- Results in a persistent or significant disability/incapacity or permanent incapacity or substantial disruption to conduct of normal life functions;
- Based upon appropriate medical judgement may jeopardize the participant's health and may require medical or surgical intervention to prevent one of the outcomes listed in this definition.

<u>Classification of AE Severity:</u>

AEs will be labeled according to severity, which is based on their impact on the participants. An AE will be termed "mild" if it does not have a major impact on the participant, "moderate" if it causes the participant some minor inconvenience, and "severe" if it causes a substantial disruption to the participant's well-being. Of note, a severe AE and a serious adverse event (SAE) are distinct terms. A participant could experience a severe AE that does not meet the above-listed definition of an SAE; alternatively, a participant could experience a moderate AE that meets the SAE definition.

<u>AE Attribution Scale:</u>

AEs will be categorized according to the likelihood that they are related to the study intervention. Specifically, they will be labeled unrelated, possibly related, or definitely related to the study intervention.

7.4 Reporting Procedures

This study will comply with the reporting requirements from the Cambridge Health Alliance IRB. The PI will report to the CHA IRB, DSMB, and NCCIH any of the following *unanticipated problems* and *adverse events* that occur 1) during the conduct of the study, 2) after study completion, or 3) after participant withdrawal or completion:

1. *Internal adverse events* that are *unexpected*, <u>and</u> related or *possibly related to the research* and that indicate there are new or increased risks to participants;
2. *External adverse events* that are *serious*, *unexpected*, <u>and</u> related or *possibly related to the research* and that indicate there are new or increased risks to

participants that require some action (e.g., modification of the protocol, consent process, or informing participants);

3. *Deviation* from the approved research protocol or plan without IRB approval in order to eliminate apparent immediate hazard to participants or harm to others;
4. *Deviation* from the approved research protocol or plan that placed participants or others at an increased risk of harm regardless of whether there was actual harm to participants or others;
5. Any event that requires prompt reporting according to the research protocol or investigational plan or the sponsor;
6. Breach of confidentiality or violation of HIPAA
7. Procedural error regardless of whether participants experienced any harm;
8. Interim analysis, safety monitoring report, publication in a peer-reviewed journal, or other finding that indicates that there are new or increased risks to participants or others or that participants are less likely to receive any direct benefits from the research;
9. Complaint by/on behalf of a research participant that indicates that the rights, welfare, or safety of the participant have been adversely affected or that cannot be resolved by the investigator;
10. Incarceration of a research participant during participation in this study (which is not currently approved for involvement of *prisoners* as participants);
11. *Noncompliance* with applicable regulations or requirements or determinations of the IRB identified by the research team or others that indicates that the rights, welfare, or safety of participants have been adversely affected;
12. *Suspension* or *termination* of the research, in whole or in part, based on information that indicates that the research places participants at an increased risk of harm than previously known or recognized;
13. Suspension or disqualification of an investigator by the sponsor, or others;
14. *Scientific misconduct*; or
15. Any other problem that indicates that the research places participants or others at an increased risk of harm or otherwise adversely affect the rights, welfare or safety of participants or others.

Procedures for collecting and reporting unanticipated problems:

All unanticipated problems (including AEs) will be collected by the PI or other study staff on an AE Tracking Log form, at the following time points: at study assessment time points at baseline and weeks 8, 16, 24, in each weekly survey, and additionally on an ad-hoc basis.

Reports of *unanticipated problems involving risks to participants or others* will be submitted to the IRB, DSMB and NCCIH within 5 working days of the date the investigator first becomes aware of the problem.

Reporting Unanticipated Problems that are Adverse Events:

Any unanticipated untoward or unfavorable medical occurrence, including abnormal sign, symptom, or disease, that indicates that the research places participants at increased risk of physical or psychological harm than previously known or recognized will be submitted as an AE to the IRB, DSMB, and NCCIH. The PI will provide the following information in the report:

1. a detailed description of the adverse event;
2. the basis for determining that the event is unexpected in nature, severity, or frequency;
3. the basis for determining that the event is related or possibly related to the research procedures;
4. the basis for determining that the research places participants at an increased risk of harm (i.e., a serious adverse event); and
5. whether any changes to the research or other corrective actions are warranted.

Reporting Unanticipated Problems that are not Adverse Events:

All other unanticipated problems incidents, experiences, information, outcomes, or other problems that indicate that the research places participants at an increased risk of physical, psychological, economic, legal, or social harm than was previously known or recognized will be submitted as an "Other Event" to the IRB, DSMB, and NCCIH within 5 business days. The investigator will provide the following information in the report:

1. a detailed description of the unanticipated problem;
2. the basis for determining that the problem is unexpected;
3. the basis for determining that the problem indicates that the research places participants at an increased risk of harm; and
4. whether any changes to the research or other corrective action are warranted.

SAE Reporting:

SAEs that are unexpected, serious, and possibly related to the study intervention will be reported to the IRB, DSMB, and NCCIH in accordance with requirements.

Any SAE, whether related to study intervention or not, will be reported to the IRB and the DSMB within 5 business days. The initial SAE report will be followed by submission of a completed SAE report. SAEs that are unanticipated, serious, and possibly related to the study intervention will be reported to the NCCIH within 5 business days. Anticipated or unrelated SAEs will be handled in a less urgent manner but will be reported to the IRB in accordance with their requirements. A summary of the SAEs that occurred during the previous year will be included in the annual progress report to NCCIH. In the annual AE summary, the DSMB Report will state that they have reviewed all AE and SAE reports.

7.5 Follow-up for Adverse Events

Communication of Adverse Events:

The PI and the study Clinical Director will be notified of all adverse events that occur within 48 hours of it being reported to the study team. The PI will then decide what appropriate actions to take in order to ensure participant safety. These include encouraging the participant to contact their PCP or mental health provider; contacting the participant's prescriber; or contacting the participant by phone or email to obtain updates on the adverse event. If the PI determines that a member of the participants clinical team should be contacted to provide context surrounding the potentially life-threatening AE, the study team will use the emergency contact information provided in the demographics form by the participant. In some cases, the participant may have already contacted their relevant providers, and/or the participant has already improved in which case no action is necessary.

Once the necessary steps to ensure participant safety have been enacted, the CMC medical director and/or PI will discuss what actions need to be taken with regard to the study: discontinuing the participant from the intervention temporarily until conditions have improved; discontinuing from the intervention permanently but keeping them in the study; or having them drop out of the study entirely.

7.6 Safety Monitoring

The PI and data analyst will review with the research coordinators data collection, data completeness and accuracy, as well as protocol compliance on a monthly basis. Study progress and safety will be reviewed monthly (and more frequently if needed). Progress reports, including participant recruitment, retention/attrition, and AEs, will be provided to the DSMB every 6 months (see DSMP for details).

Possible Risk of Suicide:

During screening, study applicants will undergo a battery of psychological tests, including the CAT-MH, to determine their eligibility and safety of their participation in this study. Special attention will be placed on history of homicide or suicide attempts and the presence of any current imminent plan. Applicants who are actively suicidal will not receive the study interventions and will be evaluated by the PI to determine whether study participation is appropriate.

If imminent risk of suicide or danger to self or others is evidenced during a screening evaluation, then the participant will be excluded from participation and will be referred to the appropriate level of care as soon as possible. While maintaining contact with the participant, the study team member will contact the National Suicide Prevention Lifeline (800-273-8255) via the CHA HIPAA compliant Google meet application. The PI will be responsible for overseeing risk assessment during screening and study visits. Study PI, Zev Schuman-Olivier, MD, will be contacted by page as soon as possible to help with clinical management. If the PI is unavailable, Todd Griswold (CMC Medical Director) can be consulted to cover.

If a participant describes feelings of suicidality while participating in a group, the study staff or group leader present during the group will help to manage this event with the current standard of care and will make immediate contact with the PI, Zev Schuman-Olivier, or clinical coverage listed above. The participant will be referred to the emergency room or the appropriate level of care, and the study staff member will contact the National Suicide Prevention Lifeline via the Google meet application. The PI will be notified within 24 hours of any suicidality.

8. INTERVENTION DISCONTINUATION

Participant Withdrawal/Termination Criteria:

Withdrawal/Termination criteria for Participants:

Participants may be discontinued from just the study intervention ("Group Discontinuation") OR both the study intervention and study assessments ("Study Withdrawal") at any time.

Specific reasons for discontinuing a participant ("Group Discontinuation") from the group are:

1. Voluntary discontinuation by the participant who is at any time free to discontinue his or her participation in the group, without prejudice to further treatment.

2. Safety reasons as judged by the investigator.
3. Substance use disorder or psychiatric symptoms worsen to a level that requires a higher level of care.
4. Revealing private information about other participants in the groups.

Specific reasons for withdrawing a participant ("Study Withdrawal") from the entire study are:

1. Voluntary discontinuation by the participant who is at any time free to discontinue his or her participation in the study, without prejudice to further treatment.
2. Safety reasons as judged by the investigator.
3. Incorrect enrollment (i.e., the participant does not meet the required inclusion/exclusion criteria) of the participant.
4. Active severe substance use disorder or level of intoxication during study procedures that precludes ability to conduct assessments.
5. Participants unable to complete baseline assessments.

Participants who discontinue will be asked about the reason(s) for their discontinuation and the presence of any adverse events. If possible, they will be seen and assessed by the Principal Investigator. Serious and Unexpected Adverse events will be followed up.

If participants are terminated from group intervention for any reason (except incorrect enrollment or safety concerns that endanger the study team), then they will still be expected to complete their outcome assessments, unless the participant revokes informed consent and withdraws from study.

Safety precautions for discontinuing a participant will be followed. Participants will be informed about discontinuation from group by the clinician leading the group and alternative treatment options will be discussed with the participant. If the participant is not available to receive this communication, then a letter will be sent informing the participant about the group discontinuation and providing alternative treatment options. Decisions about group intervention discontinuation will be made by the PI and group leaders but will be discussed with the CMC Medical Director for consultation prior to providing a group discontinuation letter.

If a participant is withdrawn from the study, they will be contacted by the Principal Investigator and informed the reason they are being withdrawn. A Withdrawal Letter will be sent informing the participant about the study withdrawal. If the participant

was still in a study group, then the group discontinuation process will also be completed as described above with attention to providing alternative treatment options.

Participation in this research study is voluntary. Participants can choose not to participate, and their decision will in no way affect the quality of care they receive at their site or their working relationship with their site. Participants may elect at any point to discontinue their participation in this study.

Participants who become pregnant during the study will not be discontinued. The current standard of care for women with opioid use disorder is to be maintained on medication-assisted treatment with methadone or buprenorphine during the pregnancy. In fact, buprenorphine treatment leads to shorter duration of neonatal abstinence syndrome than methadone[209], leading buprenorphine to be the gold standard for treatment during pregnancy. To reduce risk of fetal exposure to naloxone, the current standard is for pregnant participants to be maintained on buprenorphine alone (Subutex) instead of buprenorphine/naloxone (Suboxone) combination[210]; however, the American College of Obstetricians and Gynecologists reported in their clinical guidelines that it is possible that the combination medication (Buprenorphine/Naloxone) will increasingly be prescribed as multiple recent studies have supported its safety[211–214]. Importantly, in this study, we will not be prescribing buprenorphine to study participants, but rather enrolling pregnant participants taking buprenorphine prescribed by their health care provider as study participants into groups. If a participant becomes pregnant during the study, then the study team will alert the prescriber about the pregnancy and encourage the provider to follow their standard of care. Mindfulness groups during pregnancy have been shown in meta-analysis to reliably reduce anxiety, depression, and stress[215]. Preliminary studies suggest mindfulness groups are safe and acceptable for pregnant women with opioid use disorder[216].

<u>New Information</u>

Any pertinent new information will be communicated to study participants via e-mail if they have an e-mail address, otherwise via phone, by the research coordinator. In light of new information, participants may elect to discontinue their participation in the study.

9. STATISTICAL CONSIDERATIONS

9.1 General Design Issues

Retention can be a problem in OUD behavioral therapies studies. To support the attractiveness of the study and ensure outcome measure completion, we will provide $5 gift cards for each week that a participant attends a group session and $5 for each bi-weekly period in which all assigned surveys and toxicology screens have been completed. We will obtain phone number and email of 3 close contacts in the demographics form so that we can more easily reach participants who may not be attending study sessions. We will call participants every other week for brief engagement calls, which has worked to increase retention by 20% in previous studies and will call all participants who missed a session within 48 hours.

9.2 Sample Size and Randomization

Justification of Sample Size and Power Calculations:

This study will enroll an approximate of n = 236 and maximum of n = 280 participants who will sign informed consent and our power analyses have identified that we will have the sample to identify meaningful effects with an effective sample size of n=156 with 78 individuals in the M-ROCC arm and 78 individuals in the control group. We expect to randomize at least 192 participants (96 M-ROCC: 96 Control) over 8 months in order to have an effective sample size of n=156. We will aim to recruit participants with an active buprenorphine prescription from providers in U.S. states approved for recruitment by the IRB to enroll patients at a rate of approximately 30 participants per month, anticipating 19% loss during screening and baseline assessments, so we can randomize approximately 24 participants each month for 8 months.

Based on R21 urine toxicology data, we will focus our primary outcome analysis on the impact of M-ROCC on opioid use starting 12 weeks after intervention assignment. We estimate a mean of 4.67 negative tests for all illicit opioids (opiates, oxycodone, fentanyl, methadone) out of 6 random oral fluid toxicology tests conducted during the last 12 weeks in M-ROCC (SD 1.23) (n=78) and 4.11 negative tests for all illicit opioids in the control check-in group (SD 1.27) (n=78). We anticipate an effect size of Cohen's $d = 0.45$ for between-group difference in percentage of monthly positive toxicology screens. Participation of N=192 individuals randomized (with an effective sample size of 156 with 78 individuals in the M-ROCC arm and 78 individuals in the control group) allows for > 80% power to

detect an effect size of 0.45 for negative toxicology for all illicit opioids between M-ROCC and control arms. This power calculation was generated assuming a t-test of the difference between two means, a 1:1 intervention to control recruitment ratio, a type I error of 0.05, a two-sided comparison, and a design effect of 1.23 derived from an ICC of 0.01 with 8 group clusters with average cluster size of 24, which represents the average number of people randomized in each monthly cohort to either live-online M-ROCC or the live-online control group. This level of cluster was selected because it will encompass cohort effects. As the primary outcome will be analyzed in an intent-to-treat analysis, it is not sensitive to the number of individuals who drop out of the study after randomization. For secondary outcomes, based on pilot data and other relevant studies, we anticipate dropout from intervention in M-ROCC between 17-28% at 24-weeks and dropout in the control groups of 21-44% at 24-weeks[14,217–220].

In previous primary care studies, we have reported the impact of MTPC over 24 weeks on reductions in anxiety as measured by the PROMIS-ASF as $d = -0.72$ (n=81), $p<0.001$[166] and $d = -0.80$, $p<0.001$ (n=136)[187]. In our recent R21 pilot study, we reported significant Anxiety reductions over 24 weeks among patients with co-morbid anxiety at baseline ($\geq$55 at baseline) ($p < .05$, ES = -0.556). Given the likelihood of a small effect of control on Anxiety after 4 weeks of buprenorphine treatment[221], we anticipate an effect size of $d = -0.56$ for between-group change in PROMIS-ASF.

9.3 Definition of Populations

Intent-To-Treat analyses will be performed according to the following definition of the intent to treat population: All individuals who are randomized in the study, which is defined as those individuals who complete informed consent, meet inclusion criteria and do not meet exclusion criteria, complete all baseline assessments, are randomized and receive information about their randomization status.

9.4 Interim Analyses and Stopping Rules

This study will be stopped prior to its completion if: (1) the intervention is associated with adverse effects that call into question the safety of the intervention; (2) any new information becomes available during the trial that necessitates stopping the trial; or (3) other situations occur that might warrant stopping the trial. If the DSMB feels that a safety review to consider stopping the trial is required, then a safety review will be scheduled with the PI, and the IRB and sponsor will be informed. While no formal

interim analyses are planned, we do plan to provide regular data monitoring for consistency and to decrease missing values. During these checks, we will monitor the data for unusual patterns that might suggest issues related to safety or adverse effects.

9.5 Outcomes

9.5.1 Primary Outcome

Biochemically-confirmed opioid abstinence, which is defined as the number of two-week periods with a negative oral fluid test [negative for opiate, oxycodone, fentanyl, methadone] AND no self-reported illicit opioid use, based on weekly substance use self-report during the final 12 weeks (weeks 13-24) of the study (6 is the highest possible number of negative oral fluid toxicology screen time periods). Participants will be randomly selected to undergo a live-online supervised oral fluid screen at a rate of once every 2 weeks. Negative toxicology screens have been previously used to assess effectiveness of OUD treatment[14,222].

9.5.2 Secondary Outcomes

<u>Main Secondary Clinical Outcomes:</u>

1. Patient Report Outcomes Measurement Information System – Anxiety Short Form 8a (PROMIS-ASF)

2. Patient Report Outcomes Measurement Information System – Pain Interference Scale (PROMIS-PISF)

3. Positive oral fluid toxicology tests for Cocaine during the final 12 weeks (weeks 13-24) of the study.

4. Positive oral fluid toxicology tests for Benzodiazepines during the final 12 weeks (weeks 13-24) of the study.

<u>Exploratory Self-regulation mechanistic outcomes</u>

1. Brief Experiential Avoidance Questionnaire (BEAQ) (EMO)
2. Difficulties in Emotion Regulation Scale (DERS-16) (EMO)
3. Perceived Stress Scale (PSS) (EMO)

4. Self-Compassion Scale (SCS) (SRP)
5. Multidimensional Assessment of Interoceptive Awareness subscales 1, 4-8 (MAIA) (SRP)
6. Nonattachment to Self Scale (NTS) (SRP)
7. Self-Critical Rumination Scale (SCRS) (SRP)
8. Experiences Questionnaire- Decentering Subscale (COG/EMO)

Exploratory Other Mechanistic outcomes:
1. Five Facet Mindfulness Questionnaire (FFMQ)
2. Interpersonal Mindfulness Scale (IMS)
3. Group Cohesion Questionnaire (GCQ)
4. M-ROCC Qualitative Interviews
5. Credibility/Expectancy Survey (OUD)
6. Credibility/Expectancy Survey (Stress)

Exploratory Clinical Outcomes:
1.Intervention retention at 24 weeks, defined as any **active BUP prescription** within the last 28 days (i.e., between Weeks 20-24 of participant enrollment).
2.Time to intervention discontinuation
3. Weekly Survey reports of BUP non-adherence [a] and diversion [b]
4. Medication-assisted treatment status at 24 weeks (including methadone, buprenorphine or naltrexone).
5. Self-reported average daily dose of BZDs prescribed over final 12 weeks
6. Beck Anxiety Inventory (BAI)
7. Pain Catastrophizing Scale (PCS)
8. Substance Craving Scale (SUBCS)
9. CAT-DI
10. CAT-ANX
11. CAT-PSY
12. CAT-MANIA
13. CAT-SUD
14. CAT- PTSD
15. CAT-SDOH

9.6 Data Analyses

Primary Outcome:
The **main primary outcome** is biochemically-confirmed illicit opioid abstinent periods (defined by a negative oral fluid test [negative for opiate, oxycodone,

fentanyl, methadone] AND no self-reported illicit opioid use), during the final 12 weeks of the study (with 6 being the highest possible number of oral fluid tests during that time period).

Negative toxicology screens have been previously used to assess effectiveness of OUD treatment[14,222]. We will use an intent-to-treat design to evaluate the extent to which M-ROCC impacts biochemically confirmed illicit opioid abstinence over 6 biweekly time periods during the final 12 weeks of the study, **using Generalized Estimating Equations (GEE)[223,224],** which accounts for the clustering of individual observations over time, with covariates. This analytic approach provides reliable estimates even when data are unbalanced. GEE is also robust against misspecifications of the working correlation matrix when specifying the correct dependent-variable distribution and canonical link function. The benefit of selecting the best correlation structure is the gains in efficiency. The Quasi-likelihood Under Independence Model Criterion (QIC) will be used to ensure the best fitting working correlation structure[225]. **For dichotomous outcomes**, we will specify a binomial distribution and a logit link function, and robust standard errors clustered at the individual-level. For **missing data**, we will implement multiple imputation methods in Stata (mi procedure). This technique creates multiple complete datasets, imputes missing values using a probabilistic simulation method (e.g., chained equations), analyzes each dataset, and uses standard rules to combine estimates and adjust standard errors for the uncertainty of imputation[226].

Secondary Outcomes:

A main **secondary outcome** is change in PROMIS-ASF[227] at 24 weeks. To evaluate Anxiety as a secondary outcome, we will conduct a difference-in-differences, Intent-To-Treat, repeated measures analysis using linear mixed effects models (mixed) to evaluate time × intervention assignment interaction from baseline to 24 weeks, using a selected sample of participants with a baseline PROMIS-ASF> 16 (T-score > 55), which is the cutoff for mild anxiety disorder (since PROMIS-ASF is non-linear below this score). An additional **secondary outcome** is change in PROMIS-Pain Interference[228] (PROMIS-PISF) at 24 weeks. Mixed effects models account for clustering of multiple observations within participants. We will compute contrasts of predictive margins to test for significant within-group changes and difference-in-differences (between-group) estimates over time. Between-group and within-group effect size (Cohen's *d*) will be computed based on the predictive margins generated from the mixed models. To address missing data, we will implement multiple imputation methods in Stata (mi procedure). This technique creates multiple complete datasets, imputes missing values using a probabilistic simulation method (e.g., chained equations), analyzes each dataset, and uses standard rules to combine

estimates and adjust standard errors for the uncertainty of imputation[229]. Other **secondary clinical outcomes** include the number of positive oral fluid tests for BZD or cocaine during the final 12 weeks of the study.

For secondary and exploratory outcomes, we will use the Benjamini-Hochberg false discovery rate (FDR) procedure[230], which accounts for multiple comparisons. We will implement FDR according to Cao et al.[231] in which a cutoff p value is determined for a family of similar variables and analyses (family-wise error rate = 0.05)[166,232,233]. Statistical significance will be determined for the following analysis families: between-group main secondary clinical outcomes (family size, n = 4), and exploratory self-regulation outcomes (n=8), exploratory other mechanistic outcomes (n=4), exploratory clinical/health outcomes (n=15), formal practices (n= 4), informal practices (n = 7), and resource use (n= 7) (see section 9.5.2 for details).

Exploratory analyses:

We will conduct mixed effects model analyses as described above to analyze self-regulation mechanistic outcomes, including Brief Experiential Avoidance Questionnaire (BEAQ), Difficulties in Emotion Regulation Scale (DERS-16), Perceived Stress Scale (PSS)[234], Self-Compassion Scale (SCS), Multidimensional Assessment of Interoceptive Awareness (MAIA-2), Nonattachment to Self Scale (NTS), Self-Critical Rumination Scale (SCRS), and Experiences Questionnaire-Decentering Subscale (EQD).

We will collect other mechanistic outcomes, including interpersonal variables related to the group as potential covariates or mediators, Group Cohesion, Attitude about group leader, interpersonal mindfulness, as measured by the Interpersonal Mindfulness Scale (IMS), and intervention expectancy as measured by the Credibility/Expectancy Survey[239]. Adverse childhood experiences[240], Substance Abuse and Addiction Core Tier 1[241] (substance use severity and demographics), and distance from clinic[242,243] will also be collected. We will also collect data on participant mindfulness using the Five Facet Mindfulness Questionnaire (FFMQ).We will collect weekly surveys about BUP adherence and drug use. We will ask about mindfulness practice dose in M-ROCC and recovery skills use in control arms. Attitudes about group leaders and group cohesion[244] will be measured at 8, 16, and 24 weeks.

We will conduct mixed effects model analyses as described above to analyze several exploratory clinical outcomes, including B*eck Anxiety Inventory*[245], *Pain*

Catastrophizing Scale, Substance Craving Scale and CAT-MH outcomes (CAT-DI, CAT-ANX, CAT-PSY, CAT-MANIA, CAT-SUD, CAT-PTSD, CAT-SDOH).
We will also analyze BUP non-adherence and diversion reports, as well as self-reported average daily dose of BZDs prescribed over final 12 weeks.

We will compare intervention retention at 24-weeks, which is defined as having an active BUP prescription in the past 28 days at the 24-week study visit. In the chance case of imbalance of assignment, we will add the following variables as measurable covariates in regression models since they are known to impact retention[246]: psychiatric diagnosis, age, gender, race/ethnicity, unemployment, previous opioid treatment[242], Hepatitis C[246], childhood trauma[247], baseline cocaine use[14], and injection drug use history[14]. We will also add any other baseline characteristics that may be significantly imbalanced after randomization. We will also include key data collected during the first 4 weeks of the intervention, i.e., BUP non-adherence, self-reported illicit opioid use.

We will also measure time to intervention discontinuation, and fit Cox proportional hazard models that model the risk of time to intervention discontinuation, comparing hazard rates across the intervention and control groups, adjusting for the covariates described above in the logistic regression. All individuals that do not discontinue the intervention at the end of data collection will be considered to be right-censored. We will assess the Cox proportional hazards assumptions using tests of Schoenfeld residuals and by visual estimation of log-log plots of the estimated survival curves. We will also use Wald tests to test significance in intervention vs. control group hazard rate differences.

We will also conduct a minimum of 12 qualitative interviews for M-ROCC or control group completers to examine themes regarding online mindfulness delivery and to compare responses with our R21 qualitative outcomes from our in-person group model.

Because little is known about the dose effect or the impact of long term (> 8 weeks) mindfulness training, in exploratory analyses, we will evaluate the specific impact of MTPC intensive mindfulness sessions among those in M-ROCC who choose to continue their participation by joining MTPC, tracking their 24-week outcomes compared to the control arm that receives no mindfulness and participants that only did LDM. There is insufficient power to formally randomize and test this third arm that receives intensive MTPC, so we opt for this naturalistic design to provide exploratory data on dose effects (# of mindfulness sessions, total practice mins during 24-weeks, ≥12 group sessions, and participation in intensive MTPC-OUD). Those

who continue through MTPC are likely to differ at baseline from other study participants. Employing **propensity score (PS)** methods[248] allows us to minimize bias by balancing the MTPC, LDM only, and control groups on observable intervention eligibility criteria and potential outcome confounders. We will consider PS approaches including weighting, matching (nearest neighbor, optimal, ratio, full), or sub-classification[249]. We will select methods providing the best covariate balance across potential groups, graphically (range/density) and empirically (%-standardized difference in means ≤10%).

10. DATA COLLECTION AND QUALITY ASSURANCE

10.1 Data Collection Forms

Sources of Research Materials for Participants:

The following research material will be obtained:

1. Responses to surveys stored in REDCap
2. Audio tapes of M-ROCC sessions for fidelity ratings
3. Saliva samples (Oral Fluid Toxicology Screenings)

The following is the full list of measures for this study.

CAT-MH Interviews

Participants are sent a link to complete the CAT-MH (Computer Adaptive Testing for Mental Health) interview on a computer, tablet or phone. They can read or listen to the questions. The interview is delivered via a secure CHA IT-approved process using CHA's HIPAA-compliant REDCap server. ATT is the company who developed the CAT-MH software for behavioral health measurement. For this study, we will have an external module in REDCap that integrates CAT-MH into CHA's existing REDCap server. In this way, all patient data will exist on our side of the firewall. Since these CAT-MH assessments will only be used for research purposes, there will be no need for participants to have a direct connection to CHA in order to access the CAT-MH questionnaires that will be distributed through REDCap. Items from each of the modules for depression (CAT-DI)[189,250], anxiety (ANX-CAT)[251], mania and hypomania (M/HM-CAT)[189], PTSD (PTSD-CAT)[189], psychosis (PSY-S-CAT), social determinants of health (CAT-SDOH), and substance abuse (SUD-CAT) [252,253] are chosen from large item banks based on multidimensional item response theory, adapting each item presented to the individual's severity so that different individuals

are tested with different items depending on their severity level[250]. This allows for rapid testing – 2-12 minutes, depending on the number of domains tested – compared to 1-1.5 hours for a structured clinical interview, and greater precision. It is easy for patients to fill in online[189]. We monitor CAT-MH results daily and any rapid change in psychiatric symptoms is brought to the attention of study staff, including either medical director or PI. Moderate or severe suicide risk immediately triggers an email sent to the RC, M-ROCC clinical director (Fredericksen), medical director (Todd Grisworld) and the PI, and appropriate outreach by a study clinician and referral to the appropriate level of evaluation or psychiatric care will ensue. Duration: 2-12 min.

Montreal Cognitive Assessment - Blind (MOCA-BLIND)[204]: The MOCA was designed as a rapid screening instrument for mild cognitive dysfunction. The MOCA-BLIND is comprised of the same tasks as the original MOCA, with visual elements removed. It assesses different cognitive domains: attention and concentration, memory, language, conceptual thinking, calculations, and orientation. The total possible score is 22 points; a score of 16 or above is considered normal in substance use disorder context. Duration: 10 min.

A brief **demographics survey** will note participant race, ethnicity, primary language, income, address (including the street, unit/apartment number if applicable, city, state, and zip code), phone number and e-mail address of three close contacts, Facebook and Instagram handle, name and phone number of buprenorphine prescriber, and other quantifiable attributes. Duration: 4 min.

Scheduling Acceptability Survey: This survey will ask participants to rate, on a scale of 1-10, the likelihood that they can attend the day/time that the current control and LDM/MTPC groups are being held.

Baseline Battery for Participants:

A **Survey of Meditation and Mindfulness Experience (SMME)** will assess the participant's previous experience with meditation and Mindfulness-based interventions, both over their lifetime and in the past two weeks. This will be a brief questionnaire. Duration: 3 min.

Adverse Childhood Experiences (ACE) Questionnaire: The Adverse Childhood Experienced (ACE) Questionnaire[254] is a 10-item self-report measure that identifies childhood experiences of physical, sexual, and emotional abuse; neglect by parents or caregivers; violence between parents or caregivers; other kinds of serious household dysfunction such as alcohol and substance abuse; and peer violence,

witnessing community violence, and exposure to collective violence. Participants indicate “yes” or “no” to having experienced each type of ACE, and the score is calculated by summing up the number of questions to which they responded “yes.” Duration: 4 min

Substance Abuse and Addiction Core: Tier 1. We will use questions from this PhenX (www.phenxtoolkit.org) core toolkit related to assess age of initiation of first use, lifetime use, and past 30 days use, for all major substances of abuse, including alcohol and tobacco. We will only ask participants the items that are not redundant with the demographic survey above. The Core Collection includes highly recommended measures deemed by the SAA Scientific Panel (SSP) as broadly relevant to addiction research. This will be assessed verbally with a member of the study staff asking the questions out loud to the research participants. Duration: 10 min.

Clinical Assessment Battery for Participants:

The **Patient Reported Outcomes Measurement Information System – Anxiety Short Form 8a (PROMIS-ASF)**[255] is an 8-item scale used to assess patient-reported health status for anxiety. PROMIS instruments are funded by the National Institutes of Health (NIH), developed using Item Response Theory (IRT) and used to reliably and validly measure patient-reported outcomes for clinical research and practice. Participants are asked to rate their experience of the item in the past seven days on a 5-point scale from 1 (*Never*) to 5 (*Always*). With use of the PROMIS Assessment Center Scoring Service, a T score is generated from participant responses. A sample item includes “My worries overwhelmed me.” Duration: 3 min.

The **Patient Reported Outcomes Measurement Information System – Pain Interference 8a (PROMIS-PISF)**[255,256] The PROMIS Pain Interference instruments measure the self-reported consequences of pain on relevant aspects of one’s life. This includes the extent to which pain hinders engagement with social, cognitive, emotional, physical, and recreational activities. Pain Interference also incorporates items probing sleep and enjoyment in life, though the item bank only contains one sleep item. The Pain Interference short form is universal rather than disease-specific. It assesses pain interference over the past seven days. each question has five response options ranging in value from one to five (for pediatrics and parent proxy it is 0 to 4). To find the total raw score for a short form with all questions answered, sum the values of the response to each question. For example, for the adult 6-item form, the lowest possible raw score is 6; the highest possible raw score

is 30. A higher PROMIS T-score represents higher consequences of pain. Duration: 3 min.

The **Beck Anxiety Inventory (BAI)** consists of 21 items with a Likert scale ranging from 0 to 3 and raw scores ranging from 0 to 63. It was developed in 1988[257] and a revised manual was published in 1993 with some changes in scoring[245]. The BAI scores are classified as minimal anxiety (0 to 7), mild anxiety (8 to 15), moderate anxiety (16 to 25), and severe anxiety (30 to 63). Duration: 5 min.

Oral Fluid Toxicology Testing[95]:

The minimally invasive nature of sample collection is a key advantage of oral fluid over traditional biospecimens. An IDE is not required for these saliva tests because they are non-invasive and are not being evaluated to determine safety or effectiveness. The two dominant methods are collection of whole saliva by passive drool and collection of saliva by absorbent material placed in the mouth. A sponge method of collection will be used for oral fluid toxicology testing. Oral fluid toxicology testing will be supervised via videochat with a study staff member to ensure that there is no tampering with the collection.

Preparation: All subjects will be sent a package after enrollment and initial screening with sponge-based oral fluid toxicology testing kits. Tests will be sent at screening to participants' homes to be taken at randomly selected time points during the intervention or control group (at least 9 samples will be collected for each participant with at least 6 between weeks 13-24).

Prior to the online group, each group will also include a weekly check-in time before group or during group, during which oral fluid tests will be performed via Zoom in supervised breakout rooms with 3-6 randomly selected individuals each week (during the last 12 weeks of the intervention or control group). This process takes about 15 minutes in total. During this time, participants are required to video chat with a research coordinator to ensure that the results are properly recorded and that there is no tampering with the samples. First participants will unscrew the collector cap and pull out the collection stick with the sponge from the collection chamber. Next, participants will put the collector stick between their tongue and cheek to collect oral fluid by swabbing the inside of their mouth and then gently chewing the sponge until the saturation stick turns red; this takes a total of 3 minutes. Once this is complete, participants remove the sponge from their mouth, and place the collection stick into the collection chamber. Finally, participants secure the cap and shake three times. At this time, the research coordinator will start the timer for 10 minutes and

ensure that the collection chamber will be in clear view. Afterwards, the research coordinator will ask the participant to peel off the label. Results will be recorded by a saved screenshot image using the secure HIPAA compliant tool "Droplr" [258]. These screenshots will be uploaded to Droplr's cloud server before being transferred to CHA's secure google drive with the study ID and acrostic and date of test as the only identifiers. The research coordinator will record the results in REDCap, which will be double checked by another RC. Duration: 15 min.

These oral fluid toxicology screens can only be used for research purposes, as they are not FDA cleared for clinical use. We will be clear with patients and providers that these tests are only for research purposes. We will not provide any patient's oral fluid screen data to their provider, however we will refer to these results where necessary to provide the full context of an AE and encourage them to conduct a toxicology test with a clinically-approved urine toxicology screen. We will be clear that providers cannot make a clinical decision that impacts their patients' treatment plan based on this research screen.

Weekly Survey: Participants will receive a weekly password protected REDCap survey gathering information about their substance use in the last week, retention, buprenorphine adherence, their level of care (e.g., at a detox residential place, regular outpatient, primary care, etc.), enrollment in medication-assisted treatment, as well as their Buprenorphine and Benzodiazepine (if applicable) dosages. This survey will be sent via an email link to the secure, encrypted REDCap database. If this is impossible this information will be collected using a CHA-IT approved device. Duration: 4 min

Pain Catastrophizing Scale (PCS): The Pain Catastrophizing Scale (PCS)[259] is a 13-item scale designed to facilitate research on the mechanisms by which catastrophizing impacts pain experience. Participants will be asked to indicate the degree to which they experience certain thoughts and feelings when experiencing pain (e.g. "I feel I can't go on") on a scale from 0 ("Not at all") to 4 ("All the time"). Duration: 3 min.

Substance Craving Scale (SUBCS): The Substance Craving Scale (SUBCS) is a 6-item scale adapted from the Opioid Craving Scale (OCS)[260,261]. The SUBCS assesses participant craving for Opioids (including fentanyl, heroin, methadone, oxycodone or other opioids) and other substance use (including illicit drugs [e.g., cocaine, methamphetamine, etc.], using unprescribed pills [e.g., Adderall, etc.], using prescribed medication in ways or at doses for which they were not prescribed [e.g., Klonopin, etc.], or drinking alcohol). Duration: 2 min.

Self-Regulation Battery:

Brief Experiential Avoidance Questionnaire (BEAQ)[262]**:** The 62-item Multidimensional Experiential Avoidance Questionnaire (MEAQ) was recently developed to assess a broad range of experiential avoidance (EA) content. However, practical clinical and research considerations made a briefer measure of EA desirable. Using items from the original 62-item MEAQ, a 15-item scale (BEAQ) was created that tapped content from each of the MEAQ's six dimensions. Items were selected on the basis of their performance in 3 samples: undergraduates (n = 363), psychiatric outpatients (n = 265), and community adults (n = 215). These items were then evaluated using 2 additional samples (314 undergraduates and 201 psychiatric outpatients) and cross-validated in 2 new, independent samples (283 undergraduates and 295 community adults). The resulting measure (Brief Experiential Avoidance Questionnaire; BEAQ) demonstrated good internal consistency. It also exhibited strong convergence with respect to each of the MEAQ's 6 dimensions. The BEAQ demonstrated expected associations with measures of avoidance, psychopathology, and quality of life and was distinguishable from negative affectivity and neuroticism. Duration: 3 min

The **Difficulties in Emotion Regulation (DERS-16) Scale**[263,264] is a 16-item self-report scale designed to assess emotional dysregulation. The scale is a shortened version of the original 36-item scale. The scale assesses 5 aspects of emotional dysregulation: non-acceptance of emotional responses ("When I'm upset, I feel like I am weak"), difficulties engaging in goal directed behavior ("When I'm upset, I have difficulty thinking about anything else"), impulse control difficulties ("When I'm upset, I lose control over my behaviors"), limited access to emotion regulation strategies ("When I'm upset, I believe that there is nothing I can do to make myself feel better"), and lack of emotional clarity ("I have difficulty making sense out of my feelings."). Duration: 3 min.

The **Perceived Stress Scale (PSS) Scale**[234] uses 14 items to measure the degree to which situations in life are stressful. Items are designed to evaluate how overloaded, unpredictable, and uncontrollable one finds one's life. Each item is scored on a 5-point Likert scale from 0 (*Never*) to 4 (*Very often*). An example question is, "In the last month, how often have you felt difficulties were piling up so high that you could not overcome them?" Positively stated items are reverse scored before all scale items are summed to yield a total score. Duration: 3 min.

The **Self-Compassion Scale (SCS)**[265] is a 26-item Self-Compassion Scale. This scale evaluates six different aspects of self-compassion: Self-Kindness (e.g., ‘‘I try to be understanding and patient toward those aspects of my personality I don’t like’’), Self-Judgment (e.g., ‘‘I’m disapproving and judgmental about my own flaws and inadequacies’’), Common Humanity (e.g., ‘‘I try to see my failings as part of the human condition’’), Isolation (e.g., ‘‘When I feel inadequate in some way, I try to remind myself that feelings of inadequacy are shared by most people”), Mindfulness (e.g., ‘‘When something painful happens I try to take a balanced view of the situation’’), and Over-Identification (e.g., ‘‘When I’m feeling down I tend to obsess and fixate on everything that’s wrong.’’). The scale is scored on a 5-point Likert scale (1 = Almost never; 5 = Almost always), and negative subscale items are reverse scored. Duration: 5 min.

The **Multidimensional Assessment of Interoceptive Awareness (MAIA-2)**[266] is a 37-item self-report scale designed to assess multiple aspects of interoception and interoceptive awareness. This study will ask participants to complete 26 items including subscales 1,4-8 of the MAIA. The adapted scale assesses 6 aspects of interoceptive awareness: noticing (“I notice when I am uncomfortable in my body”), attention regulation (“When I am in conversation with someone, I can pay attention to my posture”), emotional awareness (“I notice that my breathing becomes free and easy when I feel comfortable”), self-regulation (“When I am caught up in thoughts, I can calm my mind by focusing on my body/breathing”), body listening (“I listen to my body to inform me about what to do”), and trusting (“I feel my body is a safe place”). Duration: 5 min.

The **Nonattachment to Self Scale (NTS)**[267] comprises 7 items rated on a seven-point Likert scale (1 = strongly disagree; 7 = strongly agree) measuring the degree to which the person releases fixation on self-related thoughts and feelings. Sound psychometric properties were established including good reliability (Cronbach's α = 0.84; test-retest reliability: r = 0.80), construct validity and criterion validity[267]. Internal consistency was good in the present samples (Cronbach's α = 0.88 and 0.87 in the BD and general population sample respectively), and NTS was found to be negatively associated with hypomanic symptoms in bipolar population[267,268]. Duration 2 min.

The **Self-Critical Rumination Scale (SCRS)**[269] is a 10-item scale measuring the degree to which the person repeatedly evaluates themselves negatively. Specifically, the scale measures constructs such as shame and self-criticism. Participants respond on a 7-point Likert scale from “not at all” to “very well.” Sample items from the

scale include, “My attention is often focused on aspects of myself that I’m ashamed of” and “I criticize myself a lot for how I act around other people.”

Experiences Questionnaire Decentering Subscale (EQD)
This is a subset of the 20-item self-report measure of experiences, consisting of 11 questions measuring decentering[80]. Decentering is conceptualized as a protective factor and capable of measuring resilience to depressive relapse. The EQD uses a 5-point Likert scale with responses from “never” to “all the time”. A sample item from the decentering subscale is “I can observe unpleasant feelings without being drawn into them.” Psychometric properties are: reliability: Cronbach’s $\alpha = .89$; convergent validity: $r > .46$; and divergent validity: $r < -.35$[270]. Duration: 5 min.

Mechanism Battery

Interpersonal Mindfulness Scale (IMS)[239]***:*** This is a 27-item scale that assesses trait mindfulness in the context of interpersonal interactions and relationships. Factors include Presence, Awareness of Self and Others, Nonjudgmental Acceptance, and Nonreactivity. Sample items include: “I think about the impact my words may have on another person before I speak” and “When I receive an angry text/email from someone, I try to understand their situation before responding.” Participants are asked to rate their response from 1 (almost never) to 5 (almost always). Duration: 4 min.

The **Five Facet Mindfulness Questionnaire (FFMQ)**[271] is a 39-item scale that examines five factors that represent aspects of the current empirical conception of mindfulness. These five facets include: observing, describing, acting with awareness, non-judging of inner experience, and non-reactivity to inner experience. An example item is “I pay attention to how my emotions affect my thoughts and behavior.” Participants rate their degree of agreement with each of the items on a Likert-type scale ranging from 1 (*Never or very rarely true*) to 5 (*Very often or always true*), with higher scores indicating higher experience of mindfulness. Duration: 6 min.

Attitudes about group survey:
The **Attitudes about group survey** is an adapted version of the **Group Cohesion Questionnaire (GCQ)**[272] which uses only the engagement subscale, consisting of 5 Likert scale questions that describe positive working group atmosphere. Duration: 2 min

Weekly Mindfulness Practice Diary Card:

The **Weekly Mindfulness Practice Diary Card** will be completed weekly while the participant is attending groups (for participants in the M-ROCC intervention group). Carmody et al[273] emphasizes that improvements in mindfulness, symptoms, and wellbeing are significantly related to formal and informal mindfulness practice. This card is a 4-item survey that asks participants to specify the type and duration of formal mindfulness techniques they completed each day for the past week, as well as the type and frequency of informal techniques each day. Formal mindfulness techniques include body scan, sitting meditation, loving kindness, mindful movement, and informal mindfulness practices include techniques like connecting with breath and mindful awareness of eating. The survey also asks about the type and frequency of use community or mobile mindfulness resources. Mobile resources might include apps such as Headspace, Stop/Breath/Think, or Insight Timer, and community resources would include online recordings from the CMC or UCSD websites. A participant's total practice for the week will be calculated by study personnel, based on the information on the diary card that will collected by group leaders weekly. This will also be completed by all participants during the Follow-up Survey Sessions, at participant study weeks 8, 16, and 24. This survey will be sent via an email link to the secure, encrypted REDCap database. Duration: 2 min.

Weekly Recovery Skills Diary:

The **Weekly Recovery Skills Diary** will be completed weekly while the participant is attending groups. This 5-item survey asks participants to specify the average number of times per day (over the past week) that they have engaged in recovery-related activities. Sample items include "Choose to be honest with myself and others," "Call or meet up with other people in recovery or attend mutual support meetings," and "Avoid high-risk situations, such as triggers, people, or places that remind me of opioids." This survey will be sent via an email link to the secure, encrypted, password protected REDCap database. Duration: 2 min.

Adverse Events Self-Reporting Battery:

The **Adverse Event Patient Participant Self-Report Form** will be systematically assessed at each assessment time point (8-weeks, 16-weeks, and 24-weeks). In addition, group leaders and research coordinators will be trained to identify and report any adverse events that occur or are reported during weekly live-online group visits. All AEs will be reviewed monthly by the PI, and SAE's will be reviewed within 24 hours. Please see adverse events section (7.3) above. Duration: 2 min

Credibility/Expectancy Survey (OUD)

The **Credibility/Expectancy Survey** will be completed at week 2, after the

participant has been randomized to their study arm. This 6-item survey will assess intervention expectancy and rationale credibility for the participant's assigned intervention arm in regards to opioid use reduction. An example question is "At this point, how much do you really feel that this program will help you to reduce your opioid use?". Duration: 3 min.

Credibility/Expectancy Survey (Stress)

The **Credibility/Expectancy Survey** will be completed at week 2, after the participant has been randomized to their study arm. This 6-item survey will assess intervention expectancy and rationale credibility for the participant's assigned intervention arm in regards to stress reduction. An example question is "At this point, how much do you really feel that this program will help you to reduce your stress?". Duration: 3 min.

10.2 Data Management

Data Storage and Data Sharing/Transfer

The below outlines data storage in this study, which data will be stored, and what PHI will be stored if applicable. Only CHA IRB approved study staff will have access to the below, which summarizes the entirety of data collected in this study. Data will not be shared from CHA to primary care sites at other institutions. ATT will send downloads of the coded CAT-MH data through a previously CHA IT-approved process on a weekly basis to our G-Drive. All data collection will be centralized to the following locations below. Study staff will be the only individuals who have access to the sources below. We will not collect any data that is not outlined below. Data will flow one-way from participants to the data storage location, which will be maintained and overseen by CHA. This information will be outlined in the participant ICF. Participants may request access to their own attendance and oral fluid toxicology screen results. The study team will mail these results on a monthly basis, and will not make any recommendations for what constitutes appropriate use of these results.

CHA Secure G-Suite Databases: Secure CHA G-suite database access will be granted through secure logins to CHA IRB approved study staff only. All data kept in G-suite databases will be identified by study number only, with the exception of the one linking sheet that links name and study number, and the G-suite folder where the pdfs of consent form signatures are kept. CAT-MH data will be transferred by ATT to a confidential G-suite Drive for CHA MindWell that they have access to deliver file drops once a week. Study status tracking for each participant will be maintained on a

G-suite database. Audio-video recordings of group sessions will be recorded by Zoom, saved to a coordinators computer but only accessible within the CHA Zoom research account on the coordinator's computer, then uploaded to a G-suite database and erased from the coordinator's computer Zoom account. Study status tracking for each participant will be maintained on a G-suite database. Any notes taken by study staff during any portion of the study will be recorded in a google document within our secure G Drive. All study staff will be alerted to the fact that no paper notes will be recorded throughout the trial due to privacy issues related to working from home during COVID-19.

PHI kept on Secure G-suite databases:

Study Inquiry Contact Form: Name, phone number, email address

Screening database: Name, birthday, medical diagnosis, phone number, email address

Uploaded recordings of zoom group sessions

Linking database: Name, study number, study acrostic

Consent form folder, stored in secure G-Suite drive separate from all other study documents: PDFs of consent form signature pages uploaded

Secure REDCap Databases:

Electronically signed informed consent forms and informed consent assessments will be stored in REDCap and completion will be visible from REDCap dashboard. All participant survey responses will be kept in a secure REDCap database. Only CHA IRB-approved study staff will have access to these databases. REDCap (Research Electronic Data Capture)[274] is a secure, HIPAA compliant, web-based application designed to support data capture for research studies. This platform provides the following elements: 1) an intuitive interface for validated data entry; 2) audit trails for tracking data manipulation and export procedures; 3) automated export procedures for seamless data downloads to common statistical packages; and 4) procedures for importing data from external sources. The REDCap software was developed by Vanderbilt University and has been obtained and installed for usage at the Cambridge Health Alliance. Online REDCap surveys will be emailed to participants using a password protected link.

PHI kept on REDCap database: demographic and survey response data (home address including street, unit/apartment number if applicable, city, state, and zip code, e-mail address, and age), oral fluid toxicology screen results, BUP prescription information (dose, frequency, dispense number, prescription date).

Online Database Storage: Given the online nature of this study and complications associated with the COVID-19 pandemic, all data will be stored using online secure databases.

CHA IT Electronic Device: If a participant does not have internet and video camera access, they may receive a CHA IT approved iPhone SE smartphone with unlimited data for the duration of the study in order to facilitate survey completion and group adherence. Participants will be mailed these study phones after randomization to their study arm. Alternatively, participants may receive reimbursement towards an unlimited data upgrade in the form of an online gift card to facilitate survey completion and group adherence, should they provide documentation of their data plan upgrade. Participants will be able to use the phone to access REDCap survey links, and to participate in groups via Zoom.

PHI kept on electronic devices: None.

Data Linkage to participants and access to data:
The secure web application REDCap will be used for data collection. Data will be entered directly into REDCap by participants. Any data entry not conducted by participants will be conducted only by members of the research team. All data collection will take place under the supervision of the Principal Investigator (Dr. Schuman-Olivier, MD) or a Research Coordinator. Substitute codes will be used to label all sources of participant information and access to identifiable data will be limited to researchers directly involved in the study. The Data Analyst will be responsible for coding data and exporting coded data from REDCap to STATA (14 or newer version), R (3.5.3 or newer version), or SAS (9.4 or newer version). All identifiable data will be destroyed 7 years after study completion and will not be maintained for future uses not specified in this research plan.

Data Sharing

The data generated in this grant will be presented at national or international conferences and published in a timely fashion. All final peer-reviewed manuscripts that arise from this proposal will be submitted to the digital archive PubMed Central. Primary outcome data will be deposited to appropriate public repositories (e.g., Harvard Dataverse, Open Science Framework) prior to the time the main study findings are accepted for publication. This will be listed in the consent form.

We will ensure that the clinical trial is registered to ClinicalTrials.gov no later than 21 days after enrollment of the first participant, and that a summary of study results will

be available on ClinicalTrials.gov no later than 12 months after the primary completion date. Informed consent documents for the study will include a specific statement relating to posting of study information and results at ClinicalTrials.gov. CHA has an internal policy in place to ensure that clinical trials registration and results reporting occur in compliance with policy requirements.

Description of Plan for Data Quality and Management:
The PI and data analyst will review data collection, data completeness and accuracy as well as protocol compliance on a monthly basis.

Survey session data will be entered by participants into REDCap only. The data analyst will review all REDCap data collection forms on an ongoing basis for data completeness and accuracy as well as protocol compliance and provide a monthly report to the PI about missing data.

Adherence with expected study assessment visits will be reviewed twice monthly with PI, and twice yearly by the study statistician and DSMB.

Frequency of Review—The frequency of data review for this study differs according to the type of data and can be summarized in the following Data Quality Monitoring Table**.**

Table 3. Data Quality Monitoring Table

**Data type**	**Frequency of review**	**Reviewer**
Study progress, recruitment, ICF and I/E review, and safety	Weekly	Senior Research Coordinator (SRC)
Data collection, data quality/completeness/accuracy protocol compliance	Weekly	Senior Research Coordinator (SRC)
Study progress, recruitment, participant accrual (including compliance with protocol enrollment criteria)	Monthly	PI, SRC,<br>Data Analyst
Data collection, data quality/completeness/accuracy protocol compliance	Monthly	Data Analyst, SRC, PI,
Participant accrual (including compliance with protocol enrollment criteria)	Twice-yearly	PI, Investigator Meeting, DSMB
Status of all enrolled participants, as of date reporting	Twice-yearly	PI, Investigator Meeting, DSMB

Adherence data regarding study visits and intervention	Twice-yearly	PI, Study Methodologist, DSMB
AEs and study safety	Per occurrence & Monthly	PI
Minor Protocol Deviations	Per occurrence & Monthly	PI
Major Protocol Violations	Per occurrence	PI, IRB, DSMB, NCCIH
SAEs	Per occurrence	PI, IRB, DSMB
SAEs (unanticipated, serious, and possibly related to study)	Per occurrence	PI, IRB, DSMB, NCCIH

10.3 Quality Assurance

10.3.1 Training

Intervention group leaders will be clinical staff. Groups will be co-led by a trained CHA clinical staff member, along with a co-leader from either CHA or one of our affiliated primary care sites, trained by CMC to administer M-ROCC. Group leaders will not have access to any study documents or information. They will act in a study capacity. Control group leaders will be required to have at least 1 year of prior experience leading recovery support groups for patients with OUD.

<u>M-ROCC Group Leader Training:</u>

M-ROCC has particular competencies which enable its effective delivery, including use of MBI: TAC (Teaching Assessment Criteria)[275] during group leader training and fidelity

checklists from MTPC that include the inquiry behavior count fidelity structure adapted from MBRP[276]. All group leaders have the capacity to embody the qualities and attitudes of mindfulness within the process of the group leadership, to respond deftly to affect emerging within the group with a mindful and compassionate stance. All group leaders will have engaged in appropriate training, consisting of 8 weeks of personal MBSR, at least 6 months of regular personal practice, and 40- hours of MTPC group leader training, including a one-day workshop focused on the application of mindfulness theory to the OUD MAT population. Every group leader will commit to weekly mentorship for the first two MTPC-OUD cohorts and must maintain an ongoing personal practice. Finally, group leaders recognize that the group is part of a participatory learning and healing process, underpinned by recognition of our ‘common humanity’[277].

M-ROCC Group Leader Fidelity and Adherence

Fidelity and Adherence: MROCC group leaders will participate in videoconference or in-person weekly mentorship during the time they are leading first two M-ROCC groups (LDM and MTPC). All groups will be audio-recorded via zoom. In order to prevent drift from the manual, 10% of session audio records will be reviewed by trained experts, and all sessions will be assessed for adherence to the manual using fidelity checklists that are completed by research coordinators participating in the group.

We have enrolled 16 clinicians from five OBOT sites around Massachusetts into our group leader training pipeline and 12 clinicians across eight sites completed the four-day MTPC group leader training in March 2019, with future trainings planned for sites to expand their group leader capacity. Additional training is available if new staff join the site are interested in working with program. Within each institution, there is the capacity for a clinician to help cover multiple primary care sites, but clinicians can't be shared between institutions. The Clinical Director at CHA may be able to help with site coverage as a co-leader, if a time-limited coverage issue arises. These trained group leaders at each site will have the opportunity to co-lead a group with a trained CHA clinician with supervision.

10.3.2 Quality Control Committee

The Senior Research Coordinator will review enrollment reports, adverse events, data quality, missing data, database quality, event reports from group leaders, and other aspects of quality control address study each week.

An MTPC Faculty mentor (Gawande) will review 10% of audio-recordings of the intervention and review adherence checklists to ensure fidelity to the intervention.

Dr. Schuman-Olivier meets monthly with the senior research coordinator and data analyst to review enrollment, adverse events, data quality, missing data, database quality, event reports from group leaders, and other aspects of quality control.

10.3.3 Metrics

All self-report survey items for both primary and secondary measures are coded as required fields in REDCap to prevent missed items. During the RCT group leader

fidelity checklists are reviewed each week by the clinical director or other member of the fidelity assessment team. All data are checked each month for missingness within each measure and for missing surveys, by the data analyst.

10.3.4 Protocol Deviations

Protocol deviations are captured weekly in the research assistant and research coordinator meeting, which is overseen by the senior research coordinator, and in the study implementation weekly meeting, which is overseen by the PI. Deviations are documented in the Protocol Deviation log, which is reviewed monthly by the PI and is reviewed twice yearly by the DSMB, and annually by the CHA IRB.

10.3.5 Monitoring

Data Safety Monitoring Board:
We will have a Data Safety Monitoring Board that meets twice yearly, consisting of 3 members, with at least one substance use clinician researcher with expertise in Buprenorphine treatment (Joji Suzuki, MD), at least one member who has expertise in mindfulness-based interventions (Sarah Bowen, PhD), and at least one epidemiologist/statistician (Linda Valeri, PhD).

Safety Review Plan:

Study progress and safety will be reviewed monthly (and more frequently if needed). Progress reports, including participant recruitment, retention/attrition, and AEs, will be provided to the DSMB every 6 months for the DSMB meeting. An Annual Report will be compiled and will include a list and summary of AEs. In addition, the Annual Report will address (1) whether AE rates are consistent with pre-study assumptions; (2) reason for dropouts from the study; (3) whether all participants met entry criteria; (4) whether continuation of the study is justified on the basis that additional data are needed to accomplish the stated aims of the study; and (5) conditions whereby the study might be terminated prematurely. The Annual Report will be sent to the DSMB and will be forwarded to the IRB. The IRB and other applicable recipients will review progress of this study on an annual basis. The PI will also send copies of signed recommendations and comments from the DSMB to the NCCIH Program Officer within 30 days of each monitoring review.

Study Report Outline for the DSMB (Interim or Annual Reports):

The study team will generate Study Reports for the DSMB and will provide information on the following study parameters: rate of participant accrual and compliance with inclusion/exclusion criteria, status of all enrolled participants, adherence data regarding study visits and intervention, AEs, and protocol violations. Study report tables will be generated only from aggregate (not by group assignment) baseline and aggregate safety data for the study population. A separate Closed Safety Report, with masked group baseline and safety data, will be generated for the DSMB by a designated unmasked member of the team but will not be reviewed by the study PI.

This study will be stopped prior to its completion if: (1) the intervention is associated with adverse effects that call into question the safety of the intervention; (2) any new information becomes available during the trial that necessitates stopping the trial; or (3) other situations occur that might warrant stopping the trial.

The PI will include an assessment of futility (if relevant) in the annual progress report to NIH (using statistical means such as predictive probability, if appropriate) and will consult with the study monitors to assess the impact of significant data loss due to problems in recruitment, retention, or data collection. The study may also be discontinued at any time by the IRB, the NCCIH, or other government agencies as part of their duties to ensure that research participants are protected.

11. PARTICIPANT RIGHTS AND CONFIDENTIALITY

11.1 Institutional Review Board (IRB) Review

This protocol and the informed consent document and any subsequent modifications will be reviewed and approved by the IRB or ethics committee responsible for oversight of the study.

11.2 Informed Consent Forms

Informed consent will be obtained during the Informed Consent Session as described in Section 6 above. The consent session will begin with a verbal review of key information about the study. The consent form will describe the purpose of the study, the procedures to be followed, and the risks and benefits of participation. All informed consent documents are approved and reviewed on an annual basis by the

CHA IRB. A signed consent form will be obtained from each participant. Given that the intervention requires a 7th grade reading level and only adult participants can enroll, we will not obtain informed consent for this study from a person who requires a guardian.

11.3 Participant Confidentiality

All participants will be protected by a certificate of confidentiality automatically issued through the NIH. Confidentiality will be ensured by use of a unique numeric identification code and an acrostic that are unique to each study participant.

All research session data will be collected using standardized electronic forms on designed using the REDCap database hosted by Cambridge Health Alliance. REDCap (Research Electronic Data Capture) is a secure, web-based application designed to support data capture for research studies, providing an intuitive interface for validated data entry and export procedures to common statistical packages. All data management will be conducted by the research team operating from CHA. All data collection will take place under the supervision of the Dr. Schuman-Olivier or the research coordinator. Data will only be collected by members of the research team. Only CHA IRB-approved study staff will have access to the study REDCap databases, including the Research Coordinators. All REDCap access will be password protected. Site clinical staff will not have access to REDCap databases.

The name and birthday, will be entered into an initial CHA secured G-Suite database strictly for the purpose of screening and consent process. A unique numeric identifier and acrostic will be created for all participants who have completed an informed consent. The list linking any personal identifying information with the participant's study number and acrostic will be kept in a CHA secured G-suite file. All data will be linked to these identifiers and no direct participant identifiers will be transmitted from REDCap to STATA 14, R 3.5.3 or SAS 9.4 for analysis.

All further study data from survey sessions will be collected in a second REDCap database, which will reference this unique numeric identifier and acrostic.

<u>Database Protection:</u>

Participant informed consent, screening forms, and course documents will be stored in double-locked lockboxes or filing cabinets at each site. Copies of consent forms will be transferred to double-locked file cabinets at the Center for Mindfulness and Compassion.

Access to identifiable data will be limited to researchers directly involved in the study. All identifiable data will be destroyed 7 years after study completion and will not be maintained for future uses not specified in this research plan.

Minimization:

The risk of loss of confidentiality is judged to be minimal. Confidentiality will be maintained by disguising identifying information through the assignment of a numeric study number and an alphabetic study acrostic, and by keeping all data in the secure REDCap application. Any coded or de-identified data will be maintained in password protected databases. Participant information will be accessible only to study staff. Information about study participants will not leave our institution in any form that would identify individual participants. Data will be transmitted to STATA 14 or SAS 9.4 in a pooled form with participants identified only by numeric code and acrostic. In addition, we will ask group participants to agree to a group confidentiality agreement. This agreement would require that information shared within the group remain solely within the group. Study staff will also be required to use password protected computers connected to password protected WiFi when working remotely.

Confidentiality During Adverse Event (AE) Reporting

AE reports and annual summaries will not include participant- or group-identifiable material. Each report will only include the unique M-ROCC numeric identifier and acrostic.

11.4 Study Discontinuation

This study will be stopped prior to its completion if: (1) the intervention is associated with adverse effects that call into question the safety of the intervention; (2) difficulty in study recruitment or retention will significantly impact the ability to evaluate the study endpoints; (3) any new information becomes available during the trial that necessitates stopping the trial; or (4) other situations occur that might warrant stopping the trial.

During the funding of this study, any action by the IRB or one of the study investigators that results in a temporary or permanent suspension of the study will be reported to the NCCIH Program Official within 1 business day of notification.

12. COMMITTEES

The DSMB for this study includes Sarah Bowen, PhD, Linda Valeri, PhD, and Joji Suzuki, MD. See DSMP for details.

13. PUBLICATION OF RESEARCH FINDINGS

Any presentation, abstract, or manuscript will be made available for review by NCCIH prior to publication.

14. REFERENCES

1. Hedegaard, H., Miniño, A. M. & Warner, M. Drug Overdose Deaths in the United States, 1999-2018. *NCHS Data Brief* 1–8 (2020).
2. Warner, M., Chen, L. H., Makuc, D. M., Anderson, R. N. & Miniño, A. M. Drug poisoning deaths in the United States, 1980-2008. *NCHS Data Brief* 1–8 (2011).
3. Cicero, T. J. & Ellis, M. S. Effect of Abuse-Deterrent Formulation of OxyContin. *N. Engl. J. Med.* **367**, 187–189 (2012).
4. Tietjen, D. New England Governors to Work Together to Address Opioid Epidemic. *Boston Globe* http://www.boston.com/health/2014/06/18/new-england-governors-work-together-address-opioid-epidemic/oXImIQdvLWIO6Y4C76pXxJ/story.html (2014).
5. Fudala, P. J. *et al.* Office-based treatment of opiate addiction with a sublingual-tablet formulation of buprenorphine and naloxone. *N. Engl. J. Med.* **349**, 949–958 (2003).
6. Comer, S. D. *et al.* Abuse liability of intravenous buprenorphine/naloxone and buprenorphine alone in buprenorphine-maintained intravenous heroin abusers. *Addiction* **105**, 709–718 (2010).
7. Bell, J. R., Butler, B., Lawrance, A., Batey, R. & Salmelainen, P. Comparing overdose mortality associated with methadone and buprenorphine treatment. *Drug Alcohol Depend.* **104**, 73–77 (2009).
8. Rosenblatt, R. A., Andrilla, C. H. A., Catlin, M. & Larson, E. H. Geographic and specialty distribution of US physicians trained to treat opioid use disorder. *Ann. Fam. Med.* **13**, 23–26 (2015).
9. Zhang, Z., Friedmann, P. D. & Gerstein, D. R. Does retention matter? Treatment duration and improvement in drug use. *Addiction* **98**, 673–684 (2003).
10. Clausen, T., Anchersen, K. & Waal, H. Mortality prior to, during and after opioid maintenance treatment (OMT): A national prospective cross-registry study. *Drug Alcohol Depend* **94**, 151–57 (2008).
11. Hser, Y.-I. *et al.* Treatment Retention among Patients Randomized to Buprenorphine/Naloxone Compared to Methadone in A Multi-site Trial. *Addiction* **109**, 79–87 (2014).
12. Ferri, M., Finlayson, A. J. R., Wang, L. & Martin, P. R. Predictive factors for relapse in patients on buprenorphine maintenance. *Am. J. Addict.* **23**, 62–67 (2014).
13. Gerra, G. *et al.* Buprenorphine treatment outcome in dually diagnosed heroin dependent patients: A retrospective study. *Prog. Neuro-Psychopharmacology Biol. Psychiatry* **30**, 265–272 (2006).
14. Schuman-Olivier, Z., Weiss, R. D., Hoeppner, B. B., Borodovsky, J. & Albanese, M. J. Emerging adult age status predicts poor buprenorphine treatment retention. *J. Subst. Abuse*

Treat. **47**, 202–212 (2014).
15. Sullivan, L. E. *et al.* The Association between Cocaine Use and Treatment Outcomes in Patients Receiving Office-Based Buprenorphine/Naloxone for the Treatment of Opioid Dependence. *Am. J. Addict.* **19**, 53–58 (2010).
16. Worley, M. J., Heinzerling, K. G., Shoptaw, S. & Ling, W. Volatility and change in chronic pain severity predict outcomes of treatment for prescription opioid addiction. *Addiction* **112**, 1202–1209 (2017).
17. Bramness, J. & Kornør, H. Benzodiazepine prescription for patients in opioid maintenance treatment in Norway. *Drug Alcohol Depend.* **90**, 203–209 (2007).
18. Lintzeris, N. & Nielsen, S. Benzodiazepines, methadone and buprenorphine: interactions and clinical management. *Am. J. Addict.* **19**, 59–72 (2010).
19. Schuman-Olivier, Z. *et al.* Benzodiazepine use during buprenorphine treatment for opioid dependence: Clinical and safety outcomes. *Drug Alcohol Depend.* **132**, 580–586 (2013).
20. Reynaud, M., Petit, G., Potard, D. & Courty, P. Six deaths linked to concomitant use of buprenorphine and benzodiazepines. *Addiction* **93**, 1385–1392 (1998).
21. Otto, M. W., Bruce, S. E. & Deckersbach, T. Benzodiazepine use, cognitive impairment, and cognitive-behavioral therapy for anxiety disorders: issues in the treatment of a patient in need. *J. Clin. Psychiatry* **66 Suppl 2**, 34–38 (2005).
22. Midgard, H., Bramness, J. G., Skurtveit, S., Haukeland, J. W. & Dalgard, O. Hepatitis C Treatment Uptake among Patients Who Have Received Opioid Substitution Treatment: A Population-Based Study. *PLoS One* **11**, e0166451 (2016).
23. Cullen, M. Mindfulness-Based Interventions: An Emerging Phenomenon. *Mindfulness (N. Y).* **2**, 186–193 (2011).
24. Baer, R. A. Mindfulness training as a clinical intervention: A conceptual and empirical review. *Clin. Psychol. Sci. Pract.* **10**, 125–143 (2003).
25. Kabat-Zinn, J. Mindfulness-based interventions in context: Past, present, and future. *Clin. Psychol. Sci. Pract.* **10**, 144–156 (2003).
26. Kabat-Zinn, J. An outpatient program in behavioral medicine for chronic pain patients based on the practice of mindfulness meditation: theoretical considerations and preliminary results. *Gen Hosp Psychiatry* **4**, 33–47 (1982).
27. Kabat-Zinn, J., Lipworth, L. & Burney, R. The clinical use of mindfulness meditation for the self-regulation of chronic pain. *J Behav Med* **8**, 163–190 (1985).
28. Khoury, B., Sharma, M., Rush, S. E. & Fournier, C. Mindfulness-based stress reduction for healthy individuals: A meta-analysis. *J. Psychosom. Res.* **78**, 519–528 (2015).
29. Kabat-Zinn, J. *et al.* Effectiveness of a meditation-based stress reduction program in the treatment of anxiety disorders. *Am. J. Psychiatry* **149**, 936–43 (1992).
30. Crane, R., Brewer, J., Feldman, C. & Kabat-Zinn, J. What defines mindfulness-based programs? The warp and the weft. *Psychol. Med.* **47**, 990–99 (2017).
31. Barnes, N., Hattan, P. R., Black, D. S. & Schuman-Olivier, Z. An Examination of Mindfulness-Based Programs in US Medical Schools. *Mindfulness (N. Y).* **8**, 489–494 (2017).
32. Newby, J. M., McKinnon, A., Kuyken, W., Gilbody, S. & Dalgleish, T. Systematic review and meta-analysis of transdiagnostic psychological treatments for anxiety and depressive disorders in adulthood. *Clin. Psychol. Rev.* **40**, 91–110 (2015).
33. Strauss, C., Cavanagh, K., Oliver, A. & Pettman, D. Mindfulness-based interventions for people diagnosed with a current episode of an anxiety or depressive disorder: A meta-

analysis of randomised controlled trials. *PLoS One* **9**, (2014).

34. Goyal, M. *et al.* Meditation programs for psychological stress and well-being: A systematic review and meta-analysis. *JAMA Intern. Med.* **174**, 357–368 (2014).

35. Hofmann, S. G., Sawyer, A. T., Witt, A. a & Oh, D. The Effect of Mindfulness-Based Therapy on Anxiety and Depression: A Meta-Analytic Review. *J. Consult. Clin. Psychol.* **78**, 169–183 (2010).

36. Blanck, P. *et al.* Effects of mindfulness exercises as stand-alone intervention on symptoms of anxiety and depression: Systematic review and meta-analysis. *Behav. Res. Ther.* **102**, 25–35 (2018).

37. Pascoe, M. C., Thompson, D. R., Jenkins, Z. M. & Ski, C. F. Mindfulness mediates the physiological markers of stress: Systematic review and meta-analysis. *J. Psychiatr. Res.* **95**, 156–178 (2017).

38. Hyman, S. M., Fox, H., Hong, K.-I. A., Doebrick, C. & Sinha, R. Stress and Drug-Cue-Induced Craving in Opioid-Dependent Individuals in Naltrexone Treatment. *Exp Clin Psychopharmacol* **15**, 134–143 (2007).

39. Mantsch, J. R., Baker, D. A., Funk, D., Lê, A. D. & Shaham, Y. Stress-induced reinstatement of drug seeking: 20 years of progress. *Neuropsychopharmacology* **41**, 335–356 (2016).

40. Koob, G. F. *et al.* Addiction as a stress surfeit disorder. *Neuropharmacology* **76**, 370–382 (2014).

41. Ribeiro, S. C., Kennedy, S. E., Smith, Y. R., Stohler, C. S. & Zubieta, J.-K. Interface of physical and emotional stress regulation through the endogenous opioid system and mu-opioid receptors. *Prog. neuro-psychopharmacology & Biol. psychiatry* **29**, 1264—1280 (2005).

42. Xu, G. P., Van Bockstaele, E., Reyes, B., Bethea, T. & Valentino, R. J. Chronic morphine sensitizes the brain norepinephrine system to corticotropin-releasing factor and stress. *J. Neurosci.* **24**, 8193–8197 (2004).

43. Lex, C., Bäzner, E. & Meyer, T. D. Does stress play a significant role in bipolar disorder? A meta-analysis. *J. Affect. Disord.* **208**, 298–308 (2017).

44. Cheng, S., Walsh, E. & Schepp, K. Vulnerability, Stress, and Support in the Disease Trajectory from Prodrome to Diagnosed Schizophrenia: Diathesis-Stress-Support Model. *Arch. Psychatric Nurs.* **30**, 810–817 (2016).

45. Gong, H. *et al.* Mindfulness meditation for insomnia: A meta-analysis of randomized controlled trials. *J. Psychosom. Res.* **89**, 1–6 (2016).

46. Margolin, A., Beitel, M., Schuman-Olivier, Z. & Avants, S. K. A controlled study of a spirituality-focused intervention for increasing motivation for HIV prevention among drug users. *AIDS Educ. Prev. Off. Publ. Int. Soc. AIDS Educ.* **18**, 311–322 (2006).

47. Margolin, A. *et al.* A Preliminary Study of Spiritual Self-Schema (3-S) Therapy for Reducing Impulsivity in HIV-Positive Drug Users. *J. Clin. Psychol.* **63**, 979–999 (2007).

48. Bowen, S., Somohano, V. C., Rutkie, R. E., Manuel, J. A. & Rehder, K. L. Mindfulness-Based Relapse Prevention for Methadone Maintenance: A Feasibility Trial. *J Altern Complement Med* **23**, 541–544 (2017).

49. Bowen, S. *et al.* Relative efficacy of mindfulness-based relapse prevention, standard relapse prevention, and treatment as usual for substance use disorders: a randomized clinical trial. *JAMA psychiatry* **71**, 547–556 (2014).

50. Grow, J. C., Collins, S. E., Harrop, E. N. & Marlatt, G. A. Enactment of home practice

following mindfulness-based relapse prevention and its association with substance-use outcomes. *Addict. Behav.* **40**, 16–20 (2015).

51. Penberthy, J. K. *et al.* Mindfulness-based relapse prevention: History, mechanisms of action, and effects. *Mindfulness (N. Y).* **6**, 151–158 (2015).
52. Witkiewitz, K. *et al.* Mindfulness-based treatment to prevent addictive behavior relapse: theoretical models and hypothesized mechanisms of change. *Subst. Use Misuse* **49**, 513–524 (2014).
53. Glasner-Edwards, S. *et al.* Mindfulness Based Relapse Prevention for Stimulant Dependent Adults: A Pilot Randomized Clinical Trial. *Mindfulness (N. Y).* **8**, 126–135 (2017).
54. Witkiewitz, K., Lustyk, M. K. B. & Bowen, S. Retraining the addicted brain: a review of hypothesized neurobiological mechanisms of mindfulness-based relapse prevention. *Psychol. Addict. Behav. J. Soc. Psychol. Addict. Behav.* **27**, 351–365 (2013).
55. Grant, S. *et al.* Mindfulness-based Relapse Prevention for Substance Use Disorders: A Systematic Review and Meta-analysis. *J. Addict. Med.* **11**, 386–396 (2017).
56. Zgierska, A. *et al.* Mindfulness meditation for substance use disorders: a systematic review. *Subst. Abus.* **30**, 266–294 (2009).
57. Wilson, A. D. *et al.* Mindfulness-based interventions for addictive behaviors: Implementation issues on the road ahead. *Psychol. Addict. Behav. J. Soc. Psychol. Addict. Behav.* **31**, 888–896 (2017).
58. Elwafi, H., Witkiewitz, K., S, M., Thornhill, Ta. & Brewer, J. Mindfulness training for smoking cessation: moderation of the relationship between craving and cigarette use. *Drug Alcohol Depend* **130 (1–3)**, 222–229 (2013).
59. Brewer, J. A., Elwafi, H. M. & Davis, J. H. Craving to quit: psychological models and neurobiological mechanisms of mindfulness training as treatment for addictions. *Psychol. Addict. Behav. J. Soc. Psychol. Addict. Behav.* **27**, 366–379 (2013).
60. Garland, E. L. *et al.* Pain, hedonic regulation, and opioid misuse: Modulation of momentary experience by Mindfulness-Oriented Recovery Enhancement in opioid-treated chronic pain patients. *Drug Alcohol Depend.* **173 Suppl**, S65–S72 (2017).
61. Garland, E. & Howard, M. Mindfulness-Oriented Recovery Enhancement Reduces Pain Attentional Bias in Chronic Pain Patients. *Psychother. Psychosom.* **82**, 311–318 (2013).
62. Phelps, C. L., Paniagua, S. M., Willcockson, I. U. & Potter, J. S. The relationship between self-compassion and the risk for substance use disorder. *Drug Alcohol Depend.* **183**, 78–81 (2018).
63. Van Dam, N. T., Sheppard, S. C., Forsyth, J. P. & Earleywine, M. Self-compassion is a better predictor than mindfulness of symptom severity and quality of life in mixed anxiety and depression. *J. Anxiety Disord.* **25**, 123–130 (2011).
64. Kelly, A. C., Carter, J. C. & Borairi, S. Are improvements in shame and self-compassion early in eating disorders treatment associated with better patient outcomes? *Int. J. Eat. Disord.* **47**, 54–64 (2014).
65. Holl, J. *et al.* Substance use to regulate intense posttraumatic shame in individuals with childhood abuse and neglect. *Dev. Psychopathol.* **29**, 737–749 (2017).
66. Matthews, S., Dwyer, R. & Snoek, A. Stigma and Self-Stigma in Addiction. *J. Bioeth. Inq.* **14**, 275–286 (2017).
67. Health NIo. Science of Behavior Change. *Office of Stategic Coordination - The Common Fund* https://commonfund.nih.gov/behaviorchange (2017).

68. Hölzel, B. K. *et al.* How Does Mindfulness Meditation Work? Proposing Mechanisms of Action From a Conceptual and Neural Perspective. *Perspect. Psychol. Sci. a J. Assoc. Psychol. Sci.* **6**, 537–559 (2011).
69. Fan, J., Raz, A. & Posner, M. Attentional Mechanisms. *Encyclopedia of Neurological Sciences* 292–299 (2003).
70. Fan, J., McCandliss, B. D., Sommer, T., Raz, A. & Posner, M. I. Testing the efficiency and independence of attentional networks. *J. Cogn. Neurosci.* **14**, 340–347 (2002).
71. Langner, R. & Eickhoff, S. Sustaining Attention to Simple Tasks: A Meta-Analytic Review of the Neural Mechanisms of Vigilant Attention. *Psychol. Bull.* **139**, 870–900 (2013).
72. Sturm, W. & Willmes, K. On the functional neuroanatomy of intrinsic and phasic alertness. *Neuroimage* **14**, S76-84 (2001).
73. Ridderinkhof, K. R., van den Wildenberg, W. P. M., Segalowitz, S. J. & Carter, C. S. Neurocognitive mechanisms of cognitive control: the role of prefrontal cortex in action selection, response inhibition, performance monitoring, and reward-based learning. *Brain Cogn.* **56**, 129–140 (2004).
74. Rueda, M. R., Posner, M. I. & Rothbart, M. K. The development of executive attention: contributions to the emergence of self-regulation. *Dev. Neuropsychol.* **28**, 573–594 (2005).
75. Dillon, D. G. & Pizzagalli, D. A. Inhibition of Action, Thought, and Emotion: A Selective Neurobiological Review. *Appl. Prev. Psychol. J. Am. Assoc. Appl. Prev. Psychol.* **12**, 99–114 (2007).
76. van Gaal, S., Lamme, V. A. F., Fahrenfort, J. J. & Ridderinkhof, K. R. Dissociable brain mechanisms underlying the conscious and unconscious control of behavior. *J. Cogn. Neurosci.* **23**, 91–105 (2011).
77. Schooler, J. W. *et al.* Meta-awareness, perceptual decoupling and the wandering mind. *Trends in Cognitive Sciences* vol. 15 319–326 (2011).
78. Critchley, H. D., Wiens, S., Rotshtein, P., Ohman, A. & Dolan, R. J. Neural systems supporting interoceptive awareness. *Nat. Neurosci.* **7**, 189–195 (2004).
79. Vago, D. R. & Silbersweig, D. A. Self-awareness, self-regulation, and self-transcendence (S-ART): a framework for understanding the neurobiological mechanisms of mindfulness. *Front. Hum. Neurosci.* **6**, 296 (2012).
80. Fresco, D. M. *et al.* Initial Psychometric Properties of the Experiences Questionnaire: Validation of a Self-Report Measure of Decentering. *Behav. Ther.* **38**, 234–246 (2007).
81. Bieling, P. J. *et al.* Treatment-specific changes in decentering following mindfulness-based cognitive therapy versus antidepressant medication or placebo for prevention of depressive relapse. *J. Consult. Clin. Psychol.* **80**, 365–372 (2012).
82. Valentine, E. R. & Sweet, P. L. G. Meditation and attention: A comparison of the effects of concentrative and mindfulness meditation on sustained attention. *Ment. Health. Relig. Cult.* **2**, 59–70 (1999).
83. Chan, D. & Woollacott, M. Effects of level of meditation experience on attentional focus: is the efficiency of executive or orientation networks improved? *J. Altern. Complement. Med.* **13**, 651–657 (2007).
84. Davidson, R. J., Goleman, D. J. & Schwartz, G. E. Attentional and affective concomitants of meditation: A cross-sectional study. *Journal of Abnormal Psychology* vol. 85 235–238 (1976).

85. Slagter, H. A. *et al.* Mental Training Affects Distribution of Limited Brain Resources. *PLOS Biol.* **5**, e138 (2007).
86. Lazar, S. W. *et al.* Functional brain mapping of the relaxation response and meditation. *Neuroreport* **11**, 1581–1585 (2000).
87. Brefczynski-Lewis, J. A., Lutz, A., Schaefer, H. S., Levinson, D. B. & Davidson, R. J. Neural correlates of attentional expertise in long-term meditation practitioners. *Proc. Natl. Acad. Sci.* **104**, 11483 LP – 11488 (2007).
88. Tang, Y.-Y. *et al.* Short-term meditation training improves attention and self-regulation. *Proc. Natl. Acad. Sci.* **104**, 17152 LP – 17156 (2007).
89. Srinivasan, N. & Baijal, S. Concentrative meditation enhances preattentive processing: A mismatch negativity study. *NeuroReport: For Rapid Communication of Neuroscience Research* vol. 18 1709–1712 (2007).
90. Pagnoni, G. & Cekic, M. Age effects on gray matter volume and attentional performance in Zen meditation. *Neurobiol. Aging* **28**, 1623–1627 (2007).
91. Jha, A. P., Krompinger, J. & Baime, M. J. Mindfulness training modifies subsystems of attention. *Cogn. Affect. Behav. Neurosci.* **7**, 109–119 (2007).
92. Bushell, W. C. New beginnings: Evidence that the meditational regimen can lead to optimization of perception, attention, cognition, and other functions. in *Longevity, regeneration, and optimal health: Integrating Eastern and Western perspectives.* 348–361 (Wiley-Blackwell, 2009).
93. Lutz, A. *et al.* Mental training enhances attentional stability: neural and behavioral evidence. *J. Neurosci.* **29**, 13418–13427 (2009).
94. Wenk-Sormaz, H. Meditation can reduce habitual responding. *Advances in Mind-Body Medicine* vol. 21 33–49 (2005).
95. Chambers, R., Lo, B. C. Y. & Allen, N. B. The impact of intensive mindfulness training on attentional control, cognitive style, and affect. *Cognit. Ther. Res.* **32**, 303–322 (2008).
96. Gross, J. J. The Emerging Field of Emotion Regulation: An Integrative Review. *Rev. Gen. Psychol.* **2**, 271–299 (1998).
97. Cosci, F., Pistelli, F., Lazzarini, N. & Carrozzi, L. Nicotine dependence and psychological distress: outcomes and clinical implications in smoking cessation. *Psychol. Res. Behav. Manag.* **4**, 119–128 (2011).
98. Farb, N. A. S. *et al.* Attending to the present: mindfulness meditation reveals distinct neural modes of self-reference. *Soc. Cogn. Affect. Neurosci.* **2**, 313–322 (2007).
99. Taylor, V. A. *et al.* Impact of mindfulness on the neural responses to emotional pictures in experienced and beginner meditators. *Neuroimage* **57**, 1524–1533 (2011).
100. Desbordes, G. *et al.* Effects of mindful-attention and compassion meditation training on amygdala response to emotional stimuli in an ordinary, non-meditative state . *Frontiers in Human Neuroscience* vol. 6 292 (2012).
101. Hölzel, B. K. *et al.* Stress reduction correlates with structural changes in the amygdala. *Soc. Cogn. Affect. Neurosci.* **5**, 11–17 (2010).
102. Barnes, V. A., Treiber, F. A. & Davis, H. Impact of Transcendental Meditation on cardiovascular function at rest and during acute stress in adolescents with high normal blood pressure. *J. Psychosom. Res.* **51**, 597–605 (2001).
103. MacLean, C. R. *et al.* Altered responses of cortisol, GH, TSH and testosterone to acute stress after four months' practice of transcendental meditation (TM). *Ann. N. Y. Acad. Sci.* **746**, 381–384 (1994).

104. Sudsuang, R., Chentanez, V. & Veluvan, K. Effect of Buddhist meditation on serum cortisol and total protein levels, blood pressure, pulse rate, lung volume and reaction time. *Physiol. Behav.* **50**, 543–548 (1991).
105. Carlson, L. E., Speca, M., Faris, P. & Patel, K. D. One year pre-post intervention follow-up of psychological, immune, endocrine and blood pressure outcomes of mindfulness-based stress reduction (MBSR) in breast and prostate cancer outpatients. *Brain. Behav. Immun.* **21**, 1038–1049 (2007).
106. Ortner, C. N. M., Kilner, S. J. & Zelazo, P. D. Mindfulness meditation and reduced emotional interference on a cognitive task. *Motiv. Emot.* **31**, 271–283 (2007).
107. Arch, J. J. & Craske, M. G. Mechanisms of mindfulness: emotion regulation following a focused breathing induction. *Behav. Res. Ther.* **44**, 1849–1858 (2006).
108. Erisman, S. M. & Roemer, L. A preliminary investigation of the effects of experimentally induced mindfulness on emotional responding to film clips. *Emotion* **10**, 72–82 (2010).
109. Goldin, P. R. & Gross, J. J. Effects of mindfulness-based stress reduction (MBSR) on emotion regulation in social anxiety disorder. *Emotion* **10**, 83–91 (2010).
110. Brewer, J. A. *et al.* Mindfulness Training and Stress Reactivity in Substance Abuse: Results from a Randomized, Controlled Stage I Pilot Study. *Subst. Abus.* **30**, 306–317 (2009).
111. Broderick, P. Mindfulness and Coping with Dysphoric Mood: Contrasts with Rumination and Distraction. *Cognit. Ther. Res.* **29**, 501–510 (2005).
112. Campbell-Sills, L., Barlow, D. H., Brown, T. A. & Hofmann, S. G. Effects of suppression and acceptance on emotional responses of individuals with anxiety and mood disorders. *Behav. Res. Ther.* **44**, 1251–1263 (2006).
113. Kuehner, C., Huffziger, S. & Liebsch, K. Rumination, distraction and mindful self-focus: effects on mood, dysfunctional attitudes and cortisol stress response. *Psychol. Med.* **39**, 219–228 (2009).
114. Britton, W. B., Shahar, B., Szepsenwol, O. & Jacobs, W. J. Mindfulness-based cognitive therapy improves emotional reactivity to social stress: results from a randomized controlled trial. *Behav. Ther.* **43**, 365–380 (2012).
115. Bandura, A. *Self-Efficacy: The Exercise of Control*. (1997).
116. Schwarzer, R. *Self-Efficacy: Thought Control of Action*. (1992).
117. Conner, M. & Norman, P. *Predicting Health Behaviour*. (2005).
118. Hogrefe & Huber. The role of self-efficacy in health self-regulation. in *Clinical Handbook of Psychotropic Drugs* 137–152 (2005).
119. Neff, K. The Development and Validation of a Scale to Measure Self-Compassion. *Self Identity* **2**, 223–250 (2003).
120. Neff, K. D., Kirkpatrick, K. L. & Rude, S. S. Self-compassion and adaptive psychological functioning. *J. Res. Pers.* **41**, 139–154 (2007).
121. Neff, K. D. & Vonk, R. Self-compassion versus global self-esteem: two different ways of relating to oneself. *J. Pers.* **77**, 23–50 (2009).
122. Adams, C. E. & Leary, M. R. PROMOTING SELF – COMPASSIONATE ATTITUDES TOWARD EATING AMONG RESTRICTIVE AND GUILTY EATERS. **26**, 1120–1144 (2007).
123. Kelly, A., Zuroff, D., Gilbert, P. & Foa, C. Who Benefits from Training in Self-Compassionate Self-Regulation? A Study of Smoking Reduction. *J. Soc. Clin. Psychol.* **29**, 727–755 (2010).

124. Magnus, C. M. R., Kowalski, K. C. & McHugh, T.-L. F. The role of self-compassion in women's self-determined motives to exercise and exercise-related outcomes. *Self Identity* **9**, 363–382 (2010).
125. Terry, M. L. & Leary, M. R. Self-compassion, self-regulation, and health. *Self Identity* **10**, 352–362 (2011).
126. Nolen-Hoeksema, S., Wisco, B. E. & Lyubomirsky, S. Rethinking Rumination. *Perspect. Psychol. Sci. a J. Assoc. Psychol. Sci.* **3**, 400–424 (2008).
127. Smallwood, J. & Andrews-Hanna, J. Not all minds that wander are lost: the importance of a balanced perspective on the mind-wandering state. *Front. Psychol.* **4**, 441 (2013).
128. Killingsworth, M. A. & Gilbert, D. T. A wandering mind is an unhappy mind. *Science* **330**, 932 (2010).
129. Epel, E. S. *et al.* Wandering Minds and Aging Cells. *Clin. Psychol. Sci.* **1**, 75–83 (2012).
130. Smallwood, J., Fishman, D. J. & Schooler, J. W. Counting the cost of an absent mind: mind wandering as an underrecognized influence on educational performance. *Psychon. Bull. Rev.* **14**, 230–236 (2007).
131. Braboszcz, C. & Delorme, A. Lost in thoughts: neural markers of low alertness during mind wandering. *Neuroimage* **54**, 3040–3047 (2011).
132. Mooneyham, B. W. & Schooler, J. W. The costs and benefits of mind-wandering: A review. *Canadian Journal of Experimental Psychology/Revue canadienne de psychologie expérimentale* vol. 67 11–18 (2013).
133. Brewer, J. A. *et al.* Meditation experience is associated with differences in default mode network activity and connectivity. *Proc. Natl. Acad. Sci. U. S. A.* **108**, 20254–20259 (2011).
134. Kabbat-Zinn, J. *Full Catastrophe Living (Revised Edition): Using the Wisdom of Your Body and Mind to Face Stress, Pain, and Illness*. (2013).
135. Mehling, W. E. *et al.* Body Awareness: a phenomenological inquiry into the common ground of mind-body therapies. *Philos. Ethics. Humanit. Med.* **6**, 6 (2011).
136. Segal, Z., Williams, J. & Teasdale, J. *Mindfulness-Based Cognitive Therapy for Depression*. (2013).
137. Craig, A. D. How do you feel? Interoception: the sense of the physiological condition of the body. *Nat. Rev. Neurosci.* **3**, 655–666 (2002).
138. Craig, A. D. B. The sentient self. *Brain Struct. Funct.* **214**, 563–577 (2010).
139. Damasio, A. & Carvalho, G. B. The nature of feelings: evolutionary and neurobiological origins. *Nat. Rev. Neurosci.* **14**, 143–152 (2013).
140. Herbert, B. M. & Pollatos, O. The body in the mind: on the relationship between interoception and embodiment. *Top. Cogn. Sci.* **4**, 692–704 (2012).
141. Füstös, J., Gramann, K., Herbert, B. M. & Pollatos, O. On the embodiment of emotion regulation: interoceptive awareness facilitates reappraisal. *Soc. Cogn. Affect. Neurosci.* **8**, 911–917 (2013).
142. Interoception and emotion: A neuroanatomical perspective. in *Handbook of emotions, 3rd ed.* (eds. Lewis, M., Haviland-Jones, J. M. & Barrett, L. F.) 272 (The Guilford Press, 2008).
143. Gray, M. A. & Critchley, H. D. Interoceptive basis to craving. *Neuron* **54**, 183–186 (2007).
144. Naqvi, N. H. & Bechara, A. The insula and drug addiction: an interoceptive view of pleasure, urges, and decision-making. *Brain Struct. Funct.* **214**, 435–450 (2010).

145. Addicott, M. A., Sweitzer, M. M., Froeliger, B., Rose, J. E. & McClernon, F. J. Increased Functional Connectivity in an Insula-Based Network is Associated with Improved Smoking Cessation Outcomes. *Neuropsychopharmacology* **40**, 2648–2656 (2015).
146. Farb, N. A. S., Segal, Z. V & Anderson, A. K. Attentional modulation of primary interoceptive and exteroceptive cortices. *Cereb. Cortex* **23**, 114–126 (2013).
147. Lutz, A., Brefczynski-Lewis, J., Johnstone, T. & Davidson, R. J. Regulation of the neural circuitry of emotion by compassion meditation: effects of meditative expertise. *PLoS One* **3**, e1897 (2008).
148. Lutz, A., Greischar, L. L., Perlman, D. M. & Davidson, R. J. BOLD signal in insula is differentially related to cardiac function during compassion meditation in experts vs. novices. *Neuroimage* **47**, 1038–1046 (2009).
149. Lutz, A., McFarlin, D. R., Perlman, D. M., Salomons, T. V & Davidson, R. J. Altered anterior insula activation during anticipation and experience of painful stimuli in expert meditators. *Neuroimage* **64**, 538–546 (2013).
150. Lamm, C. & Singer, T. The role of anterior insular cortex in social emotions. *Brain Struct. Funct.* **214**, 579–591 (2010).
151. Nakata, H., Sakamoto, K. & Kakigi, R. Meditation reduces pain-related neural activity in the anterior cingulate cortex, insula, secondary somatosensory cortex, and thalamus. *Front. Psychol.* **5**, 1489 (2014).
152. Gard, T. *et al.* Pain attenuation through mindfulness is associated with decreased cognitive control and increased sensory processing in the brain. *Cereb. Cortex* **22**, 2692–2702 (2012).
153. Allen, M. *et al.* Cognitive-affective neural plasticity following active-controlled mindfulness intervention. *J. Neurosci.* **32**, 15601–15610 (2012).
154. Farb, N. A. S., Segal, Z. V & Anderson, A. K. Mindfulness meditation training alters cortical representations of interoceptive attention. *Soc. Cogn. Affect. Neurosci.* **8**, 15–26 (2013).
155. Luders, E. *et al.* The unique brain anatomy of meditation practitioners: alterations in cortical gyrification. *Front. Hum. Neurosci.* **6**, 34 (2012).
156. Lazar, S. W. *et al.* Meditation experience is associated with increased cortical thickness. *Neuroreport* **16**, 1893–1897 (2005).
157. Hölzel, B. K. *et al.* Investigation of mindfulness meditation practitioners with voxel-based morphometry. *Soc. Cogn. Affect. Neurosci.* **3**, 55–61 (2008).
158. Grant, J. A., Courtemanche, J. & Rainville, P. A non-elaborative mental stance and decoupling of executive and pain-related cortices predicts low pain sensitivity in Zen meditators. *Pain* **152**, 150–156 (2011).
159. Marchand, W. R. Neural mechanisms of mindfulness and meditation: Evidence from neuroimaging studies. *World J. Radiol.* **6**, 471–479 (2014).
160. Lutz, J. *et al.* Mindfulness and emotion regulation--an fMRI study. *Soc. Cogn. Affect. Neurosci.* **9**, 776–785 (2014).
161. Fox, K. C. R. *et al.* Is meditation associated with altered brain structure? A systematic review and meta-analysis of morphometric neuroimaging in meditation practitioners. *Neurosci. Biobehav. Rev.* **43**, 48–73 (2014).
162. Zeidan, F. *et al.* Brain mechanisms supporting the modulation of pain by mindfulness meditation. *J. Neurosci.* **31**, 5540–5548 (2011).
163. Haase, L. *et al.* Mindfulness-based training attenuates insula response to an aversive

interoceptive challenge. *Soc. Cogn. Affect. Neurosci.* **11**, 182–190 (2016).

164. Segal, Z. V & Walsh, K. M. Mindfulness-based cognitive therapy for residual depressive symptoms and relapse prophylaxis. *Curr. Opin. Psychiatry* **29**, 7–12 (2016).

165. Moyers, T. B., Houck, J., Glynn, L. H., Hallgren, K. A. & Manuel, J. K. A randomized controlled trial to influence client language in substance use disorder treatment. *Drug and Alcohol Dependence* vol. 172 43–50 (2017).

166. Gawande, R. *et al.* Mindfulness Training Enhances Self-Regulation and Facilitates Health Behavior Change for Primary Care Patients: a Randomized Controlled Trial. *J. Gen. Intern. Med.* **34**, 293–302 (2019).

167. Roos, C. R. & Witkiewitz, K. A contextual model of self-regulation change mechanisms among individuals with addictive disorders. *Clin. Psychol. Rev.* **57**, 117–128 (2017).

168. Slep, A. *et al.* Targeting Corrosive Couple Conflict and Parent-Child Coercion to Impact Health Behaviors and Regimen Adherence. *National Institutes of Health* (2015).

169. Leonard, K. E. & Eiden, R. D. Marital and family processes in the context of alcohol use and alcohol disorders. *Annu. Rev. Clin. Psychol.* **3**, 285–310 (2007).

170. Markoff, L. S., Reed, B. G., Fallot, R. D., Elliott, D. E. & Bjelajac, P. Implementing Trauma-Informed Alcohol and Other Drug and Mental Health Services for Women: Lessons Learned in a Multisite Demonstration Project. *American Journal of Orthopsychiatry* vol. 75 525–539 (2005).

171. Jennings, A. *Models for Developing Trauma-Informed Behavioral Health Systems and Trauma- Specific Services*. (2007).

172. Ecker, A. H. & Hundt, N. Posttraumatic Stress Disorder in Opioid Agonist Therapy: A Review. *Psychol. Trauma Theory, Res. Pract. Policy* **10**, 636–642 (2018).

173. Ortiz, R. & Sibinga, E. M. The Role of Mindfulness in Reducing the Adverse Effects of Childhood Stress and Trauma. *Child. (Basel, Switzerland)* **4**, 16 (2017).

174. Garland, E. L. Restructuring reward processing with Mindfulness-Oriented Recovery Enhancement: novel therapeutic mechanisms to remediate hedonic dysregulation in addiction, stress, and pain. *Ann. N. Y. Acad. Sci.* **1373**, 25–37 (2016).

175. Bowen, S. & Marlatt, A. Surfing the urge: brief mindfulness-based intervention for college student smokers. *Psychol. Addict. Behav. J. Soc. Psychol. Addict. Behav.* **23**, 666–671 (2009).

176. Witkiewitz, K. & Bowen, S. Depression, craving, and substance use following a randomized trial of mindfulness-based relapse prevention. *J. Consult. Clin. Psychol.* **78**, 362–374 (2010).

177. Bean, M. K., Mazzeo, S. E., Stern, M., Bowen, D. & Ingersoll, K. A values-based Motivational Interviewing (MI) intervention for pediatric obesity: study design and methods for MI Values. *Contemp. Clin. Trials* **32**, 667–674 (2011).

178. Miller, W. & Baca, J. *Quantum Change: When Epiphanies and Sudden Insights Transform Ordinary Lives*. (Guilford Press, 2001).

179. Markland, D., Ryan, R. M., Tobin, V. J. & Rollnick, S. Motivational Interviewing and Self-Determination Theory. *J. Soc. Clin. Psychol.* **24**, 811–831 (2005).

180. Lovas, D. A., Lutz, J. & Schuman-Olivier, Z. Meditation and Medication—What About a Middle Path? *JAMA Psychiatry* **73**, 1294–1295 (2016).

181. Vivmont, C. Mindfulness May Help Patients Reduce Suboxone Dose, Pilot Program Suggests. *Partnership for Drug Free Kids* (2017).

182. Bagley, S. M., Hadland, S. E., Carney, B. L. & Saitz, R. Addressing Stigma in Medication

Treatment of Adolescents With Opioid Use Disorder. *Journal of addiction medicine* vol. 11 415–416 (2017).

183. Woods, J. & Joseph, H. Stigma from the Viewpoint of the Patient. *J. Addict. Dis.* **34**, (2015).

184. Curry, S., Marlatt, G. A. & Gordon, J. R. Abstinence violation effect: validation of an attributional construct with smoking cessation. *J. Consult. Clin. Psychol.* **55**, 145–149 (1987).

185. Hilbert, A. *et al.* Self-Compassion as a Resource in the Self-Stigma Process of Overweight and Obese Individuals. *Obes. Facts* **8**, 293–301 (2015).

186. Heath, P. J., Brenner, R. E., Vogel, D. L., Lannin, D. G. & Strass, H. A. Masculinity and barriers to seeking counseling: The buffering role of self-compassion. *J. Couns. Psychol.* **64**, 94–103 (2017).

187. Gawande, R. *et al.* Insurance-Reimbursable Mindfulness for Safety-Net Primary Care Patients: a Pilot Randomized Controlled Trial. *Mindfulness (N. Y).* **10**, 1744–1759 (2019).

188. Nielsen, L. *et al.* The NIH Science of Behavior Change Program: Transforming the science through a focus on mechanisms of change. *Behav. Res. Ther.* **101**, 3–11 (2018).

189. Achtyes, E. D. *et al.* Validation of computerized adaptive testing in an outpatient nonacademic setting: The VOCATIONS trial. *Psychiatr. Serv.* **66**, 1091–1096 (2015).

190. Gibbons, R. D. *et al.* Development of a Computerized Adaptive Test Suicide Scale—The CAT-SS. *J. Clin. Psychiatry* **78**, 1376–1382 (2017).

191. Copersino, M. L. *et al.* Rapid cognitive screening of patients with substance use disorders. *Exp. Clin. Psychopharmacol.* **17**, 337–344 (2009).

192. Ewert, V. *et al.* Determination of MoCA Cutoff Score in Patients with Alcohol Use Disorders. *Alcohol. Clin. Exp. Res.* **42**, 403–412 (2018).

193. Chapman, J. E. *et al.* Comparing face-to-face and videoconference completion of the Montreal Cognitive Assessment (MoCA) in community-based survivors of stroke. *J. Telemed. Telecare* 1357633X19890788 (2019) doi:10.1177/1357633X19890788.

194. Demarzo, M. *et al.* Efficacy of 8- and 4-Session Mindfulness-Based Interventions in a Non-clinical Population: A Controlled Study. *Front. Psychol.* **8**, 1343 (2017).

195. Greeson, J. M., Juberg, M. K., Maytan, M., James, K. & Rogers, H. A randomized controlled trial of Koru: a mindfulness program for college students and other emerging adults. *J Am. Coll. Health* **62**, 222–233 (2014).

196. Cormack, D., Jones, F. W. & Maltby, M. A “Collective Effort to Make Yourself Feel Better”: The Group Process in Mindfulness-Based Interventions. *Qual. Health Res.* **28**, 3–15 (2017).

197. Rooks, J., Morrison, A. B., Goolsarran, M., Rogers, S. & Jha, A. P. _We Are Talking About Practice_: the Influence of Mindfulness vs. Relaxation Training on Athlete�_ Attention and Well-Being over High-Demand Intervals. *J. Cogn. Enhanc.* **1**, 141–153 (2017).

198. Wendt, D. C. & Gone, J. P. Group Therapy for Substance Use Disorders: A Survey of Clinician Practices. *J. Groups Addict. Recover.* **12**, 243–259 (2017).

199. Wendt, D. C. & Gone, J. P. Complexities with group therapy facilitation in substance use disorder specialty treatment settings. *J. Subst. Abuse Treat.* **88**, 9–17 (2018).

200. Efird, J. Blocked Randomization with Randomly Selected Block Sizes. *International Journal of Environmental Research and Public Health* vol. 8 (2011).

201. Crane, R. S. & Kuyken, W. The Mindfulness-Based Interventions: Teaching Assessment

Criteria (MBI:TAC): reflections on implementation and development. *Curr. Opin. Psychol.* **28**, 6–10 (2019).
202. Black, D. S. Mindfulness-based interventions: an antidote to suffering in the context of substance use, misuse, and addiction. *Subst. Use Misuse* **49**, 487–491 (2014).
203. Nasreddine, Z. S. *et al.* The Montreal Cognitive Assessment, MoCA: A Brief Screening Tool for Mild Cognitive Impairment. *J. Am. Geriatr. Soc.* **53**, 695–699 (2005).
204. Mitchell, S. G. *et al.* The Use of Technology in Participant Tracking and Study Retention: Lessons Learned From a Clinical Trials Network Study. *Subst. Abus.* **36**, 420–426 (2015).
205. Petry, N. M. & Bohn, M. J. Fishbowls and candy bars: using low-cost incentives to increase treatment retention. *Sci. Pract. Perspect.* **2**, 55–61 (2003).
206. Walker, R. *et al.* Disseminating contingency management to increase attendance in two community substance abuse treatment centers: lessons learned. *J. Subst. Abuse Treat.* **39**, 202–209 (2010).
207. Atlas.ti Version 8.4, Berlin GER. (2019).
208. Lindahl, J. R., Fisher, N. E., Cooper, D. J., Rosen, R. K. & Britton, W. B. *The varieties of contemplative experience: A mixed-methods study of meditation-related challenges in Western Buddhists*. *PLoS ONE* vol. 12 (2017).
209. Jones, H. E. *et al.* Neonatal abstinence syndrome after methadone or buprenorphine exposure. *N. Engl. J. Med.* **363**, 2320–2331 (2010).
210. College of Obstetricians and Gynocologists. Opioid Use and Opioid Use Disorder in Pregnancy. (2017).
211. Debelak, K., Morrone, W. R., O'Grady, K. E. & Jones, H. E. Buprenorphine + naloxone in the treatment of opioid dependence during pregnancy-initial patient care and outcome data. *Am. J. Addict.* **22**, 252–254 (2013).
212. Wiegand, S. L. *et al.* Buprenorphine and naloxone compared with methadone treatment in pregnancy. *Obstet. Gynecol.* **125**, 363–368 (2015).
213. Nguyen, L. *et al.* Treating women with opioid use disorder during pregnancy in Appalachia: Initial neonatal outcomes following buprenorphine + naloxone exposure. *Am. J. Addict.* **27**, 92–96 (2018).
214. Jumah, N. A. *et al.* Observational study of the safety of buprenorphine+naloxone in pregnancy in a rural and remote population. *BMJ Open* **6**, e011774 (2016).
215. Dhillon, A., Sparkes, E. & Duarte, R. V. Mindfulness-Based Interventions During Pregnancy: a Systematic Review and Meta-analysis. *Mindfulness (N. Y).* **8**, 1421–1437 (2017).
216. Lander, L. R., Marshalek, P. & Sullivan, C. R. Medication-Assisted Treatment for Pregnant Women: An Interdisciplinary Group Based Model. *J. Groups Addict. Recover.* **11**, 182–193 (2016).
217. Hsu, Y.-J., Marsteller, J. A., Kachur, S. G. & Fingerhood, M. I. Integration of Buprenorphine Treatment with Primary Care: Comparative Effectiveness on Retention, Utilization, and Cost. *Popul. Health Manag.* **22**, 292–299 (2019).
218. Alford, D. P. *et al.* Collaborative care of opioid-addicted patients in primary care using buprenorphine: five-year experience. *Arch. Intern. Med.* **171**, 425–431 (2011).
219. Stein, M. D., Cioe, P. & Friedmann, P. D. Brief report: Buprenorphine retention in primary care. *J. Gen. Intern. Med.* **20**, 1038–1041 (2005).
220. Neumann, A. M., Blondell, R. D., Azadfard, M., Nathan, G. & Homish, G. G. Primary care patient characteristics associated with completion of 6-month buprenorphine

treatment. *Addict. Behav.* **38**, 2724—2728 (2013).

221. Streck, J. M., Ochalek, T. A., Badger, G. J. & Sigmon, S. C. Interim buprenorphine treatment during delays to comprehensive treatment: Changes in psychiatric symptoms. *Exp. Clin. Psychopharmacol.* **26**, 403–409 (2018).

222. Marsch, L. A. *et al.* A randomized controlled trial of buprenorphine taper duration among opioid-dependent adolescents and young adults. *Addiction* **111**, 1406–1415 (2016).

223. Liang, K.-Y. & Zeger, S. L. Longitudinal Data Analysis Using Generalized Linear Models. *Biometrika* **73**, 13–22 (1986).

224. Zeger, S. L. & Liang, K.-Y. Longitudinal Data Analysis for Discrete and Continuous Outcomes. *Biometrics* **42**, 121–130 (1986).

225. Cui, J. & Feng, L. Correlation Structure and Model Selection for Negative Binomial Distribution in GEE. *Commun. Stat. - Simul. Comput.* **38**, 190–197 (2008).

226. Rubin, D. B. & Schenker, N. Multiple imputation in health-care databases: an overview and some applications. *Stat. Med.* **10**, 585–598 (1991).

227. Schalet, B. D., Cook, K. F., Choi, S. W. & Cella, D. Establishing a common metric for self-reported anxiety: linking the MASQ, PANAS, and GAD-7 to PROMIS Anxiety. *J. Anxiety Disord.* **28**, 88–96 (2014).

228. Teresi, J. A. *et al.* Measurement Equivalence of the Patient Reported Outcomes Measurement Information System® (PROMIS®) Pain Interference Short Form Items: Application to Ethnically Diverse Cancer and Palliative Care Populations. *Psychol Test Assess Model.* **58**, 309–352 (2016).

229. Little, R. J. A. & Robin, D. B. *Statistical Analysis with Missing Data*. (Wiley, 2002).

230. Benjamini, Y. & Hochberg, Y. Controlling the False Discovery Rate: A Practical and Powerful Approach to Multiple Testing. *J. R. Stat. Soc. Ser. B* **57**, 289–300 (1995).

231. Cao, J. & Zhang, S. Multiple comparison procedures. *JAMA* **312**, 543–544 (2014).

232. Glickman, M. E., Rao, S. R. & Schultz, M. R. False discovery rate control is a recommended alternative to Bonferroni-type adjustments in health studies. *J. Clin. Epidemiol.* **67**, 850–857 (2014).

233. Wahbeh, H., Goodrich, E., Goy, E. & Oken, B. S. Mechanistic Pathways of Mindfulness Meditation in Combat Veterans With Posttraumatic Stress Disorder. *J. Clin. Psychol.* **72**, 365–383 (2016).

234. Cohen, S., Kamarck, T. & Mermelstein, R. A global measure of perceived stress. *J Heal. Soc Behav* **24**, 385–396 (1983).

235. Boyd, J. E., Adler, E. P., Otilingam, P. G. & Peters, T. Internalized Stigma of Mental Illness (ISMI) scale: a multinational review. *Compr. Psychiatry* **55**, 221–231 (2014).

236. Kamaradova, D. *et al.* Connection between self-stigma, adherence to treatment, and discontinuation of medication. *Patient Prefer. Adherence* **10**, 1289–1298 (2016).

237. Gyawali, S. *et al.* Perceived stigma and its correlates among treatment seeking alcohol and opioid users at a tertiary care centre in India. *Asian J. Psychiatr.* **37**, 34–37 (2018).

238. Sarkar, S. *et al.* Internalized stigma among patients with substance use disorders at a tertiary care center in India. *J. Ethn. Subst. Abuse* **18**, 345–358 (2019).

239. Pratscher, S. D., Wood, P. K., King, L. A. & Bettencourt, B. A. Interpersonal Mindfulness: Scale Development and Initial Construct Validation. *Mindfulness (N. Y).* **10**, 1044–1061 (2019).

240. Finkelhor, D., Shattuck, A., Turner, H. & Hamby, S. Improving the adverse childhood experiences study scale. *JAMA Pediatr.* **167**, 70–75 (2013).

241. Health NIo. Phenx Toolkit. https://www.phenxtoolkit.org/index.php (2018).
242. Christensen, D. R. *et al.* Adding an Internet-delivered treatment to an efficacious treatment package for opioid dependence. *J. Consult. Clin. Psychol.* **82**, 964–972 (2014).
243. Beardsley, K., Wish, E. D., Fitzelle, D. B., O'Grady, K. & Arria, A. M. Distance traveled to outpatient drug treatment and client retention. *J. Subst. Abuse Treat.* **25**, 279–285 (2003).
244. Norton, P. J. & Kazantzis, N. Dynamic relationships of therapist alliance and group cohesion in transdiagnostic group CBT for anxiety disorders. *J. Consult. Clin. Psychol.* **84**, 146–155 (2016).
245. Beck, A. & SR. Beck Anxiety Inventory manual. (1993).
246. Weinstein, Z. M. *et al.* Long-term retention in Office Based Opioid Treatment with buprenorphine. *J. Subst. Abuse Treat.* **74**, 65–70 (2017).
247. Kumar, N., Stowe, Z. N., Han, X. & Mancino, M. J. Impact of early childhood trauma on retention and phase advancement in an outpatient buprenorphine treatment program. *Am. J. Addict.* **25**, 542–548 (2016).
248. Rosenbaum, P. R. & Rubin, D. B. Constructing a Control Group Using Multivariate Matched Sampling Methods That Incorporate the Propensity Score. *Am. Stat.* **39**, 33–38 (1985).
249. Stuart, E. A. Matching Methods for Causal Inference: A Review and a Look Forward. *Stat. Sci.* **25**, 1–21 (2010).
250. Gibbons, R. D. *et al.* Development of a computerized adaptive test for depression. *Arch. Gen. Psychiatry* **69**, 1104–1112 (2012).
251. Gibbons, R. D. *et al.* Development of the CAT-ANX: a computerized adaptive test for anxiety. *Am. J. Psychiatry* **171**, 187–94 (2014).
252. Gibbons, R. D., Weiss, D. J., Frank, E. & Kupfer, D. Computerized Adaptive Diagnosis and Testing of Mental Health Disorders. *Annu. Rev. Clin. Psychol.* **12**, 83–104 (2016).
253. Gibbons, R. D. & deGruy, F. V. Without Wasting a Word: Extreme Improvements in Efficiency and Accuracy Using Computerized Adaptive Testing for Mental Health Disorders (CAT-MH). *Current Psychiatry Reports* (2019) doi:10.1007/s11920-019-1053-9.
254. Felitti, V. J. *et al.* Relationship of childhood abuse and household dysfunction to many of the leading causes of death in adults. The Adverse Childhood Experiences (ACE) Study. *Am. J. Prev. Med.* **14**, 245–258 (1998).
255. Teresi, J. A., Ocepek-Welikson, K., Kleinman, M., Ramirez, M. & Kim, G. Measurement Equivalence of the Patient Reported Outcomes Measurement Information System® (PROMIS®) Anxiety Short Forms in Ethnically Diverse Groups. **58**, 183–219 (2016).
256. Chen, C. X. *et al.* Estimating minimally important differences for the PROMIS pain interference scales: results from 3 randomized clinical trials. *Pain* **159**, 775–782 (2018).
257. Beck, A. T., Epstein, N., Brown, G. & Steer, R. A. An inventory for measuring clinical anxiety: psychometric properties. *J. Consult. Clin. Psychol.* **56**, 893–897 (1988).
258. Covideo, L. Droplr. (2020).
259. Sullivan, M. J. L., Bishop, S. R. & Pivik, J. The Pain Catastrophizing Scale: Development and validation. *Psychol. Assess.* **7**, 524–532 (1995).
260. McHugh, R. K. *et al.* Assessing craving and its relationship to subsequent prescription opioid use among treatment-seeking prescription opioid dependent patients. *Drug Alcohol Depend.* **145**, 121–126 (2014).

261. Weiss, R. D. *et al.* The relationship between cocaine craving, psychosocial treatment, and subsequent cocaine use. *Am. J. Psychiatry* **160**, 1320–1325 (2003).
262. Gámez, W., Chmielewski, M., Kotov, R., Ruggero, C., Suzuki, N. & Watson, D. The Brief Experiential Avoidance Questionnaire: Development and initial validation. *Psychol. Assess.* **26**, 35–45 (2014).
263. Gratz, K. L. & Roemer, L. Multidimensional Assessment of Emotion Regulation and Dysregulation: Development, Factor Structure, and Initial Validation of the Difficulties in Emotion Regulation Scale. *J. Psychopathol. Behav. Assess.* **26**, 41–54 (2004).
264. Bjureberg, J. *et al.* Development and Validation of a Brief Version of the Difficulties in Emotion Regulation Scale : The DERS-16. **38**, 284–296 (2016).
265. Raes, F., Pommier, E., Neff, K. D. & Van Gucht, D. Construction and factorial validation of a short form of the Self-Compassion Scale. *Clin. Psychol. Psychother.* **18**, 250–255 (2011).
266. Bornemann, B. & Singer, T. Taking time to feel our body: Steady increases in heartbeat perception accuracy and decreases in alexithymia over 9 months of contemplative mental training. *Psychophysiology* **54**, 469–482 (2017).
267. Whitehead, R., Bates, G., Elphinstone, B., Yang, Y. & Murray, G. Letting Go of Self: The Creation of the Nonattachment to Self Scale. *Front. Psychol.* **9**, 2544 (2018).
268. Yang, Y., Fletcher, K., Michalak, E. E. & Murray, G. An investigation of self-compassion and nonattachment to self in people with bipolar disorder. *J. Affect. Disord.* **262**, 43—48 (2020).
269. Smart, L. M., Peters, J. R. & Baer, R. A. Development and Validation of a Measure of Self-Critical Rumination. *Assessment* **23**, 321–332 (2016).
270. Soler, J. *et al.* Assessing Decentering: Validation, Psychometric Properties, and Clinical Usefulness of the Experiences Questionnaire in a Spanish Sample. *Behav. Ther.* (2014) doi:10.1016/j.beth.2014.05.004.
271. Baer, R. A. *et al.* Construct Validity of the Five Facet Mindfulness Questionnaire in Meditating and Nonmeditating Samples. *Assessment* **15**, 329–42 (2008).
272. MacKenzie, K. R. Measurement of Group Climate. *Int. J. Group Psychother.* **31**, 287–295 (1981).
273. Carmody, J. & Baer, R. A. Relationships between mindfulness practice and levels of mindfulness, medical and psychological symptoms and well-being in a mindfulness-based stress reduction program. *J. Behav. Med.* **31**, 23–33 (2008).
274. Harris, P. A. *et al.* Research Electronic Data Capture (REDCap) - A metadata-driven methodology and workflow process for providing translational research informatics support. *J Biomed Inf.* **42**, 377–381 (2009).
275. Crane, R. S. *et al.* Development and Validation of the Mindfulness-Based Interventions – Teaching Assessment Criteria (MBI:TAC). *Assessment* **20**, 681–688 (2013).
276. Chawla, N. *et al.* The mindfulness-based relapse prevention adherence and competence scale: development, interrater reliability, and validity. *Psychother. Res.* **20**, 388–397 (2010).
277. Germer, C. K. & Neff, K. D. Self-compassion in clinical practice. *J. Clin. Psychol.* **69**, 856–867 (2013).
