# Supplementary material for: Mindfulness Training vs Recovery Support for Opioid Use, Craving, and Anxiety During Buprenorphine Treatment: A Randomized Clinical Trial
Source: JAMA Netw Open. 2025 Jan 21;8(1):e2454950. doi: 10.1001/jamanetworkopen.2024.54950 (PMC11751747; doi:10.1001/jamanetworkopen.2024.54950)
Supplement: Supplement 2. — eFigure 1. PROMIS-Anxiety by Study Week and Group eTable 1. Abstinence Outcomes by Arm With Additional Covariates Using Imputation eTable 2. Secondary and Exploratory Outcomes by Group With Additional Covariates in Sensitivity Analysis Using Imputation eFigure 2. Opioid Craving Scale Total by Study Week and Group With Imputation eResults 1. Sensitivity Analysis eTable 3. Abstinence Outcomes by Arm in Sensitivity Analysis With Additional Covariates eTable 4. Secondary and Exploratory Outcomes by Group With Additional Covariates in Sensitivity Analysis eResults 2. Adverse Events eFigure 3. Geographic Distribution of Randomized Participants [file jamanetwopen-e2454950-s002.pdf]

Supplementary Online Content

Schuman-Olivier Z, Goodman H, Rosansky J, et al. Mindfulness training vs recovery support for opioid use, craving, and anxiety during buprenorphine treatment: a randomized clinical trial. *JAMA Netw Open*. 2025;8(1):e2454950.
doi:10.1001/jamanetworkopen.2024.54950

eFigure 1. PROMIS-Anxiety by Study Week and Group

eTable 1. Abstinence Outcomes by Arm With Additional Covariates Using Imputation

eTable 2. Secondary and Exploratory Outcomes by Group With Additional Covariates in Sensitivity Analysis Using Imputation

eFigure 2. Opioid Craving Scale Total by Study Week and Group With Imputation

eResults 1. Sensitivity Analysis

eTable 3. Abstinence Outcomes by Arm in Sensitivity Analysis With Additional Covariates

eTable 4. Secondary and Exploratory Outcomes by Group With Additional Covariates in Sensitivity Analysis

eResults 2. Adverse Events

eFigure 3. Geographic Distribution of Randomized Participants

This supplementary material has been provided by the authors to give readers additional information about their work.

© 2025 Schuman-Olivier Z et al.

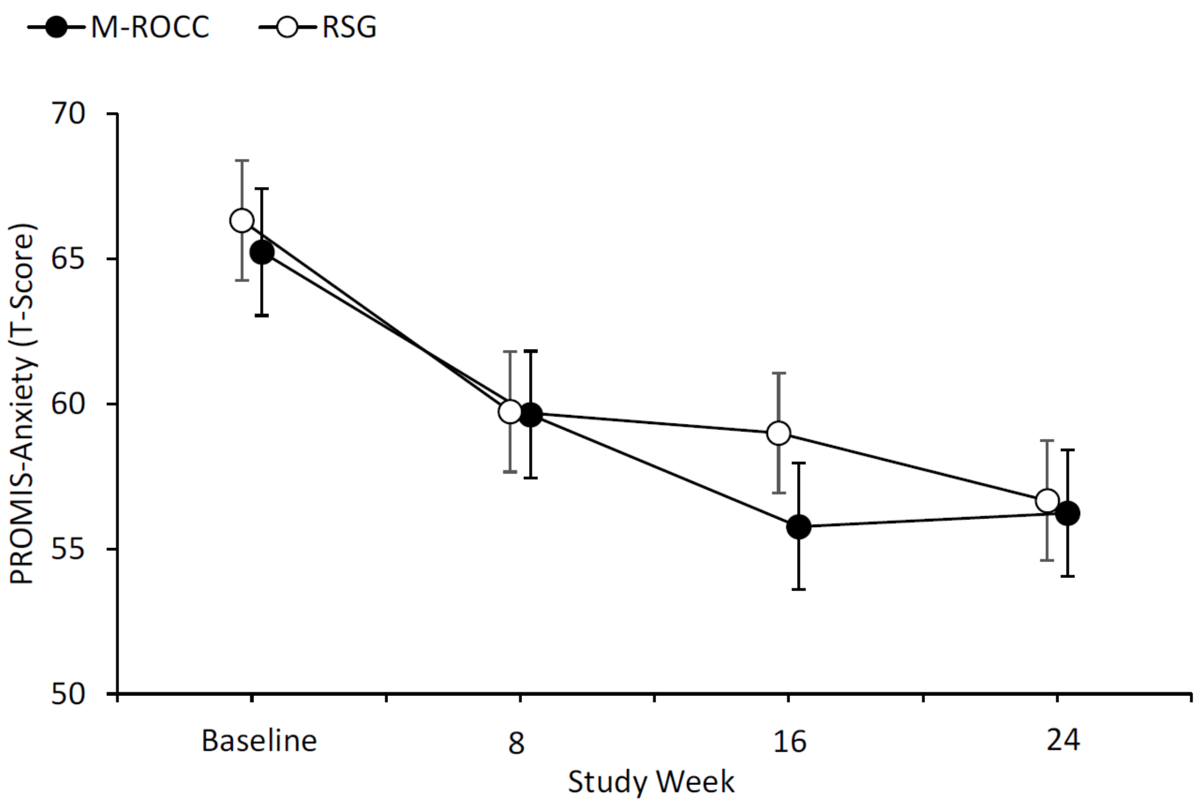

eFigure 1. PROMIS-Anxiety by Study Week and Group

© 2025 Schuman-Olivier Z et al.

Measures	Weeks 13 to 24		Difference in Week 13 to 24 for M-ROCC Relative to RSG		
	Percentage of Non-Abstinent Periods [%]	95% CI	Diff [%]	95% CI	P
Primary Measure					
**Non-Abstinent Periods of Opioids**					
RSG	22.7	[8.3, 62.1]	-	-	-
M-ROCC	24	[9.1, 62.9]	1.2	[-13.4, 15.8]	0.87
Secondary Measures					
**Non-Abstinent Periods of Cocaine**					
RSG	16.7	[10.8, 30.5]	-	-	-
M-ROCC	18.1	[11.9, 35]	1.4	[-8.6, 23.8]	0.85
**Non-Abstinent Periods of Benzodiazepines**					
RSG	31.9	[18.3, 43.1]	-	-	-
M-ROCC	29.7	[17, 39.6]	-2.1	[-14.9, 20.4]	0.81

eTable 1. Abstinence Outcomes by Arm with additional covariates using imputation

© 2025 Schuman-Olivier Z et al.

Measures	Study Group by Week Interaction		Week 24 - Week 0			Difference in Week 24 - Week 0 for M-ROCC Relative to RSG		
	F [df1,df2]	Adj P[#]	Diff	95% CI	ES	Diff	95% CI	ES
Secondary								
**PROMIS-Anxiety**								
RSG	1.6 [3, 218]	0.29	-9.9	[-12, -7.7]	-1.2	-	-	-
M-ROCC			-9.3	[-12.1, -6.6]	-1.1	0.5	[-2.9, 3.9]	0.1
Exploratory [pre-specified]								
**Opioid Craving**								
RSG	11.5 [1, 264]	<0.01	-1.4	[-1.9, -0.9]	-0.7	-	-	-
M-ROCC			-2.4	[-3.1, -1.8]	-1.2	-1.0	[-1.9, -0.2]	-0.5

[#]The p-values for the Study Group by Week Interaction were adjusted using the Benjamini-Hochberg false discovery rate procedure to account for multiple comparisons across this family of two outcome measures according to the Cao et al method.[38,39] Diff: Difference. ES: Effect size.

eTable 2. Secondary and Exploratory Outcomes by Group with additional covariates in sensitivity analysis using imputation

© 2025 Schuman-Olivier Z et al.

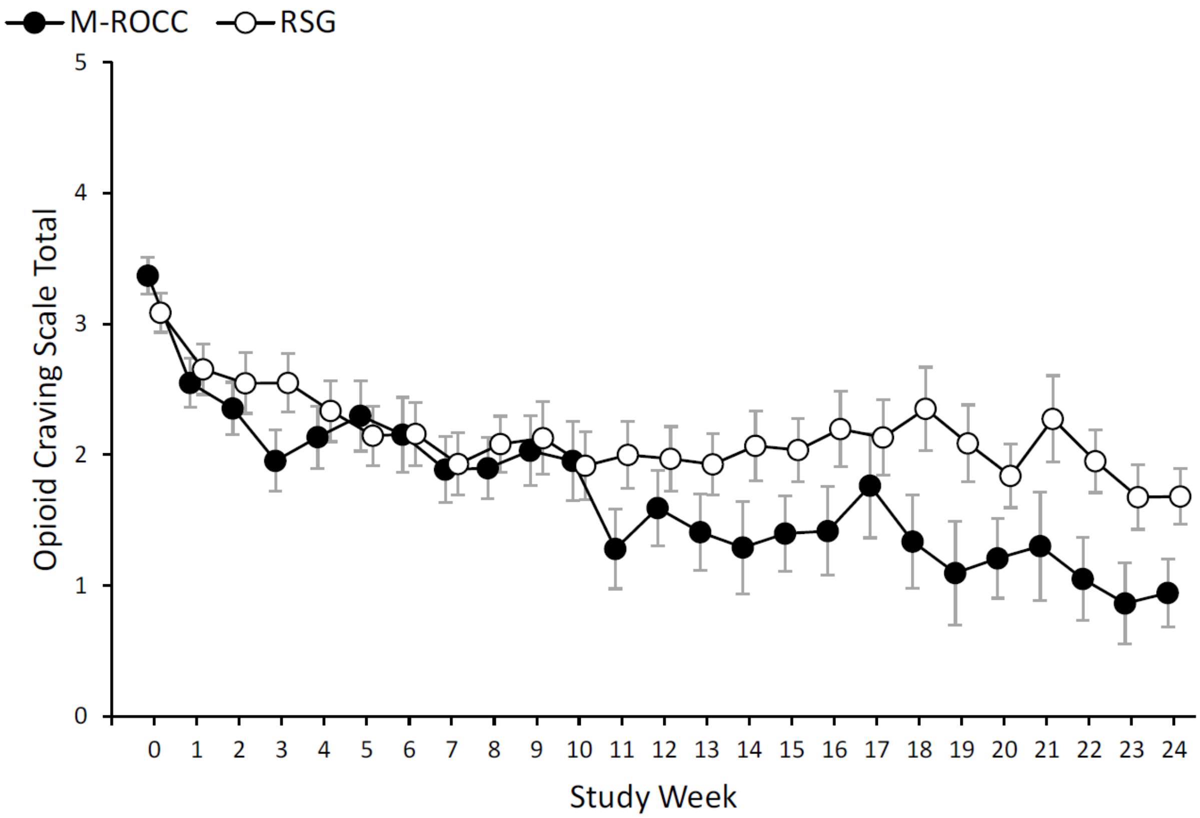

The figure shows the marginal estimates of the (0-10) Opioid Craving Scale score by study group and week, with the standard error of the estimate shown as vertical bars. Examining variation in opioid craving over time by study group, the interaction term for study group by study week, when added to the model in addition to the main effects, was found to be significant.

eFigure 2. Opioid Craving Scale Total by Study Week and Group with Imputation

© 2025 Schuman-Olivier Z et al.

eResults 1. Sensitivity Analysis

Of 196 participants enrolled, 102 (52%) provided a complete clinical outcome survey at week 24. No covariates differed (*p*<0.100) between those with and without missing clinical survey data at week 24. Overall continuation in the study post week 8 was 53% (103 participants), with no covariates found to be associated. Retention, defined as having a prescription buprenorphine dose reported at week 24 was 51%, with no covariates found to be associated. Post-8-week engagement, defined as the number of sessions attended post week 8 averaged 4.7 sessions, where those aged at least 40 years attended more sessions (mean=5.7) than those younger than 40 years (3.9) (*p*=0.1).

Examining covariates that differed (*p*<0.15) according to an interaction term between study arms and continuation post week 8, we found an association between study group and participants who identified either as Hispanic, non-white, or had English as their second language. Where, 70% of participants in the RSG arm continued post week 8, while only 6% of those in the M-ROCC group chose to continue (*p*=0.018).

For average sessions attended post week 8, attendance differed between study group and participants with four or more adverse childhood experiences (ACEs) and past mindfulness experience. Participants with four or more ACEs attended an average of 2.6 and 6.2 sessions in the M-ROCC and RSG groups, respectively. The participants with less than four ACEs attended an average of 5 and 5.6 sessions in the M-ROCC and RSG groups, respectively (*p*=0.080). Participants with past mindfulness experience attended an average of 2.2 and 7.6 sessions post week 8 in the M-ROCC and RSG groups, respectively. Those with less mindfulness experience attended an average of 3.7 and 5.6 sessions in the M-ROCC and RSG groups, respectively (*p*=0.085).

© 2025 Schuman-Olivier Z et al.

Measures	Weeks 13 to 24		Difference in Week 13 to 24 for M-ROCC Relative to RSG		
	Percentage of Non-Abstinent Periods (%)	95% CI	Diff (%)	95% CI	P
Primary Measure					
**Non-Abstinent Periods of Opioids**					
RSG	13.5	[8.5, 18.5]	-	-	-
M-ROCC	12.4	[5.3, 19.5]	-1.1	[-10.1, 7.8]	0.807
Secondary Measures					
**Non-Abstinent Periods of Cocaine**					
RSG	1.8	[0.0, 4.7]	-	-	-
M-ROCC	6.8	[0.6, 13.0]	5.0	[-2.9, 13.0]	0.214
**Non-Abstinent Periods of Benzodiazepines**					
RSG	21.3	[13.8, 28.8]	-	-	-
M-ROCC	20.4	[11.8, 29.0]	0.9	[-12.6, 10.7]	0.870

eTable 3. Abstinence Outcomes by Arm in Sensitivity Analysis with Additional Covariates

© 2025 Schuman-Olivier Z et al.

Measures	Study Group by Week Interaction		Week 24 - Week 0			Difference in Week 24 - Week 0 for M-ROCC Relative to RSG		
	Chi-sq, df	Adj P[#]	Diff	95% CI	ES	Diff	95% CI	ES
Secondary								
**PROMIS-Anxiety**								
RSG	4.5, 3	0.318	-9.6	[-11.5, -7.7]	-1.3	-	-	-
M-ROCC			-8.7	[-11.4, -6.0]	-1.2	0.9	[-2.4, 4.2]	0.1
Exploratory (pre-specified)								
**Opioid Craving**								
RSG	56.3, 24	0.001	-1.4	[-2.0, -0.9]	-0.8	-	-	-
M-ROCC			-2.3	[-2.9, -1.7]	-1.3	-0.9	[-1.7, -0.1]	-0.5

[#]The p-values for the Study Group by Week Interaction were adjusted using the Benjamini-Hochberg false discovery rate procedure to account for multiple comparisons across this family of two outcome measures according to the Cao et al method.[38,39] Diff: Difference. ES: Effect size.

eTable 4. Secondary and Exploratory Outcomes by Group with additional covariates in sensitivity analysis

© 2025 Schuman-Olivier Z et al.

eResults 2. Adverse Events

Among M-ROCC participants, 29/98 individuals reported 40 AEs (0.41 per person), including 39 unrelated to the protocol. This was not significantly different from RSG, in which 21/98 participants reported 26 AEs (0.27 per person) (p=0.09). No moderate-to-severe (0/40) or severe (0/9) AEs (e.g., injuries, grief-related symptoms, infections/COVID-19) were related to the interventions.

© 2025 Schuman-Olivier Z et al.

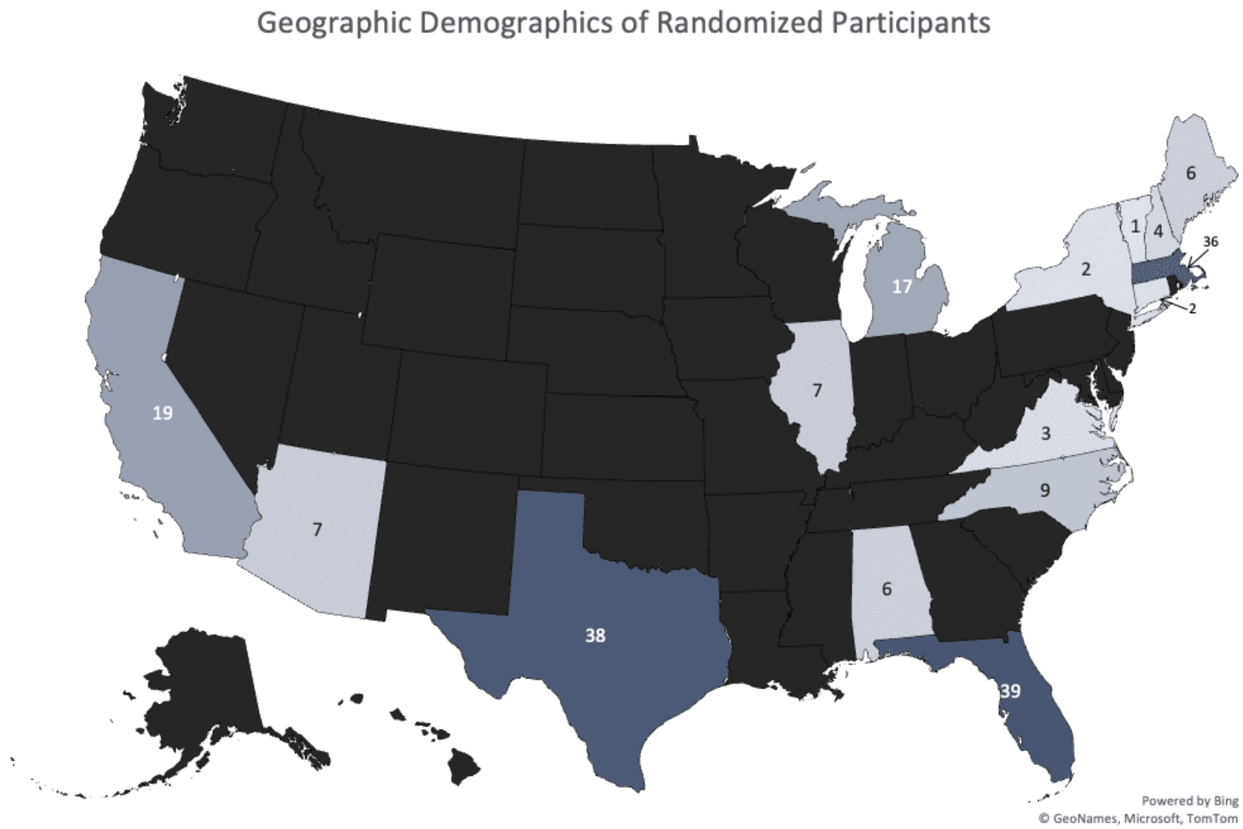

eFigure 3. Geographic Distribution of Randomized Participants

© 2025 Schuman-Olivier Z et al.
