# Supplementary material for: Mindfulness Training vs Recovery Support for Opioid Use, Craving, and Anxiety During Buprenorphine Treatment: A Randomized Clinical Trial
Source: JAMA Netw Open. 2025 Jan 21;8(1):e2454950. doi: 10.1001/jamanetworkopen.2024.54950 (PMC11751747; doi:10.1001/jamanetworkopen.2024.54950)
Supplement: Supplement 3. — Data Sharing Statement [file jamanetwopen-e2454950-s003.pdf]

Data Sharing Statement

Schuman-Olivier. Mindfulness Training vs Recovery Support for Opioid Use, Craving, and Anxiety During Buprenorphine Treatment. *JAMA Netw Open*. Published January 21, 2025. doi:10.1001/jamanetworkopen.2024.54950

Data
Additional Information: Trial Registration: NCT04278586
Data available: Yes
Data types: Deidentified participant data, Data dictionary
How to access data: https://search.vivli.org/studyDetails/fromSearch/0a33769b-cb02-42b7-8078-14cde19186f4
When available: With publication

Supporting Documents
Document types: Statistical/analytic code, Informed consent form
How to access documents: https://search.vivli.org/studyDetails/fromSearch/0a33769b-cb02-42b7-8078-14cde19186f4
When available: With publication

Additional Information
Who can access the data: Data will be available to researchers upon submitting a request on the Vivli website to access the shared study data and will be available through the NIH HEAL Data Ecosystem.
Types of analyses: Primary, Secondary and Pre-specified Exploratory Analyses will be made available. Any analysis proposal sent by a researcher looking to access other data shared on the Vivli data repository will be reviewed by the study team.
Mechanisms of data availability: Data will be available with a data access agreement with Vivli. After approval of the manuscript for submission then the deidentified data set and analyses code will be uploaded to Vivli prior to publication or by September 1st, whichever is first in accordance with the NIH HEAL Data Ecosystem requirements.
Any additional restrictions: n/a
